# Galectin-8 binds to the Farnesylated C-terminus of K-Ras4B and Modifies Ras/ERK Signaling and Migration in Pancreatic and Lung Carcinoma Cells

**DOI:** 10.3390/cancers12010030

**Published:** 2019-12-20

**Authors:** Christopher Meinohl, Sarah J. Barnard, Karin Fritz-Wolf, Monika Unger, Andreea Porr, Marisa Heipel, Stefanie Wirth, Johannes Madlung, Alfred Nordheim, Andre Menke, Katja Becker, Klaudia Giehl

**Affiliations:** 1 Signal Transduction of Cellular Motility, Internal Medicine V, Justus Liebig University Giessen, D-35392 Giessen, Germany; Christopher.Meinohl@innere.med.uni-giessen.de (C.M.); Sarah.Barnard@web.de (S.J.B.); marisa_heipel@web.de (M.H.); Stefanie.Wirth@innere.med.uni-giessen.de (S.W.); 2 Max Planck Institute for Medical Research, D-69120 Heidelberg, Germany; Karin.Fritz-Wolf@mpimf-heidelberg.mpg.de; 3 Biochemistry and Molecular Biology, Justus Liebig University Giessen, D-35392 Giessen, Germany; Katja.Becker@ernaehrung.uni-giessen.de; 4 Institute of Pharmacology and Toxicology, University of Ulm, D-89069 Ulm, Germany; mojo.unger@web.de; 5 Internal Medicine I, University of Ulm, D-89069 Ulm, Germany; andreeaporr@yahoo.com; 6 Interfaculty Institute of Cell Biology, Proteome Center Tübingen, University of Tübingen, D-72076 Tübingen, German; johannes.madlung@uni-tuebingen.de (J.M.); alfred.nordheim@uni-tuebingen.de (A.N.); 7 Interfaculty Institute of Cell Biology, Unit of Molecular Biology, University of Tübingen, D-72076 Tübingen, Germany; 8 Molecular Oncology of Solid Tumors, Justus-Liebig-University Giessen, D-35392 Giessen, Germany; andre.menke@innere.med.uni-giessen.de

**Keywords:** K-Ras4B, Galectin-8, signal transduction, ERK, lung and pancreatic carcinoma, migration, proliferation

## Abstract

K-Ras is the most prominent driver of oncogenesis and no effective K-Ras inhibitors have been established despite decades of intensive research. Identifying new K-Ras-binding proteins and their interaction domains offers the opportunity for defining new approaches in tackling oncogenic K-Ras. We have identified Galectin-8 as a novel, direct binding protein for K-Ras4B by mass spectrometry analyses and protein interaction studies. Galectin-8 is a tandem-repeat Galectin and it is widely expressed in lung and pancreatic carcinoma cells. siRNA-mediated depletion of Galectin-8 resulted in increased K-Ras4B content and ERK1/2 activity in lung and pancreatic carcinoma cells. Moreover, cell migration and cell proliferation were inhibited by the depletion of Galectin-8. The K-Ras4B–Galectin-8 interaction is indispensably associated with the farnesylation of K-Ras4B. The lysine-rich polybasic domain (PBD), a region that is unique for K-Ras4B as compared to H- and N-Ras, stabilizes the interaction and accounts for the specificity. Binding assays with the deletion mutants of Galectin-8, comprising either of the two carbohydrate recognition domains (CRD), revealed that K-Ras4B only interacts with the N-CRD, but not with the C-CRD. Structural modeling uncovers a potential binding pocket for the hydrophobic farnesyl chain of K-Ras4B and a cluster of negatively charged amino acids for interaction with the positively charged lysine residues in the N-CRD. Our results demonstrate that Galectin-8 is a new binding partner for K-Ras4B and it interacts via the N-CRD with the farnesylated PBD of K-Ras, thereby modulating the K-Ras effector pathways as well as cell proliferation and migration.

## 1. Introduction

Monomeric GTPases of the Ras family act as molecular switches of signaling pathways, thereby modulating many aspects of physiologic and pathophysiologic cell behavior, including cell proliferation, differentiation, survival, and apoptosis, as well as cell migration and invasion. Moreover, they are the most frequently mutated human oncogenes in cancer and are most prominently associated with human malignancies. The three ubiquitously expressed Ras genes (*HRAS*, *KRAS*, and *NRAS*) encode for the four isoforms H-Ras, N-Ras, and the two splice-variants K-Ras4A and K-Ras4B. Missense gain of function mutations, especially in codon 12, 13, or 61, render Ras constitutively active in its GTP-bound state. These mutations are found in 27% of all human cancer, and *KRAS* is most commonly affected in pancreatic (with around 90%), colon (40%), and lung (25%) adenocarcinoma [1]. Ras proteins share more than 90% sequence homology within their first 168/169 amino acids (aa), but they differ in their last 23/24 aa at the C-terminus, designated as the hypervariable region (HVR). For K-Ras 4A and 4B the HVR is encoded by the alternatively spliced fourth exon. K-Ras4A is less abundant and far less studied than K-Ras4B [2,3].

Ras must be associated with membranes for the activation of downstream signaling pathways, such as the mitogen-activated protein kinase (MAPK) or the phosphoinositide 3-kinase α (PI3Kα) pathway. Therefore, all of the Ras isoforms are carboxy-methylated and farnesylated at the C-terminal cysteine. H-Ras, N-Ras, and K-Ras4A are further palmitoylated at one or two cysteine residues in the HVR, providing the second signal for stable interaction with the plasma membrane and for recycling processes [4,5,6]. K-Ras4B is not palmitoylated, but it exhibits a stretch of lysines that constitute the so-called polybasic domain (PBD) for electrostatic interaction with acidic lipids at the inner leaflet of the plasma membrane [7,8,9]. K-Ras4B dissociates from the plasma membrane via protein kinase C (PKC)-catalyzed phosphorylation of serine 181 within the PBD [10]. Membrane-bound K-Ras4B is in a dynamic exchange with a cytoplasmic pool, where it is bound to chaperones, such as phosphodiesterase-δ (PDEδ), thus shielding the hydrophobic farnesyl lipid [11,12]. The HVR modification is also important for segregating Ras isoforms into distinct, non-overlapping microdomains in the inner leaflet of the plasma membrane. H-Ras and N-Ras localize in cholesterol-rich liquid-ordered lipid rafts and non-raft structures, depending on the bound nucleotide, with H-Ras.GDP in lipid raft and H-Ras.GTP in non-raft structures and N-Ras in opposite directions [13,14,15]. Spatial segregation into nanometer-sized domains, designated as nanoclusters, which are essential for high-fidelity signal transduction, further enhances the compartmentalization [16]. Extensive studies of John Hancock’s group suggest that approximately 56% of Ras molecules exist as freely diffusing monomers or dimers. The remaining are ordered into nanoclusters that contain ~6 Ras molecules. The formation of Ras dimers, oligomers, and nanoclusters not only depends on the interplay of the lipid anchor and HVR, as well as the G domain of Ras with the specific membrane composition, but also on auxiliary scaffold proteins, such as galectins [7,14,17]. 

Mammalian Galectins comprise a family of 15 carbohydrate-binding proteins that are involved in many physiological functions, such as apoptosis, immune response, inflammation, angiogenesis, adhesion, and migration, as well as cell transformation and tumor growth [18]. All Galectin family members exhibit a conserved β-galactoside binding site within the common ~130 aa carbohydrate recognition domains (CRDs) for carbohydrate-dependent interactions with extracellular glycoconjugates. They also interact with cytosolic and nuclear proteins in a carbohydrate-independent fashion. Galectins are classified into: prototype galectins with one CRD, such as Galectin-1; chimera-type Galectin-3 with one C-terminal CRD and a proline- and glycine-rich amino acid chain; and, tandem-repeat-type galectins, including Galectin-8, having two CRDs that are connected by a hinge region of variable length [19,20,21]. In contrast to Galectin-8, Galectin-1 and 3 have been demonstrated to interact with Ras proteins. Experiments overexpressing Galectin-1 showed enhanced H-Ras.GTP nanoclustering, and the activation of H-Ras led to an accumulation of Galectin-1 at H-Ras.GTP nanoclusters [22]. Furthermore, Shalom-Feuerstein demonstrated the association of Galectin-3 with K-Ras.GTP [23]. The binding to Ras involves the farnesyl moiety of the GTP-bound Ras proteins with a direct binding of Galectin-3 to K-Ras4B [24]. The binding of Galectin-1 to H-Ras might be more indirect involving the Ras-binding domain (RBD) of Ras effectors, e.g., cRaf1 [25]. Interaction of active K-Ras4B with Galectin-3 reduces the dissociation of K-Ras4B from the plasma membrane by stabilizing the nanocluster-trapped K-Ras.GTP [26]. The overexpression of Galectin-3 in cancer cells increased K-Ras4B-induced signal transduction [27,28]. The CRD domain of Galectin-3 harbors a hydrophobic pocket that binds the farnesyl chain of K-Ras4B [23], as supported by the fact that farnesylthiosalicylic acid (FTS/Salirasib), which mimics the carboxy-terminal farnesyl cysteine carboxymethyl ester of K-Ras4B dislodges the active Ras protein from cellular membranes by disrupting the interaction with Galectin-3 [29,30]. Therefore, Galectin-3 might not only act as a guanosine nucleotide dissociation inhibitor (GDI)-like chaperone, but also regulates GTP-bound K-Ras4B membrane association, which supports K-Ras oncogenic signaling. In all, Galectin-3 and Galectin-1 support high fidelity and prolonged Ras signaling downstream to Raf or PI3-kinase, and they also control Ras-regulated tumor suppressor, e.g., Bcl2 or β-catenin, thereby supporting tumor survival [21,31,32].

We performed co-precipitation analyses to identify new binding partners for K-Ras4B, which might serve as a new target for therapeutic intervention, since K-Ras4B is most prominently associated with malignant cancer progression and still hardly druggable. Here, we report that the tandem-repeat Galectin-8 is a new specific and direct interaction partner of K-Ras4B. We analyzed the interaction of K-Ras4B and Galectin-8 and its impact on signal transduction, as well as cell proliferation and migration in pancreatic and lung carcinoma cells. Moreover, we defined the structural components that mediate this specific binding and presented a structural model of how the farnesyl moiety of K-Ras4B interacts with Galectin-8. 

## 2. Results

### 2.1. Identification of Galectin-8 as a Novel Binding Protein for K-Ras

Co-precipitation experiments were performed with recombinant, post-translationally modified Ras isoforms to identify new binding partners for oncogenic K-Ras. Farnesylated, human influenza hemagglutinin (HA)-tagged K-Ras (G12V), H-Ras (G12V), and N-Ras (G12V) were purified from the membrane-containing fraction of baculovirus-infected *Sf*9 insect cells, immobilized on anti-HA para-magnetic beads, and subsequently incubated with PANC-1 cell lysate to precipitate Ras isoform-specific binding proteins. K-Ras4B is referred to as K-Ras since the expression of K-Ras4A is almost undetectable in human lung and pancreatic carcinoma cell lines [33].

**Figure 1:**
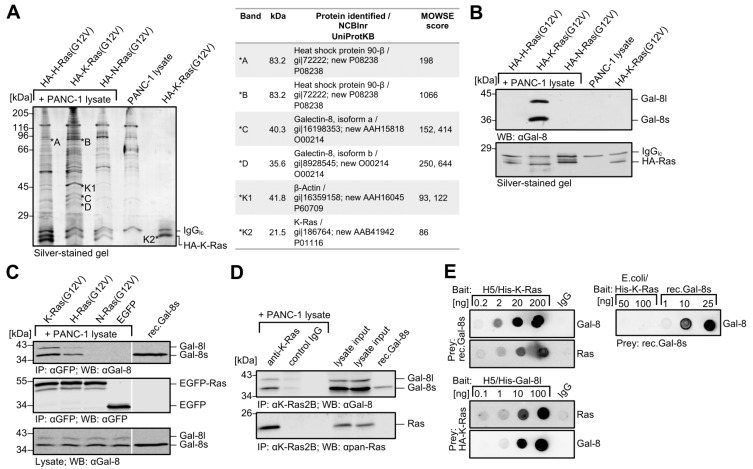
Identification of Galectin-8 as K-Ras interaction partner. (**A**)Identification of Galectin-8 by mass spectrometry. Purified, post-translationally modified HA-H-Ras (G12V), HA-K-Ras (G12V), and HA-N-Ras (G12V) (500 ng) immobilized on anti-HA µMacs beads were incubated with PANC-1 lysate and precipitates were separated by SDS-PAGE. As controls, PANC-1 lysate were incubated with anti-HA microbeads only [PANC-1 lysate] and HA-K-Ras (G12V) were coupled microbeads only [HA-K-Ras (G12V)]. Peptides from the indicated proteins (*A–*D, and *K1, *K2 as controls, * = extracted protein band), extracted from the silver-stained gel were analyzed by ESI-MS⁄MS mass spectrometry (*n* = 3). The table shows the identified proteins. (**B**) Detection of precipitated Galectin-8. HA-Ras interacting proteins were prepared as outlined in (**A**) and analyzed by western blot. The upper panel shows the detection of Galectin-8 while using anti-Gal-8 antibody and the lower panel the corresponding silver-stained gel of one third of the eluted proteins to control for equal precipitation of Ras isoforms (*n* = 3). (**C**) Interaction of EGFP-tagged Ras isoforms with Galectin-8. EGFP-tagged K-Ras (G12V), H-Ras (G12V), or N-Ras (G12V) were transiently expressed in HEK293 cell, immunoprecipitated using anti-GFP µMacs beads and then incubated with PANC-1 lysate for interaction assays. The precipitates were analyzed by western blotting and rec.Gal-8s (30 ng) was applied as a control. Upper panel: detection of co-precipitated Galectin-8; middle panel: precipitated EGFP Ras isoforms; lower panel: endogenous Galectin-8 in 80 µg of the PANC-1 lysates (*n* = 4). (**D**) Co-immunoprecipitation of endogenous K-Ras with Galectin-8. Endogenous K-Ras was precipitated from PANC-1 cell lysate (8 mg) while using anti-K-Ras2B antibody [anti-K-Ras] and protein A µMacs beads. Precipitation with rabbit IgG [control IgG] was performed as control. The precipitates were analyzed by western blotting. The amount of Galectin-8 and K-Ras was controlled in 1/150th of the lysate [lysate input], and 10 ng rec.Gal-8s served as a control. Upper panel: Co-precipitated Galectin-8; lower panel: precipitated K-Ras (*n* = 3). (**E**) The interaction of purified K-Ras and Galectin-8. The indicated amounts of His-K-Ras (G12V) [H5/His-K-Ras] or H5/His-Gal-8l, each expressed in H5 insect cells (left panel), and indicated amounts of *E. coli*-derived His-K-Ras [E. coli/His-K-Ras] and *E. coli*-derived Galectin-8 short [rec.Gal-8s] (right panel) were dotted as baits onto nitrocellulose membranes. Rabbit IgG served as control. The membranes were either incubated with 1 µg of rec.Gal-8s or with 1 µg HA-K-Ras (G12V) from *Sf*9 insect cells as a prey. Bound (upper blots) or spotted (lower blots) proteins were detected by the indicated antibodies (*n* = 2).

Figure 1A shows a representative silver-stained SDS-polyacrylamide gel with the characteristic band pattern for each Ras isoform precipitate. Mass spectrometry identified the marked protein bands. Bands C and D, which were exclusively detected in HA-K-Ras (G12V) co-precipitates, were identified as Galectin-8 isoform a and Galectin-8 isoform b, respectively (table in Figure 1A). HSP90 (bands A and B) was detected in HA-H-Ras (G12V) and HA-K-Ras (G12V) co-precipitates and not further considered in this study. Fig. 1B shows that these two Galectin-8 isoforms were verified as Galectin-8 by western blotting while using a specific Galectin-8 antibody. The upper band corresponded to the longer isoform a, named Galectin-8 long (Gal-8l), and the lower band to the shorter isoform b, named Galectin-8 short (Gal-8s). The origin of these two proteins was further characterized at the mRNA level in PANC-1 and six other pancreatic carcinoma cell lines. RT-PCR amplified two cDNA variants from all cell lines. The longer cDNA with 1145 bp (from ATG to stop codon) (NM_006499.3, NCBI, LGAL8S variant 1), encoded for the Galectin-8 long isoform with 359 amino acids (aa). The shorter cDNA comprised 956 bp (NM_201543.1, NCBI, LGAL8S variant 2), encoding for Galectin-8 short with 317 aa. Sequence analyses showed that the two variants differ with regard to the expression of exon 11, which encodes for 42 aa, being located in the hinge region of Galectin-8 between the two CRDs. Galectin-8 cDNAs were both cloned in expression vectors and used for ectopic expression within this work. Supplementary Figure S1 shows the endogenous expression of Galectin-8 long and short, as well as Galectin-1 and Galectin-3, in nine pancreatic (Supplementary Figure S1A) and eleven lung carcinoma cell lines (Supplementary Figure S1B). Galectin-8 short is more prominent than Galectin-8 long in nearly all analyzed cell lines. The lung adenocarcinoma cell line H1299 lacks detectable amounts of Galectin-8 and HEK293 kidney cells contain no detectable amounts of the three Galectins and, therefore, were used for ectopic expression experiments.

K-Ras (G12V), H-Ras (G12V), and N-Ras (G12V) were transiently expressed as EGFP fusion proteins in HEK293 cells, precipitated by anti-GFP µMACS beads, and incubated with PANC-1 cell lysates to precipitate endogenous Galectin-8 long and short from these lysates to investigate whether Galectin-8 also interacted differentially with EGFP-Ras isoforms. The western blot in Figure 1C demonstrates that Galectin-8 isoforms were both markedly precipitated with EGFP-K-Ras (G12V) and to a lower extent with EGFP-H-Ras (G12V) (lanes 1 and 2). Galectin-8 hardly ever interacted with EGFP-N-Ras (G12V) (lane 3) and it did not interact with EGFP (lane 4). Co-immunoprecipitation analyses in which both the EGFP-Ras isoforms and Galectin-8 long or short were co-expressed in HEK293 cells confirmed these results were confirmed and the anti-GFP antibody precipitated EGFP-Ras proteins (Supplementary Figure S2A). A quantitative analysis of the interaction of Galectin-8 from PANC-1 cells with ectopically expressed EGFP-Ras isoforms is presented in Supplementary Figure S2B. EGFP-H-Ras (G12V) showed an interaction of 33.78 ± 15.14% and EGFP-N-Ras (G12V) of 3.44 ± 5.12% (densitometric analyses of *n* = 4) when setting the interaction of Galectin-8 short with EGFP-K-Ras (G12V) to 100% as reference. Note that we could detect a very weak binding of Galectin-3 (black bar) and an apparent binding of Galectin-1 (grey bar) to EGFP-K-Ras (G12V) in the same precipitate.

**Figure 2:**
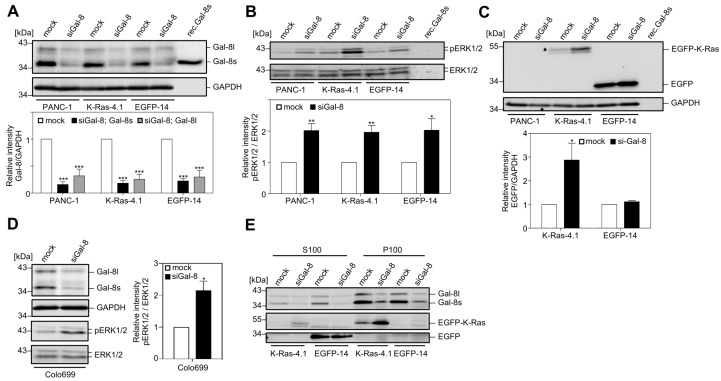
Activation of ERK1/2 by downregulation of Galectin-8. (**A**–**C**). PANC-1 cells and cell clones stably expressing EGFP-K-Ras(G12V) [K-Ras-4.1] or EGFP [EGFP-14] were transiently transfected with Gal-8 [siGal-8] or control [mock] siRNA and lysed 72 h after transfection. The expression of Galectin-8, pERK1/2, ERK1/2, EGFP proteins, and GAPDH was investigated by western blot. 15 ng of rec.Gal-8s was applied as a control. The western blots in (**A**) show the amount of Galectin-8 (upper panel) and of GAPDH as a loading control (lower panel). The bar graph shows the mean ± SEM of densitometric analyses of Galectin-8 long and short in relation to GAPDH, normalized to the mock-transfected control. The values are given as relative intensity (*** *p* ≤ 0.001, *n* = 6). (**B**) The phosphorylation of ERK1/2 at Thr202/Tyr204 (upper panel) and the amount of total ERK1/2 (lower panel) was detected simultaneously in Western blot analysis using the Odyssey Infrared Imaging System. The bar graph shows the densitometric analysis of the ratio of pERK1/2 to ERK1/2 normalized to the control (mean ± SEM; * *p* ≤ 0.05; ** *p* ≤ 0.01, *n* = 6). (**C**) Expression of EGFP-K-Ras and EGFP was evaluated using anti-GFP antibody. GAPDH (lower panel) was determined as a loading control. The diagram shows the densitometric analysis as a ratio of EGFP/GAPDH normalized to the mock-transfected control (mean ± SEM; * *p* ≤ 0.05, *n* = 6). (**D**) Depletion of Galectin-8 in lung carcinoma cells. Colo699 cells were treated as in (A). The western blots show the amount of Galectin-8 and GAPDH (upper panel) and pERK1/2 and ERK1/2 (lower panel). The diagram shows the densitometric analysis of the ratio of pERK1/2 to ERK1/2 (mean ± SEM; * *p* ≤ 0.05, *n* = 4). (**E**) Subcellular localization of EGFP-K-Ras (G12V) and Galectin-8. To determine whether enhanced ERK phosphorylation is associated with an enhanced amount of active, membrane-associated EGFP-K-Ras (G12V), EGFP-14 and K-Ras-4.1 PANC-1 cells were treated as in (**A**). Lysates were fractionated into soluble [S100] (cytosolic) and particulate [P100] (membranous) fractions and they were analyzed by western blot (S100: 50 µg, P100: 25 µg). The upper blot shows the distribution and depletion of Galectin-8 in both fractions, the middle panel displays the distribution of EGFP-K-Ras, especially in the P100 fraction, and the lower panel shows the enrichment of EGFP in the S100 fraction (*n* = 4).

Co-immunoprecipitation assays were performed to detect K-Ras-Galectin-8 interaction in living cells since all these analyses were carried out with ectopically expressed Ras proteins. PANC-1 cells, which express considerable amounts of both Galectin-8 isoforms as well as oncogenic and wildtype K-Ras4B [34] were used. Endogenous K-Ras was precipitated while using a K-Ras4B specific antibody and endogenous Galectin-8 was clearly co-precipitated (Figure 1D). Moreover, cell fractionation assays (see Figure 2E) and immunofluorescence studies (see Supplementary Figure S4A) revealed that Galectin-8 located to the cytoplasm and markedly to the membrane, where it co-localized with K-Ras. Subsequently, we investigated whether purified K-Ras and purified Galectin-8 directly interact with each other in immunodot blot experiments. Therefore, purified His-tagged K-Ras (G12V) and His-tagged Galectin-8 long proteins, expressed as modified proteins in baculovirus-infected insect cells, and unmodified His-tagged K-Ras as well as Galectin-8 short (rec.Gal-8s) from *E. coli* were used as bait or prey, respectively. Figure 1E demonstrates direct interaction of these proteins. The upper blot in each panel shows the detection of the bound and thus interacting prey protein, and the lower blot the amount of the spotted bait protein. Recombinant Gal-8s interacted with post-translationally modified K-Ras expressed in insect cells (upper panel left). Interestingly, it did not with unmodified K-Ras from *E. coli* (right panel), where rec.Gal-8s was spotted in increasing amounts as a control. In the inverse experiment, substantial amounts of K-Ras bound to insect-expressed Gal-8l when Gal-8l was spotted and incubated with posttranslationally modified K-Ras (G12V) (lower panel left). Taken together, these results demonstrate that both isoforms of Galectin-8 represent new direct binding proteins for oncogenic K-Ras.

### 2.2. Downregulation of Galectin-8 Affects K-Ras and ERK1/2

The effects of Galectin-8 silencing on the activity of the mitogen-activated protein kinases ERK1/2 were analyzed following the siRNA approach while using a pool of four siRNAs targeting different sequences of Galectin-8 to characterize signal transduction mechanisms regulated by Galectin-8. Figure 2A shows that the application of these siRNAs resulted in a >70% depletion of both Galectin-8 isoforms in PANC-1, EGFP-K-Ras (G12V), and EGFP-expressing PANC-1 cells. PANC-1 cells stably expressing EGFP-K-Ras (G12V) (clone 4.1 and 4.4) (34) were used to detect the effects of Galectin-8 depletion, particularly on EGFP-K-Ras. The silencing of Galectin-8 leading to a two-fold upregulation of the phosphorylation of ERK1/2 in all analyzed cell clones, without affecting the total amount of ERK1/2, is of note (Figure 2B). In the case of EGFP-K-Ras (G12V) cells, a significant increase in the amount of EGFP-K-Ras (2.87 ± 0.72-fold) as compared to the mock-transfected control accompanied this upregulation. The amount of EGFP did not change (1.12 ± 0.05-fold) (Figure 2C). The deletion of Galectin-8 in Colo699 lung carcinoma cells, which also express Galectin-8, -3, and -1 as PANC-1 cells (Supplementary Figure S1B), produced a similar increase in ERK1/2 phosphorylation, as shown in Figure 2D. A549 lung carcinoma cells also exhibited a 1.7-fold increased ERK phosphorylation after Galectin-8 depletion, demonstrating a cell line independent effect. The analysis of the subcellular localization of Galectin-8 in PANC-1 cell clones revealed that a substantial amount is localized in the P100 particulate, membrane-containing fraction. EGFP-K-Ras (G12V) is particularly localized in the P100 fraction, whereas EGFP is only restricted to the cytosolic fraction (Figure 2E, upper panel). Moreover, siRNA-mediated deletion of Galectin-8 caused an increase of EGFP-K-Ras (G12V), especially in the P100 fraction (Figure 2E, lanes 5 and 6), which represents K-Ras molecules that are capable of inducing signal transduction leading to ERK phosphorylation. The determination of the activity of EGFP-K-Ras (G12V) after Galectin-8 silencing by GST-RBD pull-down assays confirmed a marked increase in the amount of the GTP-bound and, thus, active protein concomitant with an increase in the total amount of EGFP-K-Ras (G12V). 

**Figure 3:**
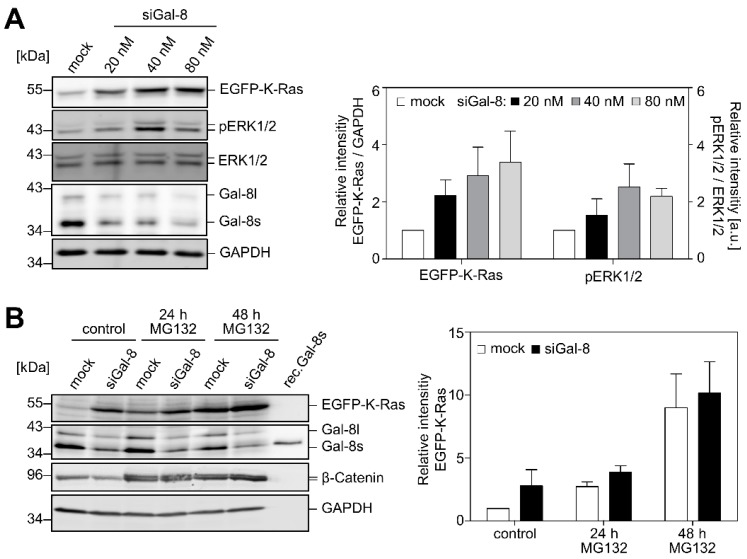
Depletion of Galectin-8 enhances the amount of EGFP-K-Ras and affects ERK. (**A**) PANC-1/EGFP-K-Ras (G12V)-4.1 cells were transiently transfected with increasing amounts of Gal-8 siRNA [siGal-8] or control siRNA [mock]. Protein expression of ERK1/2, pERK1/2, Galectin-8 and EGFP-K-Ras was determined by western blot. The western blots (left) and the diagram (right) show the increasing amounts of EGFP-K-Ras and pERK1/2 as a function of the concentration of siGal-8. The bar graph shows the mean ± SEM of the densitometric analysis (left: EGFP-K-Ras, right: pERK1/2) as relative intensity normalized to the mock-transfected control (*n* = 3). (**B**) Increase of EGFP-K-Ras by MG132. PANC-1/EGFP-K-Ras (G12V)-4.1 cells transfected with siGal-8 or control siRNA were left untreated or additionally treated for 24 h or for 48 h with 5 µM MG132. Lysates were prepared 72 h after transfection and then analyzed by western blot. 30 ng of rec.Gal-8s was applied as a control. Detection of β-catenin served as a control for the activity of MG132. GAPDH served as a loading control. The bar graph shows the mean ± SEM of the densitometric analysis of EGFP-K-Ras normalized to the mock-transfected control without MG132 (*n* = 3).

The increase in the amount of EGFP-K-Ras (G12V) and in the activity of ERK1/2 was dependent on the Galectin-8 siRNA concentration, as shown in Figure 3A. Based on these observations, we hypothesized that Galectin-8 might function as a novel regulator for maintaining a certain steady-state level of K-Ras within the cell. The mode of K-Ras degradation is controversial. Lu et al. reported that K-Ras undergoes lysosomal degradation [35], but Koo et al. described proteasome-mediated degradation [36]. K-Ras exhibits a half-life of >12 h under normal physiological conditions [37]. Mock and siGal-8 treated PANC-1/EGFP-K-Ras (G12V) cells were treated for 24 h or 48 h with the proteasome inhibitor MG132 to address the issue if inhibition of the proteasome with MG132 affects EGFP-K-Ras or Galectin-8 protein stability. As shown in Figure 3B, MG132 treatment increases the amount of EGFP-K-Ras (white columns) in a time-dependent manner as compared to control. Within 24 h of treatment, this effect was equal or slightly higher than treatment with siGal-8 alone (black bar [control] as compared to white bar [24 h MG132]). The treatment with MG132 for 48 h resulted in a marked and similar increase in EGFP-K-Ras in both mock and siGal-8-treated cells. MG132 did not affect the amount of Galectin-8, whereas the level of β-catenin clearly increased, as expected from numerous studies in the literature.

### 2.3. Impact of Galectin-8 Deletion on Cell Migration and Proliferation

**Figure 4:**
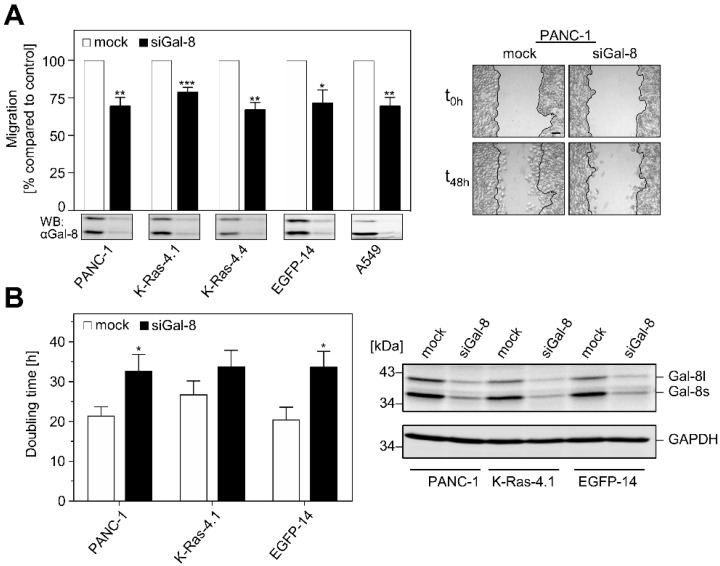
Effects of Galectin-8 depletion on migration and proliferation. (**A**) Wounding assay. PANC-1, EGFP- and EGFP-K-Ras (G12V) expressing cells [PANC-1, EGFP-14, K-Ras-4.1, K-Ras-4.4], and A549 lung cells were transfected with siGal-8 or control siRNA [mock]. After treatment with mitomycin-C, the confluent cell layer was scratched, and images of six randomly chosen points were recorded every 3 h for 48 h. The velocity [µm/h] was calculated, normalized to the mock transfected control of each cell line, and presented as the mean ± SEM (* *p* ≤ 0.05; ** *p* ≤ 0.01; *** *p* ≤ 0.001, *n* = 3–4). The insets below show Galectin-8 depletion 48 h after wounding. The image on the right shows representative phase contrast images of PANC-1 cells. The line represents the wound at t_0h_, bar: 100 µm. (**B**) Proliferation. PANC-1 cells and cell clones [PANC-1, EGFP-14, K-Ras-4.1] were transfected with siGal-8 or control siRNA [mock], reseeded after 24 h and images of 5–6 randomly chosen areas were recorded for three days. The bar graph shows the evaluation of the doubling time as mean ± SEM (* *p* ≤ 0.05, *n* = 4). The western blot on the right shows the Galectin-8 amount 48 h after transfection.

Wound healing and proliferation experiments were performed after the silencing of Galectin-8 expression to elucidate whether Galectin-8 contributes to the development of a malignant phenotype by affecting cell migration or cell proliferation. Figure 4A demonstrates that down-regulation of Galectin-8 significantly inhibited the migration of all PANC-1 cell clones. Normalization of the velocity to the mock transfected control cells (set to 100%) disclosed a migration rate of 79.3 ± 2.73% for siGal-8-treated EGFP-K-Ras-4.1, 67.3 ± 4.68% for EGFP-K-Ras-4.4, 69.88 ± 5.42% for PANC-1 cells, and 72.05 ± 8.26% for EGFP-14. The depletion of Galectin-8 in A549 lung carcinoma cells resulted in a similar reduction with 71.06 ± 5.27% as compared to the control. Thus, an approximately 30% inhibition of cell migration was discovered in pancreatic and lung carcinoma cells. Moreover, Galectin-8 depletion also reduced cell proliferation. The determination of the cell number revealed that the doubling time increased from 26.83 ± 3.34 h to 33.77 ± 4.11 h for EGFP-K-Ras-4.1 cells, 21.49 ± 2.19 h to 32.78 ± 4.05 h for parental PANC-1 cells, and 20.46 ± 3.17 h to 33.76 ± 3.86 h for EGFP-14 cells (Figure 4B, bar graph). Again, Galectin-8 was markedly downregulated, as demonstrated by the western blot in Figure 4B. 

### 2.4. The N-CRD of Galectin-8 Interacts with Farnesylated K-Ras

**Figure 5:**
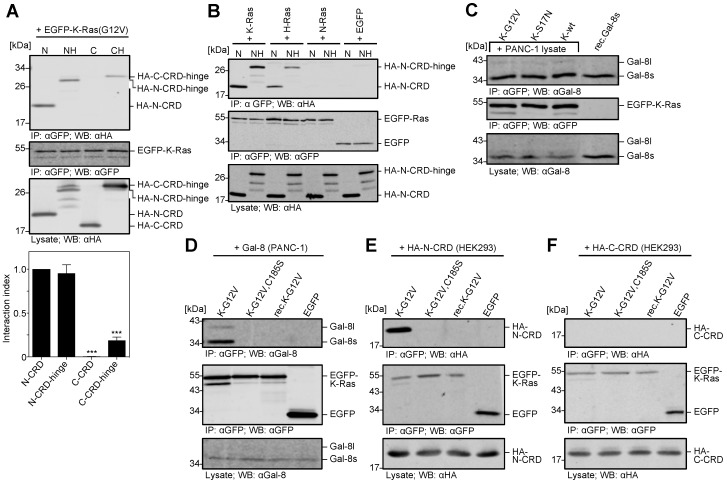
Identification of the interacting domains in K-Ras and Galectin-8. (**A**) Identification of the Galectin-8 carbohydrate recognition domain (CRD) interacting with K-Ras. HEK293 cells were transiently transfected with plasmids encoding for EGFP-K-Ras (G12V) or HA-tagged N-CRD [N], N-CRD-hinge [NH], C-CRD [C], or C-CRD-hinge [CH]. Equal amounts of EGFP-K-Ras were immunoprecipitated and incubated CRD containing lysate. Precipitated proteins were analyzed by western blot. The upper panel shows co-precipitated HA-CRD proteins and the middle panel the immunoprecipitated EGFP-K-Ras proteins. In the lower panel, one-tenth of the HA-tagged CRD-containing lysates were analyzed as an input control. The amount of precipitated HA-CRD proteins was quantified, related to precipitated EGFP-K-Ras and normalized to the amount of HA-N-CRD. The graph shows the interaction index as mean ± SD (*** *p* ≤ 0.001, *n* = 3). (**B**) Interaction of Ras isoforms the CRDs. EGFP-tagged K-Ras(G12V) [K-Ras], H-Ras(G12V) [H-Ras], N-Ras(G12V) [N-Ras], EGFP, as well as HA-N-CRD [N] and HA-N-CRD-hinge [NH] were expressed in HEK293 cells and interaction studies were performed as described in (**A**). The upper panel represents the detection of co-precipitated HA-N-CRDs, the middle panel the detection of precipitated EGFP-Ras isoforms, and the lower panel the amount of HA-CRD in one-tenth of the lysates (all *n* = 3). (**C**) Interaction of Galectin-8 with active and inactive K-Ras. EGFP-tagged, constitutively active K-Ras (G12V) [K-G12V], dominant negative K-Ras (S17N) [K-S17N], and wild-type K-Ras [K-wt] expressed in HEK293 cells were immunoprecipitated and incubated with PANC-1 lysate as a source of endogenous Galectin-8. The precipitates were analyzed by western blot. The upper panel shows the co-precipitated Galectin-8 as well as 30 ng rec.Gal-8s as control. The middle panel presents the precipitated EGFP-K-Ras proteins. In the lower panel the input of Galectin-8 was analyzed in one-twentieth of the applied PANC-1 lysates (*n* = 5). (**D**–**F**) Interaction of Galectin-8 with farnesylated K-Ras. Fully modified EGFP-K-Ras(G12V) [K-G12V], non-farnesylated EGFP-K-Ras(G12V,C185S) [K-G12V,C185S] and EGFP, expressed in HEK293 cells, and unmodified EGFP-K-Ras(G12V) [rec.K-G12V] expressed in *E. coli*, was precipitated and incubated with (**D**) 2 mg of PANC-1 cell lysate, (**E**) 0.5 mg HA-N-CRD, or (**F**) 0.5 mg HA-C-CRD containing HEK293 cell lysate. The precipitates were analyzed by western blot. The upper panel of each figure shows co-precipitated (**D**) Galectin-8, (**E**) HA-N-CRD, and (**F**) HA-C-CRD. The middle panels illustrate the amount of precipitated EGFP-tagged K-Ras proteins and the lower panels the amount of Galectin-8 and HA-CRD, respectively, in one-tenth of the lysates used (*n* ≥ 2).

Galectin-8 belongs to the tandem-repeat Galectins with two CRDs. The N- and C-terminal CRDs of Galectin-8 share 35% homology and might both serve as the binding site for K-Ras. Deletion mutants of Galectin-8, comprising each CRD either alone or in conjunction with the hinge region of Galectin-8 long, were expressed as HA-tagged proteins in HEK293 cells. Figure 5A shows the results of binding experiments with EGFP-K-Ras (G12V). Only the N-CRD, but not the C-CRD was co-precipitated with EGFP-K-Ras (G12V). The adding of the 42 aa hinge region did not alter the interaction of the N-CRD with K-Ras, but it slightly increased binding of the C-CRD-hinge (interaction index: 0.188 ± 0.065) (Figure 5A). Densitometric analysis determined the interaction index, relating the amount of precipitated HA-CRD to the amount of precipitated EGFP-K-Ras (G12V) and normalized to HA-N-CRD. Next, we analyzed whether the observed specificity for the Ras isoforms was also evident when using N-CRD/N-CRD hinge proteins. As shown in Figure 5B, N-CRD markedly co-precipitated with EGFP-K-Ras (G12V), less with EGFP-H-Ras (G12V), and no interaction was observed with EGFP-N-Ras (G12V). Therefore, the N-CRD mediates the preferential binding of Galectin-8 to K-Ras. Cellular fractionation experiments after the ectopic expression of HA-N-CRD and HA-C-CRD in PANC-1 cells demonstrated that only the N-CRD was markedly detectable in the particulate, membrane-containing fraction co-localizing with endogenous Ras Immunofluorescence analysis of the localization of HA-N-CRD and HA-C-CRD ectopically expressed in HEK293 cells confirmed these results, as shown in Supplementary Figure S4B.

The bound nucleotide in Ras changes the confirmation of the protein and its properties to interact with effector and regulatory proteins [23,38]. This leads to the question of how the activity of K-Ras influences its binding to Galectin-8. Constitutive active GTP-bound EGFP-K-Ras (G12V), dominant negative GDP-bound EGFP-K-Ras (S17N), and wildtype EGFP-K-Ras as the control, each being expressed in HEK293 cells, were incubated with PANC-1 cell lysate for precipitation of Galectin-8. Ras activity pull-down assays verified the activity of the used K-Ras mutants (data not shown). The co-precipitation experiments (Figure 5C) revealed no difference in the precipitation of Galectin-8; therefore, the bound nucleotide and the activity of K-Ras do not influence the binding of Galectin-8. These results point towards an interaction domain outside of the switch I and switch II region of K-Ras. We predicted that the farnesylation of K-Ras might be crucial for the interaction in view of the fact that we could not detect any binding of Galectin-8 to unmodified K-Ras expressed in *E. coli* in the previous experiments (see Figure 1E). Precipitation experiments with EGFP-K-Ras (G12V) and EGFP-K-Ras (G12V,C185S) expressed in HEK293 cells, as well as unmodified EGFP-K-Ras (G12V) expressed in *E. coli*, were performed, to prove this hypothesis. The mutation of the C-terminal cysteine to serine (C185S) in K-Ras abrogates K-Ras farnesylation, which leads to cytosolic localization. Hancock and coworkers first described this [8] and demonstrated in our group by its localization in the soluble fraction in cellular fractionation experiments. Figure 5D–F illustrate that Galectin-8 from PANC-1 cell lysates and ectopically expressed N-CRD interacted exclusively with the farnesylated EGFP-K-Ras and not with the farnesyl-deficient mutant or the recombinant K-Ras. None of the K-Ras proteins interacted with the C-CRD fragment. These data clearly demonstrates that the farnesyl moiety of K-Ras is crucial for the interaction with Galectin-8 via its N-CRD. 

### 2.5. The Two Lysine Residues in the Farnesylated Domain of K-Ras Promote the Interaction with Galectin-8

**Figure 6:**
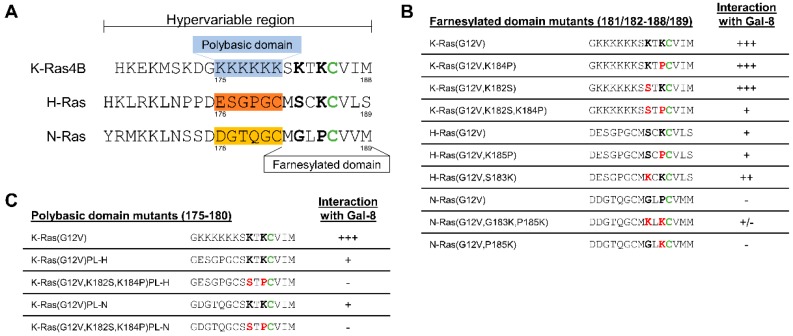
Sequence comparison of the hypervariable region of the Ras isoforms and the generated Ras mutants. (**A**) Sequence comparison of the hypervariable region of K-Ras, H-Ras, and N-Ras. The farnesylated cysteine is marked in green. (**B**) Overview of the Ras mutants in which the two lysines of K-Ras (bold) or corresponding amino acids in H-Ras and N-Ras, respectively, were exchanged. Substitutions are shown in red. (**C**) Overview of the K-Ras mutants in which the polybasic domain was exchanged by the corresponding amino acids of H-Ras or N-Ras. (**B**,**C**) The interaction of the Ras proteins with Galectin-8, based on the experiments shown in Figure 6; Figure 7, is summarized, as follows: [+++] = strong interaction, [+] = weak interaction [−] = no interaction.

**Figure 7:**
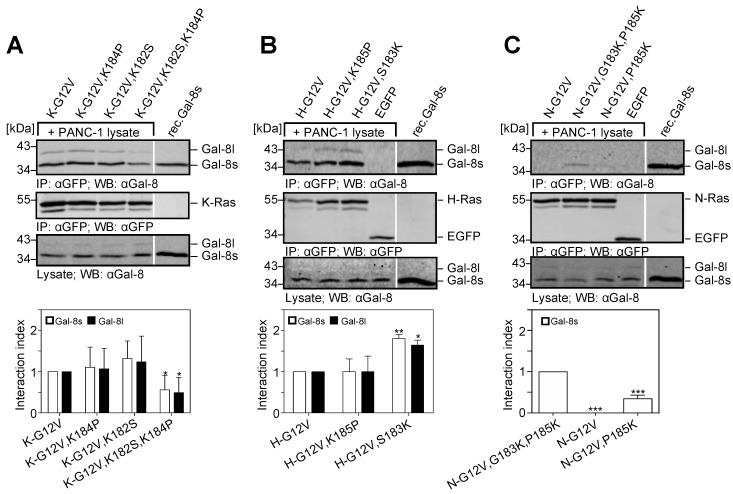
Influence of lysine residues of Ras for the interaction with Galectin-8. Ras mutants were expressed in HEK293 cells: (**A**) K-Ras mutants: [K-G12V], [K-G12V,K184P], [K-G12V,K182S], [K-G12V,K182S,K184P] (**B**) H-Ras mutants: [H-G12V], [H-G12V,K185P], [H-G12V,S183K] (**C**) N-Ras mutants: [N-G12V], [N-G12V,P185K], or [N-G12V,G183K,P185K] (see Figure 6 for a detailed description). Co-precipitation assays were performed with PANC-1 cell lysates and precipitates were analyzed by western blot. 30 ng of rec.Gal-8s was used as a control. The upper panels (**A**–**C**) show co-precipitated Galectin-8 and the middle panels illustrate immunoprecipitated EGFP-Ras proteins. The lower blots show Galectin-8 in 1/25th of the PANC-1 lysates as an input control. The bar graphs document the amount of precipitated Galectin-8 long (black) and short (white), related to the amount of the precipitated EGFP-Ras mutants, and normalized to K-Ras(G12V) (in **A**) or H-Ras(G12V) (in **B**), respectively. The interaction index was calculated with regard to the interaction of Galectin-8 short with N-Ras (G12V,G183,P185K) due to the very low interaction of Galectin-8 with N-Ras(G12V) (in **C**). Each graph shows the mean ± SD of the interaction index (* *p* ≤ 0.05; ** *p* ≤ 0.01; *** *p* ≤ 0.001, *n* = 4–5).

The C-terminal domain of K-Ras is unique with regard to its hexa-lysine polybasic domain adjacent to the farnesylated domain with the characteristic cysteine of the CAAX motif (see Figure 6). Moreover, K-Ras is unique, as it is not palmitoylated within the hypervariable region. These characteristics prompted us to investigate the importance of the lysine residues within the farnesylated domain. Lysine K182 and K184 in K-Ras were mutated to the corresponding serine in H-Ras and the corresponding proline in N-Ras (also see Figure 6 for an overview). Co-precipitation assays, as illustrated in Figure 7A–C, showed that only exchanging one of these lysines did not significantly alter the binding of Galectin-8. However, replacing both lysines significantly reduced the interaction index of Galectin-8 long for K-Ras to 0.49 ± 0.37 (*p* = 0.038) and of Galectin-8 short to 0.56 ± 0.35 (*p* = 0.047) as compared to the wildtype K-Ras form (Figure 7A). Introducing a lysine in position 183 of H-Ras, resulting in two lysine residues next to the farnesylated cysteine, increased the interaction index of both Galectin-8 isoforms for H-Ras by <1.5-fold, as compared to the wildtype form of H-Ras (Figure 7B). However, exchanging K185 in H-Ras to proline did not alter the binding index, which indicated that this lysine alone is not critical for the interaction. N-Ras exhibit no interaction with Galectin-8, but introducing lysine residues in position 183 and 185 resulted in a detectable amount of co-precipitated Galectin-8 short. The insertion of K185 alone produced a weaker interaction with Galectin-8 short.

### 2.6. Removal of the Hexa-Lysine PBD Markedly Abrogates the K-Ras-Galectin-8 Interaction

**Figure 8:**
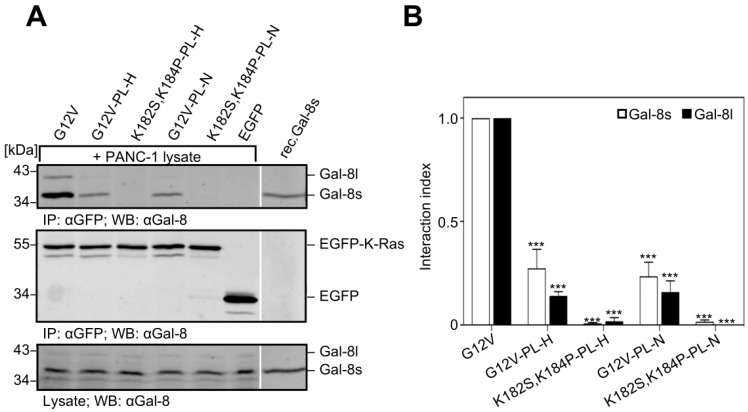
Influence of the polybasic domain of K-Ras for the interaction with Galectin-8. (**A**) The following K-Ras mutants were expressed in HEK293 cells: EGFP-tagged K-Ras(G12V) [G12V], K-Ras(G12V,K182S,K184P) [K182S,K184P] and mutants in which the six lysines KKKKKK175-180 were replaced by the corresponding amino acids ESGPGC of H-Ras [PL-H], or DGTQGC of N-Ras [PL-N] (see Figure 6 for a detailed description). PANC-1 cell lysates were used for the co-precipitation analyses. Western blot analyzed the precipitates. The upper panel shows co-precipitated Galectin-8, the middle panel displays the immunoprecipitated EGFP-K-Ras mutants and the lower panel shows Galectin-8 in one-twentieth of the PANC-1 lysates used. 30 ng of rec.Gal-8s served as a control. (**B**) The bar graph illustrates the quantification of Galectin-8 long (black) and short (white) in relation to the precipitated EGFP-K-Ras mutants expressed as an interaction index. Each graph shows the mean ± SD (*** *p* ≤ 0.001, *n* = 3).

EGFP-K-Ras (G12V) mutants were generated in which the six lysines in the PBD were replaced by the corresponding amino acids of either H-Ras (PL-H) or N-Ras (PL-N) to investigate whether the positively charged PBD (aa 175–180) contributes to the K-Ras-Galectin-8 interaction. Moreover, the lysines in the PBD and the two lysine residues (K182 and K184) in the farnesylated domain were both substituted (see Figure 6C for details). The mutants were expressed as EGFP-fusion proteins in the Galectin-8 negative HEK293 cells, immunoprecipitated, and then incubated with PANC-1 cell lysate to co-precipitate Galectin-8. As exemplified in Figure 8, the substitution of the hexa-lysine stretch in K-Ras caused a significant decrease in the interaction index to 0.27 ± 0.16 for Galectin-8 short and 0.14 ± 0.036 for Galectin-8 long in the case of PL-H, and 0.235 ± 0.12 and 0.16 ± 0.09 in the case the of PL-N when compared to K-Ras (G12V) set to 1.0. When all eight lysine residues in the C-terminus of K-Ras were exchanged, only a faint precipitation of the short isoform of Galectin-8 was evident and the interaction index dropped below 0.02. In summary, the specificity of the interaction of Galectin-8 with K-Ras is indispensably associated with the farnesylation of K-Ras and it is reliant on the existence of multiple lysines within the hypervariable region.

Besides Ras-isoforms, Rho GTPases exhibit a comparable C-terminal domain, although these proteins are geranylgeranylated instead of farnesylated. The comparison of the C-terminal amino acids of K-Ras with Rac, Rho, and Cdc42, as shown in Supplementary Figure S3A, demonstrated that Rac1, its splice variant Rac1b, and Cdc42 also possess two lysine residues in the prenylated domain next to the C-terminal cysteine. Two additional lysine residues are in the adjacent domain in the case of Rac1/Rac1b. RhoA and RhoC exhibit a glycine next to the CAAX motif instead. Precipitation analyses with EGFP-K-Ras (G12V) as a reference revealed that Galectin-8 showed a similar interaction to EGFP-tagged Rac1, Rac1b, and Cdc42. Almost no interaction was evident for EGFP-RhoA or -RhoC. Thus, Rac and Cdc42 may also interact with Galectin-8 in vivo, if they are not occupied by RhoGDIs. 

### 2.7. Structural Model of the Galectin-8 Binding Interface

**Figure 9:**
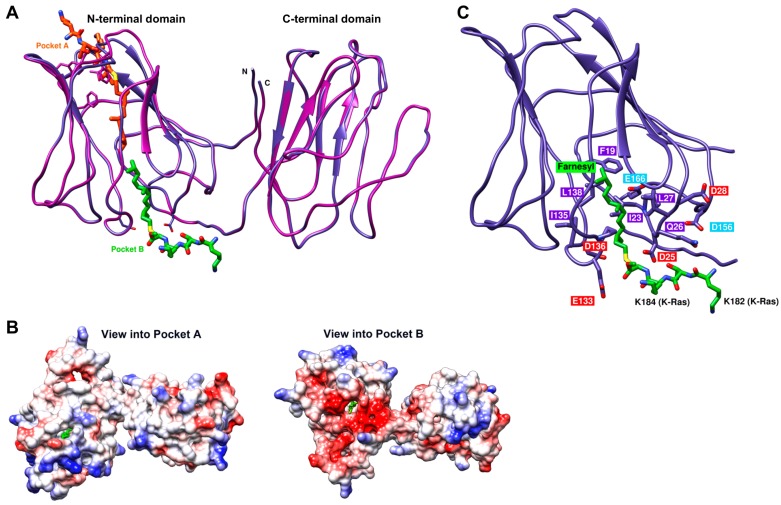
Structural modeling of Galectin-8. (**A**) Structural model of Galectin-8 short, showing the N-terminal, the C-terminal and a putative hinge domain. The two putative farnesyl binding pockets in Galectin-8 are marked by orange (pocket A) and green (pocket B) farnesyl molecules, linked to the C-terminal K-Ras-fragment 182-185 (using the structure of farnesylated and methylated K-Ras4B, RCSB Protein Data Bank: 5tar). (**B**) Comparison of the color-coded surfaces of the two putative farnesyl binding pockets. The electrostatic potential of the molecular surfaces of the cavity of pockets A and B are color-coded [red (-10 kcal/mol) to blue (10 kcal/mol)]. (**C**) Model of the putative farnesyl binding pocket. Pocket residues within a circle of 5 Å of the modeled farnesyl moiety are illustrated. The negatively charged residues are numbered red if they are part of the N-terminal domain of Galectin-8 and cyan if they are part of the hinge domain.

Galectin-1-like N-CRD and a Galectin-2-like C-CRD that are connected by a hinge region (Figure 9A), whose length varies considerably within the human isoforms, comprise Galectin-8. The group of Y. Kloog identified a potential farnesyl binding pocket in Galectin-1 by superimposing the secondary structures of Galectin-1 and RhoGDI [39] using the Cdc42-RhoGDI complex as a model [40]. In RhoGDI, this pocket expands upon binding a geranylgeranyl group. We superimposed the N-terminal domain of Galectin-8 short with Galectin-1 and identified a hydrophobic channel in Galectin-8 that seems to be too narrow to bind a farnesyl chain to localize a putative farnesyl binding pocket in Galectin-8. However, there are two potential binding pockets in the N-terminal domain with the assumption that the binding-pocket expands upon binding the farnesyl group (Figure 9A). Pocket A is similar to the farnesyl binding pocket that was proposed by Rotblat [39] and pocket B is located on the other side of the hydrophobic channel. Consequently, the farnesyl group would enter these potential binding pockets from different sides of the protein. They both proposed hydrophobic binding pockets have to expand upon the binding of the farnesyl molecule, but pocket A requires more structural changes than pocket B. A remarkable difference at the entrance of the pockets is the electrostatic potential. The surface charge of pocket B is negative in contrast to pocket A (Figure 9B). Residues D25, D28, E133, and D136, and residues D156 and E166 from the linker domain form this cluster of negatively charged amino acids in pocket B (Figure 9C). Our binding studies (summarized in Figure 6B) show that the farnesyl moiety of K-Ras and its polybasic region is indispensable for the complex formation with Galectin-8 via the N-CRD; therefore, we suggest that a farnesyl binding pocket in Galectin-8 must exist in conjunction with a negative cluster of amino acids that interact with the positively charged lysine stretch of K-Ras. A cluster of negatively charged residues is only found in the surrounding of the proposed binding pocket B. For these reasons, we assume that, in Galectin-8 pocket B (Figure 9A) and not pocket A, as proposed for Galectin-1, binds the farnesyl moiety of K-Ras. Furthermore, the homologous region in the C-CRD is not negatively charged, which is consistent with our findings that C-CRD does not interact with K-Ras (Figure 5). 

As stated above, the N-CRD resembles Galectin-1, thus we investigated whether EGFP-K-Ras (G12V) from PANC-1 cell lysates also co-precipitated Galectin-1. PANC-1 cells endogenously express Galectin-1, Galectin-3, and Galectin-8, but to considerably different amounts. Densitometric analysis of representative western blots using the amount of recombinant Galectin-1 and Galectin-8 short as references calculated the amount of Galectin-1 and Galectin-8 short in PANC-1 cell lysates. In PANC-1 cell lysates, approximately 0.05 µg/mg lysate Galectin-8 short and 1.7 µg/mg lysate Galectin-1 is present, which is >30 times more Galectin-1 than Galectin-8 short (Supplementary Figure S3B). Western blot analyses of K-Ras (G12V) co-precipitates showed that Galectin-8 and Galectin-1 were both detected in nearly equal amounts in the same precipitate, as referred to the recombinant control protein. But, considering that much less Galectin-8 was present in the sample, calculation of the bound Galectins revealed that approximately 45% of Galectin-8 short, but only 1.3% of Galectin-1 was co-precipitated with K-Ras (G12V) (Supplementary Figure S3, left panel and bar graph in the middle). These results clearly support that K-Ras preferentially interacts with Galectin-8 and not with Galectin-1 when present in the same lysate. These findings support our assumption that the negatively charged region of Galectin-8 (Figure 9), which is missing in Galectin-1, is crucial for complex formation with K-Ras. Exchanging the two lysine residues in position 182 and 184 of K-Ras nearly completely abrogated the binding of Galectin-1, whereas the exchanging of lysine 182 had no effect and the exchanging of lysine 184 downregulated binding to 50% when compared to the wildtype K-Ras (Supplementary Figure S3, right bar graph). This observation implies that the two C-terminal lysine residues are also important for the binding of K-Ras to Galectin-1, probably by hydrophobic interactions mediated by the long hydrophobic lysine side chains. However, K-Ras favors complex formation with Galectin-8. Therefore, we suppose that the hydrophobic effects of the two C-terminal lysine residues are not as crucial for Galectin- 8 as for Galectin-1 binding due to the additional electrostatic interactions within the N-CRD of Galectin-8 with the multiple lysines in K-Ras.

In conclusion, we have demonstrated within this work that Galectin-8 is a new and direct binding partner for K-Ras. The N-CRD specifically mediates the interaction with the farnesylated PBD of K-Ras, thereby modulating K-Ras effector pathways as well as cell proliferation and migration. The current results underscore the potential of Galectin-8 as a new intracellular signaling modulator and pharmacological target in K-Ras-dependent tumors. Inhibiting Galectin-8 and, hence, interfering with K-Ras signaling, might represent a new strategy to block tumor cell proliferation and motility and probably metastatic dissemination.

## 3. Discussion

In the presented study, we have combined the biochemical, structural, and cellular analyses to identify and characterize Galectin-8 as a new direct binding protein for K-Ras, which modulates the signal transduction pathways downstream of Ras and cellular functions involved in tumorgenesis. 

Our precipitation experiments demonstrate that the two isoforms of Galectin-8 directly bind to K-Ras. These two isoforms differ with regard to the expression of exon 11, encoding for 42 amino acids located in the hinge region between the N- and C-terminal CRD. Both isoforms are expressed at the protein level in 19 of 20 analyzed lung and pancreatic carcinoma cell lines (Supplementary Figure S1). This high frequency correlates with the findings of different groups, which showed that Galectin-8 is one of the most widely expressed tandem-repeat galectins in human cells and tumors [41,42]. Most of the studies concentrate on extracellular functions of secreted Galectin-8, which, for example, can either operate as a matrix protein, thereby supporting cell adhesion, or as a soluble ligand, thereby inhibiting cell adhesion by modulating integrin β1 signaling [20,41,42,43]. However, its role in oncogenesis and tumor progression is not well understood, since its expression can be found as being increased or decreased in different tumor types [41,44]. The increase in the Galectin-8 gene (LGALS8) expression correlates with increased human lung cancer progression and metastasis [45]. Unpublished, preliminary studies from our group indicates increased Galectin-8 mRNA expression in over 50% of the analysed sample from pancreatic carcinoma patients as compared to normal tissue. However, Galectin-8 seems to be down-regulated during the progression of pancreatic cancer, as detected in another study using immunohistochemistry [46]. Therefore, the available data point towards an organ and tumor-specific function of Galectin-8.

Galectins shuttle between the nucleus, cytoplasm, and extracellular compartment. Targeting to the membrane is especially evident for Galectin-1 and -3 when interacting with active H-Ras or K-Ras, respectively [21]. Cell lysate fractionation analyses, as shown for PANC-1 cells in this study (Figure 2) and several other carcinoma cell lines, revealed that Galectin-8 is localized in the cytosolic and membrane-containing fraction and it co-localizes with K-Ras at the plasma membrane. Essential studies by Hancock and colleagues have established that farnesylated K-Ras is mainly localized in non-raft microdomains through the electrostatic interaction of its PBD with the plasma membrane and it is recruited into nanoclusters after GTP loading [7,14,47]. A complex interaction of the farnesyl group and the PBD with acidic phospholipids, such as phosphatidylserine, mediates nanocluster formation. Up to approximately 40% of the K-Ras molecules are arranged in nanoclusters of ~6 proteins, whereas the remaining molecules are localized as monomers or dimers in the plasma membrane [14,48]. K-Ras nanocluster formation is modulated by PKC-mediated phosphorylation of S181, which changes the charge of the PBD of K-Ras [10], as well as by Galectin-3 [23]. Galectin-3 binds to membrane lipids, including phosphatidylserine, and sequesters the farnesyl moiety of K-Ras within in its hydrophobic pocket. This stabilizes K-Ras.GTP nanoclusters which act as a signaling hub to recruit downstream effectors, such as Raf-1 and PI3K [16,23]. The data presented here demonstrate that the interaction of K-Ras and Galectin-8 relies heavily on both the farnesyl moiety and the lysine residues within the HVR. K-Ras lacking farnesylation did not interact with Galectin-8, pointing towards a farnesyl binding pocket that is similar to the ones already discovered in Galectin-1, Galectin-3 [23,39,49], and PDEδ [11]. Sequence alignments and structural comparisons showed that Galectin-8 exhibits a Galectin-2-like C-CRD and a Galectin-1-like N-CRD (Figure 9), with the latter showing two potential farnesyl binding pockets. We propose that the N-CRD mediates binding to K-Ras via the hydrophobic pocket B, since the N-CRD alone or in conjunction with the hinge region strongly interacted with farnesylated K-Ras (Figure 5). This pocket is surrounded by a cluster of negatively charged amino acid residues, which could interact with the positively charged lysine residues within the HVR of K-Ras, as depicted in Figure 9C. This negative cluster is formed by residues D25, D28, E133, and D136, which are expressed in all Galectin-8 variants, and supported by residue D156 and E166 from the hinge domain. There are x-ray structures of the N- and the C-terminal subunits of Galectin-8, but none for the hinge region, which might lead to certain inaccuracies in the proposed folding of the hinge domain. The superimposition of the N- and C-CRD of Galectin-8 shows that both domains share a similar fold. However, no negative cluster is found in the equivalent potential cavity region of the C-CRD. Thus, we assume that Galectin-8 binds only one K-Ras molecule via the N-CRD. Furthermore, a comparison of our model with other Galectin-8 structures (RCSB Protein Data Bank: 4han, 4fqz) and with those of Galectin-3 (4lbo) and Galectin-1 (3t2t) revealed a very similar overall fold but the described negative cluster is only present in Galectin-8. Our proposed model is in accordance with the experimental data, which showed that, besides the farnesylation, the two lysines adjacent to the farnesylated cysteine, as well as the six lysines in the PBD of K-Ras, are crucial for the interaction (as summarized in Figure 6B,C). Moreover, introducing a second lysine into the farnesylated domain of H-Ras enhances the weak binding to Galectin-8, which points towards a supportive electrostatic interaction with the negatively charged residues in the binding cavity of Galectin-8. However, we cannot entirely exclude that H-Ras uses pocket A on the opposite side of the molecule, instead of pocket B. 

Until now, Galectin-3 has been discussed as a chaperone-like protein for K-Ras.GTP leading to nanoclustering and subsequent downstream signaling. The K-Ras-Galectin-3 complexes were originally discovered in NIH/3T3 cells [24], a cell line that hardly expresses Galectin-8. In our precipitation analyses with K-Ras, we could hardly detect co-precipitated Galectin-3. Thus, Galectin-8 might either compete with Galectin-3 for binding to K-Ras or it might interact with a subset of K-Ras molecules not targeted by Galectin-3. The finding supports the latter hypothesis that Galectin-8 interacts with GTP-, as well as GDP-bound K-Ras (Figure 5C), whereas Galectin-3 only interacts with K-Ras.GTP trapped in nanoclusters [23]. Galectin-8 might target these molecules, given that 50–60% of the K-Ras molecules are segregated as monomers in the membrane, possibly in a latent signaling state. As demonstrated here, deletion of Galectin-8 led to an upregulation of the total and membrane-bound EGFP-K-Ras (G12V) protein content (Figure 2C,E). This might be the result of the stabilization of GTP-bound EGFP-K-Ras (G12V) by binding now to Galectin-3 and by reduced cycling and/or proteasome-mediated degradation of K-Ras. The trafficking, recycling, and degradation of K-Ras is controversially discussed within the literature. K-Ras, phosphorylated via PKC on S181, traffics from the plasma membrane to the ER and Golgi [10], and it can be endocytosed and targeted to early and late endosomes, as well as lysosomes [35]. The inhibition of lysosomal degradation extended the half-life of K-Ras and caused sustained ERK activation after EGF stimulation [31,35]. The group of J. Hancock reported a mislocalization of K-Ras by using Fendiline and Staurosporine, thereby inhibiting the interaction with the plasma membrane and directing K-Ras to the cytosol, endosomes, mitochondria, Golgi, and the ER [50,51]. Galectin-8 might serve as an escort protein for K-Ras endosomal trafficking, since we observed Galectin-8 in the membrane of Rab-7 containing endosomes (our unpublished results). Polyubiquitination also controls K-Ras protein stability [36]. Our data, showing that the proteasome inhibitor MG132 increases the steady-state level of EGFP-K-Ras (G12V) similar to the effect seen after Galectin-8 depletion (Figure 3B), might also offer the possibility that Galectin-8 is involved in proteasome-mediated K-Ras degradation. The enhanced EGFP-K-Ras (G12V) content in the analyzed PANC-1 cell clones (Figure 2 and Figure 3) after Galectin-8 depletion resulted in enhanced ERK activation, which enhanced K-Ras (G12V) nanocluster formation could mediate by binding to Galectin-3 and it recruitment to the plasma membrane. 

K-Ras traffics via a still uncharacterized Golgi-independent pathway to the plasma membrane. Thus, Galectin-8 might primarily act as a GDI-like solubilizing factor by shielding the farnesyl group and assisting its intracellular trafficking to or from membranes, as described for PDEδ [11,52]. PDEδ controls the accurate localization of K-Ras at the plasma membrane by enhancing the solubilization and transmembrane exchange of K-Ras from endomembranes to the plasma membrane. The depletion of PDEδ results in reduced plasma membrane localization and ERK signaling in MDCK cells [52]. Our data show that Galectin-8 depletion upregulates EGFP-K-Ras (G12V) protein content in the particulate and slightly in the soluble fraction (Figure 2E). These data might point towards opposite functions of Galectin-8 and PDEδ in mediating plasma membrane to endomembrane trafficking and the localization of K-Ras. Further work is needed for solving the molecular mechanism how Galectin-8 acts as a regulator for the dynamic equilibrium of membrane-bound and cytosolic K-Ras and its protein stability.

It is well documented that human pancreatic and lung cancer with oncogenic mutation in the K-Ras allele is highly aggressive and is associated with poor prognoses. Fig. 4 documents a function for Galectin-8 in controlling tumor-relevant functions, such as cell proliferation and migration. Cell migration was reduced by approx. 30% and the doubling time increased by approx. 25% to 65%, depending on the cell clone, leading to a reduction in cell proliferation, upon the depletion of Galectin-8. The moderate reduction of cell proliferation and migration might be due to the considerable amount of Galectin-8 that is still present in the cells after siRA treatment. A CRISPR/Cas-mediated inhibition of Galectin-8 would be required to further validate Galectin-8 as a pharmacological target.

Taken together, we have identified Galectin-8 as a new and direct binding partner for K-Ras and have characterized the respective binding interfaces in Galectin-8 and K-Ras. The current results underscore the potential of Galectin-8 as a new intracellular signaling molecule and possible pharmacological target in K-Ras-dependent tumors since Galectin-8 modulates K-Ras-induced signaling, proliferation, and migration. Inhibiting Galectin-8 itself or its binding to K-Ras and, thus, impeding K-Ras signaling might represent a new strategy to block tumor cell proliferation and motility and probably metastatic dissemination.

## 4. Materials and Methods

### 4.1. Antibodies, Inhibitors and Recombinant Proteins

Antibodies, inhibitors, and recombinant proteins were obtained from standard suppliers and are listed in Supplementary Materials.

### 4.2. Plasmids, Primers and Buffers

Expression plasmids for the ectopic expression of EGFP-K-Ras (G12V), -H-Ras (G12V), -N-Ras (G12V), -Rac1, -Rac1b, -RhoA, -RhoC, and -Cdc42 were produced by ligating the coding sequence in frame into pEGFP-C vectors (Clontech, Heidelberg, Germany). All of the plasmids and buffers are described in detail in Supplementary Materials File. 

### 4.3. Cell Lines and Culture Conditions

The human pancreatic carcinoma cell line PANC-1, the human embryonic kidney cell line HEK293, the human lung carcinoma cell lines A549 (all from the American Type Culture Collection, USA), and Colo699 (DSMZ, Braunschweig, Germany) were maintained in DMEM that was supplemented with 10% fetal calf serum (FCS) (Capricorn, Ebsdorfergrund, Germany), 2 mM L-glutamine, 0.1 mM MEM non-essential amino acids solution (ThermoFisher Scientific, Langenselbold, Germany) at 37 °C in a humidified atmosphere with 10% CO_2_ and routinely tested for mycoplasma contamination. The cell clones stably transfected with pEGFP-C3 or pEGFP-C3/K-Ras (G12V) were generated and cultured, as described in [34]. Serum-starvation was performed overnight in DMEM without supplements. *Spodoptera frugiperda* 9 (*Sf*9) insect cells (ThermoFisher Scientific, Langenselbold, Germany) were cultivated in Grace’s Insect Medium (Sigma-Aldrich, Taufkirchen, Germany) that was supplemented with 10% FCS, 10 µg/mL Gentamicin, 2 mM L-glutamine at 27 °C. Recombinant baculoviruses expressing K-Ras or Galectin-8 variants were generated by transfecting *Sf*9 cells with pVL1393 encoding HA-K-Ras(G12V), HA-Gal-8 long, His-K-Ras(G12V), or His-Gal-8 long and a modified baculovirus DNA (Baculogold, Pharmingen; ProGreen^TM^ or ProEasy^TM^ AB Vector, USA), according to the manufacturer protocol. High-titer stocks of baculovirus-containing cell culture supernatants were obtained by 3–5 cycles of amplification in Sf9 cells and they were used for protein production in infected *Trichoplusia ni* High FiveTM (H5) (BTI-Tn-5B1-4; ThermoFisher Scientific, Langenselbold, Germany), as described in [53].

PANC-1 cells (3 × 10^6^ cells / 10 cm dish) were transfected with 8 μg of plasmid DNA and DMRIE-C reagent (Invitrogen, Groningen, The Netherlands), as described in detail in (34). HEK293 cells (7 × 10^6^ cells / 10 cm dish) were transfected while using 27.5 µL polyethylenimine (PEI, 1 mg/mL, Sigma-Aldrich, Taufkirchen, Germany) and 8 µg of plasmid. Protein expression was enabled for 48 h at 37 °C and 10% CO_2_ to ensure correct post-translational modification processes. The cells were lysed in an appropriate lysis buffer.

For siRNA transfection, PANC-1 cells (8 × 10^5^ cells/well, six-well plate), 7.5 µL of Lipofectamine2000 (ThermoFisher Scientific, Langenselbold, Germany), and 50–70 nM siRNA (SMARTpool: ON-TARGETplus LGALS8 siRNA, or ON-TARGETplus Non-targeting Pool, Dharmacon / Horizon Discovery, Cambridge, UK), or 5 µl of DMRIE-C (ThermoFisher Scientific, Langenselbold, Germany) and 20–80 nM siRNA in OptiMEM, were used. 7.5 µL Dharmafect 1 (Dharmacon/Horizon Discovery, Cambridge, UK) and 25 nM siRNA in DMEM without supplements transfected Colo699 cells (1 × 10^6^ cells/well). The medium was replaced by FCS-containing medium after 6 h and the cells were lysed 72 h after transfection. In indicated experiments, the cells were additionally treated with 5 µM MG132 (Calbiochem, Darmstadt, Germany) for 24 or 48 h.

### 4.4. Protein Lysate Preparations

Cell lysates were prepared in Gold-Lysis buffer or for phosphorylation analyses in IP buffer that was supplemented with proteinase and phosphatase inhibitors, being homogenized by forcing the suspension through a 0.45 × 25 mm needle attached to a syringe 5–10 times, and cleared via centrifugation at 16100× *g* for 15 min. at 4 °C. Western blotting analyzed 50 µg of protein lysates.

### 4.5. Subcellular Fractionation

Soluble (S100) (cytosolic) and particulate (P100) (membranous) fractions were prepared by centrifuging cell lysates at 100,000× *g* in HEPES buffer essentially as described in [34].

### 4.6. Purification of His-Tagged Proteins Using Ni-NTA Agarose Resin

Baculovirus-infected H5 insect cells were maintained for 96 h at 27 °C in Ex-Cell 405 medium (Sigma-Aldrich, Germany) and then lysed in His buffer. Ni-NTA Agarose Resin (Serva, Heidelberg, Germany) was equilibrated with His buffer without 0.5% (m/V) sodium deoxycholate and incubated with cell lysate for 1 h at 4 °C by end over end rotation. The resin was washed 4 x with Ni-NTA washing buffer containing 20 mM imidazole for 15 min. at 4 °C by end-over-end rotation. His-tagged proteins were eluted 3× while using 100 µL of elution buffer [Ni-NTA washing buffer containing 250 mM imidazole] by end-over-end rotation for 10 min. at room temperature. 

### 4.7. Identification of Ras-Interacting Proteins

HA-K-Ras (G12V), HA-H-Ras (G12V), and HA-N-Ras (G12V) were expressed in baculovirus-infected *Sf*9 insect cells and then purified from the particulate (P100) membrane-containing fraction. 30 mg of crude P100 extract was incubated with anti-HA (12CA5) antibody (Roche, Mannheim, Germany) that was covalently linked to Protein A Sepharose^®^ Cl-4B (GE Healthcare, Freiburg, Germany) in HA-binding buffer for 2 h at 4 °C with constant rotation. The matrix was washed 4× with binding buffer containing 400 mM NaCl. Bound proteins were eluted in four steps (750 µL each) with binding buffer containing 1 M NaCl and then dialyzed overnight in binding buffer with 50 mM NaCl. Purified HA-tagged Ras proteins (500 ng) were immunoprecipitated for 2 h at 4 °C while using µMACS HA Isolation Kit and 50 µL of HA-MicroBeads (Miltenyi Biotec, Bergisch Gladbach, Germany). Thereafter, 10 mg of PANC-1 cell lysate in Gold-Lysis buffer, precleared with 10 µg mouse IgG2a (DakoCytomation, Waldbronn, Germany) and Protein-G Agarose, was added for 2 h at 4 °C under constant rotation. The protein complexes were purified on µColumns (Miltenyi Biotec, Bergisch Gladbach, Germany) and washed 1× with MACS washing buffer 1, 4× with Gold-Lysis buffer containing 250 mM NaCl, and 2× with washing buffer 2 [20 mM Tris HCl pH 7.5]. The proteins were eluted in 40 µL of elution buffer that was preheated to 95 °C. Eluted proteins were separated on 10% SDS-polyacrylamide gels and silver-stained. Selected bands were excised for ESI-MS⁄MS mass spectrometry and then analyzed as described in [54]. Proteins were identified by correlating the ESI-MS/MS spectra with the NCBI nr protein sequence data base while using MOWSE algorithm and MASCOT software (www.matrixscience.com, accessed in July and September, 2004) with the Mascot score as a measurement for reliability.

### 4.8. Protein Interaction and Co-Immunoprecipitation Assays

GTPases were stably or transiently expressed in PANC-1 or HEK293 cells to characterize the interaction of Galectins with EGFP-Ras and EGFP-Rho GTPases. PANC-1 lysates were used as a source of endogenous Galectins. Galectin-8 variants were ectopically expressed in HEK293 cells, and lysates were prepared in Gold-Lysis buffer. Interaction assays were performed while using the µMACS GFP Isolation Kit (Miltenyi Biotec, Bergisch Gladbach, Germany). The protein lysates with equal amounts of EGFP-Ras proteins, quantified before by western blot analysis, were incubated with anti-GFP µMacs MicroBeads in Gold-Lysis buffer for 30 min. at 4 °C. PANC-1 lysate (2 mg) as a source for endogenous galectins or lysate containing ectopically expressed Galectin variants was added for 2 h at 4 °C. The interaction mixture was applied to equilibrated µColumn and washed 4 x with MACS washing buffer 1 and 2× with washing buffer 2 [20 mM Tris HCl pH 7.5]. Immunoprecipitated proteins were eluted with preheated elution buffer and then analyzed via western blot procedure. Rec. Galectin-8 short, -3, and -1 (R&D Systems, Wiesbaden, Germany) were applied as controls.

Endogenous or stably expressed proteins were co-immunoprecipitated while using appropriate µMacs isolation kits. For co-immunoprecipitation of endogenous proteins, the cells were lysed in IP buffer, 1–5 mg of protein lysate was incubated with 2 µg of the appropriate antibody and 25 µL µMacs Protein A or Protein G MicroBeads. The interaction mixture was handled as described above. For co-immunoprecipitation analyses with EGFP proteins, cells lysates were prepared in IP buffer, incubated with 25 µL anti-GFP µMacs MicroBeads, and then purified as described above. 

Immunodot blotting was performed for analyzing the direct protein-protein interaction of K-Ras 4B and Galectin-8. His-K-Ras (G12V) or His-Gal-8l, produced in insect cells and purified while using Ni-NTA agarose, was spotted onto a nitrocellulose membrane in increasing concentrations and the membrane was blocked with 3% BSA/TBS-T. Purified recombinant proteins produced in *E. coli* were separately spotted on the same membrane to evaluate the amount of bound protein and antibody specificity. Spotted His-K-Ras(G12V) was incubated overnight with 1 µg purified rec. Gal-8s solved in IP buffer and spotted His-Gal-8l with 1 µg purified HA-K-Ras (G12V) produced in insect cells. The indicated primary antibody and HRP-conjugated secondary antibody incubated the nitrocellulose membrane. Bound proteins were detected by ECL. 

### 4.9. Ras Activity Assays

The GTP-bound forms of K-Ras, H-Ras, and N-Ras were recovered from cell lysates by affinity precipitation while using a GST fusion protein of the Ras-binding domain (RBD) of Raf-1 as an activation-specific probe for Ras-GTP essentially as described in [34].

### 4.10. Migration and Proliferation Assays

For wound healing assays, PANC-1 cells were seeded into six-well plates (8 × 10^5^ cells/well), transfected with 50 nM siRNA, and cultured for 24 h, as described above. After treating the cells with mitomycin-C (10 µg/mL) for 2 h, three parallel scratches were made into the confluent monolayer while using a pipette tip. The washed cells were incubated in DMEM without supplements. Images of six randomly chosen areas at the scratches were taken every 3 h for 48 h using EVOS FL Auto 2 Cell Imaging System (ThermoFisher Scientific, Germany). Stacks of all images made for one point within the scratch were generated using Fiji (Version 1.52i) and analyzed by Scratch Assay Analyzer Plugin (MiToBo, Universität Halle) [55]. Cell migration was calculated in [µm/h]. The velocities were normalized to the control of each cell line. For proliferation assays, the cells were transfected with 33 nM siRNA. 24 h later, cells (5 × 10^4^) were seeded into 12-well plates and then incubated for 12 h to allow for cell attachment. Images of 5–6 randomly chosen areas/well were taken in 12 h interval, using EVOS FL Auto 2 Cell Imaging System. The cells were counted while using Fiji software at time points 0 h (≙ 36 h after transfection) at least 48 h (≙ 84 h after transfection). The doubling time was calculated for each experimental setup. 

### 4.11. Structural Modeling

The structures of two human Galectin-8 isoforms [Gal-8s: 317 aa, Gal-8l: 359 aa] were modeled according to the crystal structures from human Galectin-8 (4han: 293AS [56] and 4fqz: 291 aa [57]) while using the SWISS-MODEL automated comparative protein modeling server [58]. In the modeled range, the sequence identity of Gal-8s and Gal-8l to 4han and to 4fqz is at least 99.0%. Our model was superimposed with other structures via secondary structure matching (SSM) [59]. The farnesyl moiety, which was linked to the C-terminal end of K-Ras (taken from 5tar [60]), was manually modeled into the binding pocket with the interactive graphics program Coot [61], subsequently geometry minimization of the Galectin-8-geranylgeranyl complex was performed with PHENIX [62]. 

### 4.12. Miscellaneous

The bicinchoninic acid (BCA) assay determined the protein concentration (Pierce, Sankt Augustin, Germany). Ponceau S staining controlled the equal loading and transfer of proteins (Sigma-Aldrich, Taufkirchen, Germany). Immunoreactive bands were detected by using the appropriate antibodies and either ECL Western Blotting Detection System (GE Healthcare, Munich, Germany) and Fusion Imaging System and Software (Vilbert Lourmat, Eberhardzell, Germany) or Odyssey Infrared Imaging System and Software (LI-COR Biosciences, Bad Homburg vor der Höhe, Germany). All of the experiments were repeated as indicated, and similar results and identical trends were obtained each time. Data from representative experiments are shown. Western blot quantification was performed while using Image Studio Lite Software (Li-COR Biociences, Bad Homburg vor der Höhe, Germany). All of the values are presented as mean ± SD or SEM and statistical significance was calculated using one sample t test (Prism 7, GraphPad Software Inc., San Diego, CA, USA). A value of *p* ≤ 0.05 was considered to be significant.

## 5. Conclusions

In summary, our study uncovered Galectin-8 as a novel intracellular binding partner for K-Ras4B, which modifies K-Ras stability, ERK signaling, and migration and proliferation in pancreatic and lung carcinoma cells. The biochemical and structural characterization of the K-Ras-Galectin-8 interacting domains revealed that the binding is mediated via the farnesyl moiety of K-Ras and is highly supported by the polybasic stretch of lysine residues in the C-terminus of K-Ras. With regard to Galectin-8, the interaction is facilitated by the N-CRD and not the C-CRD. The N-CRD harbors a pocket for incorporating the farnesyl moiety of K-Ras, and a cluster of negatively charged amino acids, which presumably interacts with the positively charged lysines of K-Ras. The inhibition of Galectin-8 and, hence, interference with K-Ras signaling, may represent a new strategy for blocking tumor metastasis, since Galectin-8 is abundantly expressed in carcinoma.

## Supplementary Materials

The following are available online at https://www.mdpi.com/2072-6694/12/1/30/s1, Figure S1: Expression of Galectin-1, -3 and -8 in pancreatic and lung carcinoma cell lines. Figure S2: Interaction of Galectin isoforms with EGFP-Ras. Figure S3: Interaction of Galectin with GTPases. Figure S4: Immunofluorescence analysis of Galectin-8 and its CRDs.
