# Supplementary material for: Galectin-8 binds to the Farnesylated C-terminus of K-Ras4B and Modifies Ras/ERK Signaling and Migration in Pancreatic and Lung Carcinoma Cells
Source: Cancers (Basel). 2019 Dec 20;12(1):30. doi: 10.3390/cancers12010030 (PMC7017085; doi:10.3390/cancers12010030)
Supplement: Supplementary file 1 [file cancers-12-00030-s001.zip › cancers-672634-Suppl-final/cancers-672634-suppl-final.docx]

Article

Galectin-8 binds to the Farnesylated C-terminus of K-Ras4B and Modifies Ras/ERK Signaling and Migration in Pancreatic and Lung Carcinoma Cells

Christopher Meinohl ^1^, Sarah J. Barnard ^1^, Karin Fritz-Wolf ^2,3^, Monika Unger ^4^, Andreea Porr ^5^, Marisa Heipel ^1^, Stefanie Wirth ^1^, Johannes Madlung ^6^, Alfred Nordheim ^6,7^, Andre Menke ^8^, Katja Becker ^3^, and Klaudia Giehl ^1,^*


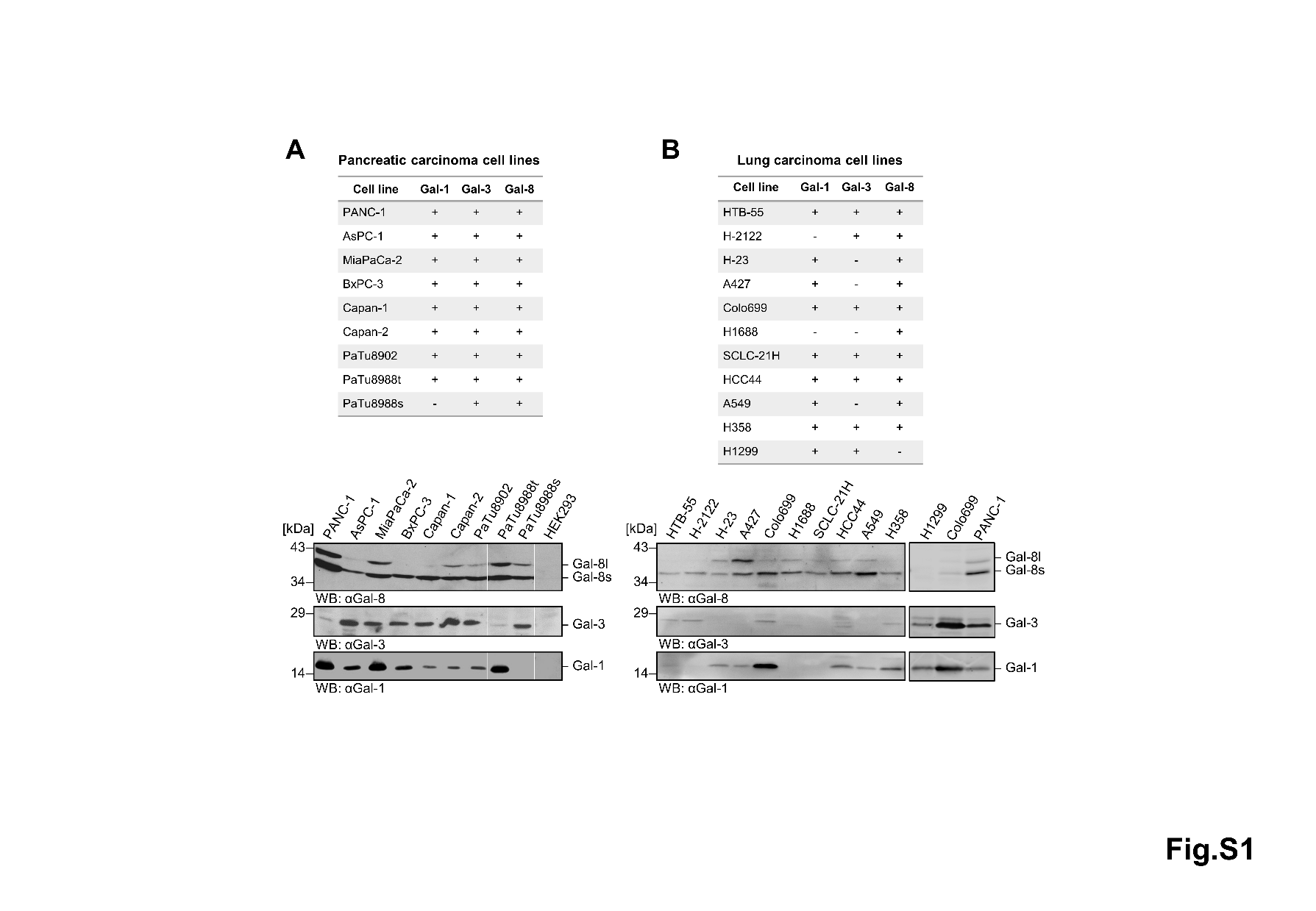


**Figure S1:** Expression of Galectin-1, -3, and -8 in pancreatic and lung carcinoma cell lines. RIPA cell lysates of 9 pancreatic carcinoma cell lines (**A**) and 11 lung carcinoma cell lines (**B**) and HEK293 kidney cells were analyzed by SDS-PAGE and western blot regarding their expression of Galectin 1 [Gal-1], Galectin-3 [Gal-3], and Galectin-8 [Gal-8]. All pancreatic carcinoma cells expressed the three galectins except PaTu8988s lacking Galectin-1. Galectin-8 was expressed in all lung carcinoma cell lines analyzed except H1299, but only half of these cell lines expressed all three galectins. Moreover, Galectin-8 short expression is more pronounced than Galectin-8 long in all cell lines except A427. HEK293 kidney cells do not express detectable amounts of these Galectins.


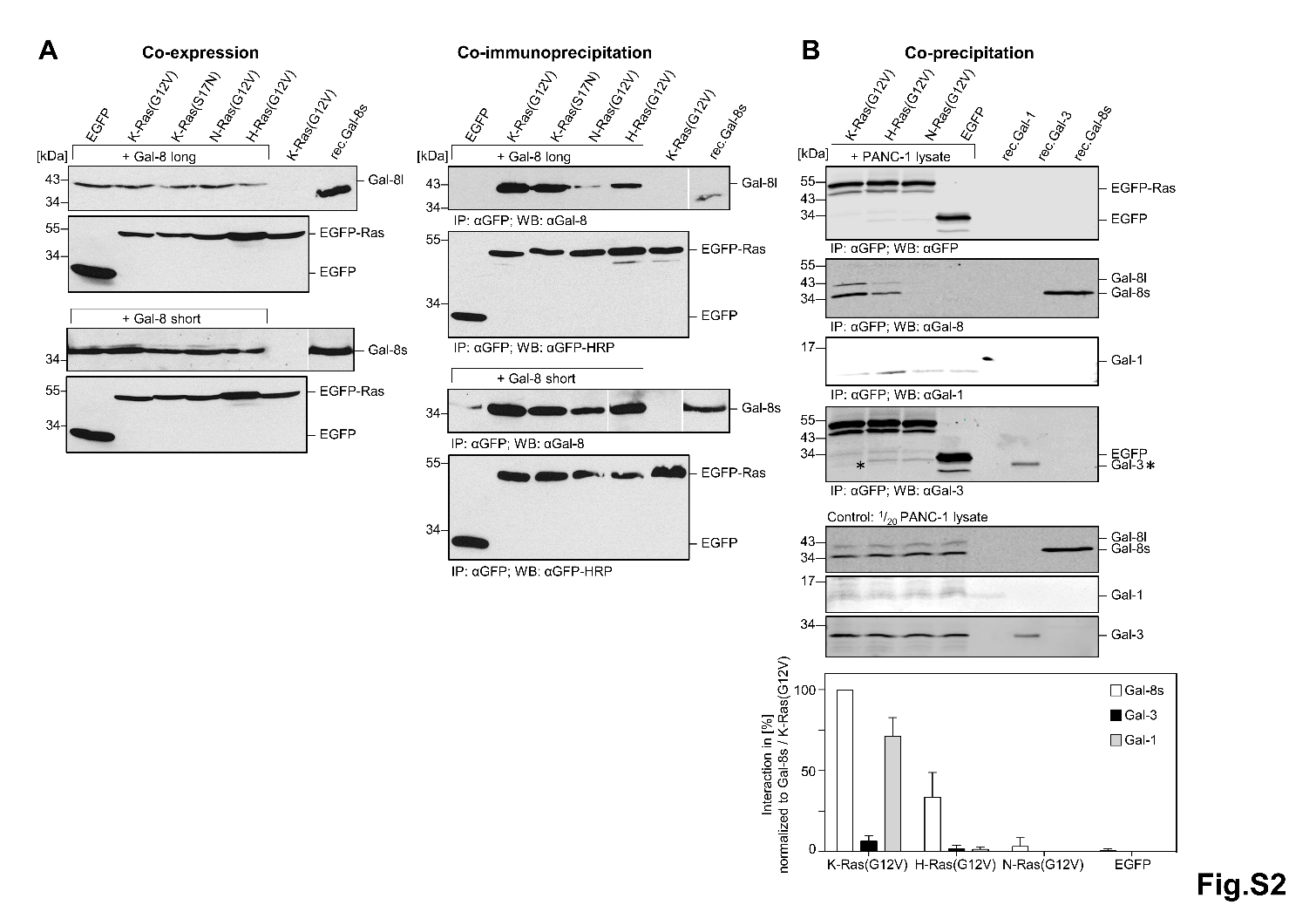


**Figure S2:** Interaction of Galectin isoforms with EGFP-Ras. (**A**) HEK293 cells were co-transfected with 5 μg of plasmids encoding for EGFP-tagged K-Ras(G12V), K-Ras(S17N), H- Ras(G12V), N-Ras(G12V), EGFP, and 10 μg of plasmids for Gal-8l or Gal-8s. Cells were lysed after 48 h. (Left panel) Co-expression of Gal-8l and Gal-8s together with EGFP-tagged Ras was analyzed by western blot using 50 μg of total cell lysate and anti-GFP-HRP antibody, respectively. Recombinant Gal-8s was used as a control. (Right panel) Equal amounts of EGFP-Ras or proteins were immunoprecipitated from each protein lysate using anti-GFP μMacs beads. The EGFP-/Ras-precipitates were analyzed by western blot. EGFP, EGFP-Ras, and Galectin-8 were detected using anti-GFP-HRP or anti- Galectin-8 antibody, respectively. Co- precipitated Gal-8l and Gal-8s is shown in the corresponding upper panel and precipitated EGFP or EGFP-Ras in the lower panel. Ectopically expressed Gal-8l and Gal-8s were markedly co-precipitated with co-expressed EGFP-K-Ras(G12V) and EGFP-K-Ras(S17N), less with EGFP-H- Ras(G12V), and to a small degree with EGFP-N-Ras(G12V), again demonstrating the preference of Galectin-8 to interact with K-Ras. (**B**) To investigate whether endogenous Galectin-1 or Galectin-3 also co-precipitated with EGFP-Ras isoforms, HEK293 cells were transfected with plasmids encoding for EGFP-tagged K-Ras(G12V), H-Ras(G12V), N-Ras(G12V), or EGFP and lysed after 48 h. Equal amounts of EGFP proteins were precipitated using anti-GFP μMacs beads before 2 mg of PANC-1 lysate was added for 2 h. The precipitates were analyzed as described in (A). For each Galectin, a recombinant Galectin of known quantity (Gal-1: 40 ng; Gal-3: 10 ng; Gal-8s: 30 ng) was used to calculate the amount of each Galectin precipitated by densitometric comparison. The bar graph shows the calculations as mean ± SD of at least two independent experiments normalized to the interaction of Gal-8s to EGFP-K-Ras(G12V), set to 100 %. Galectin-8 short predominantly interacts with EGFP-K-Ras(G12V), markedly less with H-Ras(G12V), and hardly at all with N- Ras(G12V). Endogenous Galectin-1 was only precipitated with EGFP-K-Ras(G12V) but to a smaller extent than endogenous Galectin-8. The very faint band of Galectin-3 precipitate with EGFP-K-Ras(G12V) is marked (*). All other bands represent unspecific bands not related to Galectin-3.


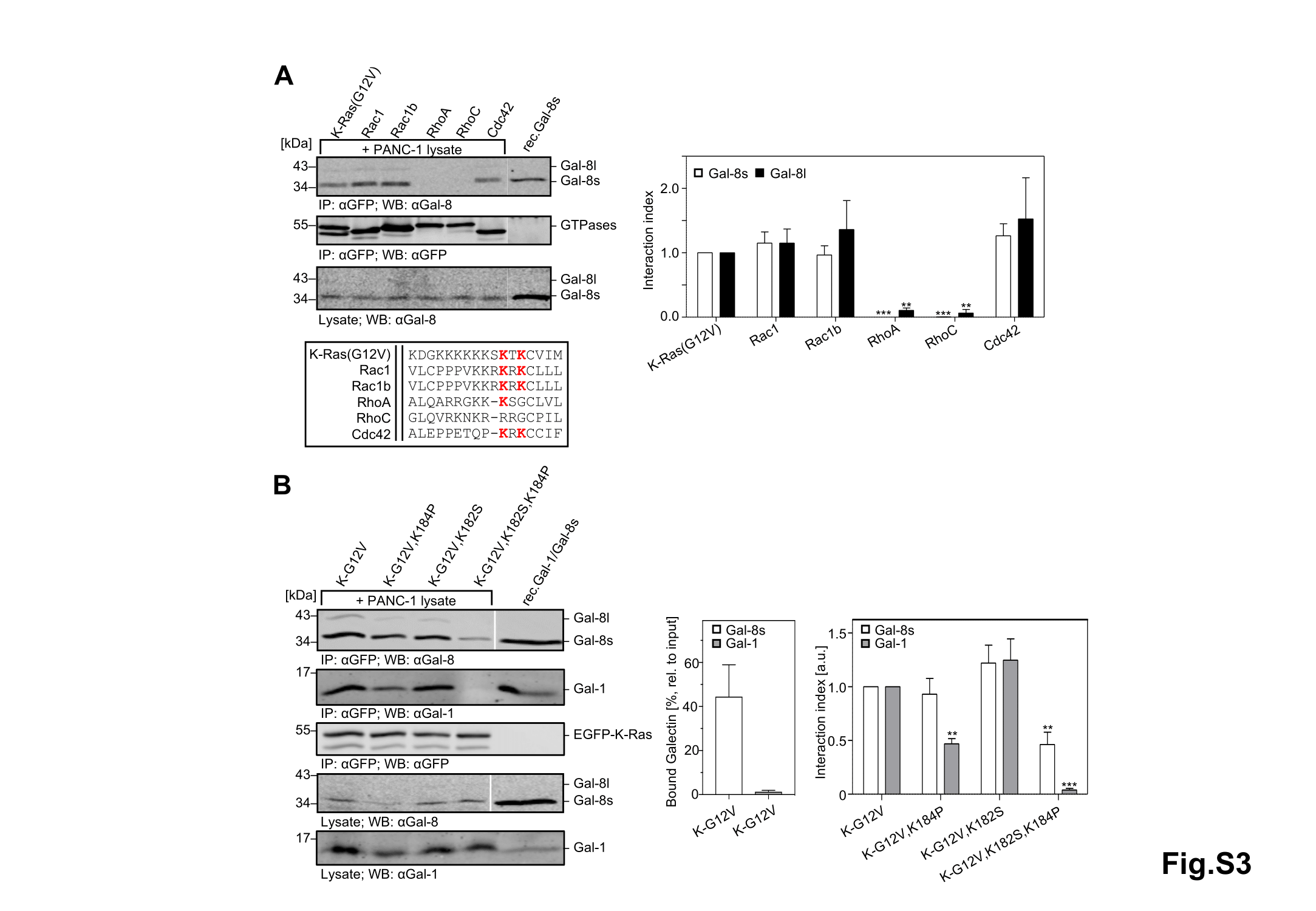


**Figure S3.** Interaction of Galectin with GTPases. (**A**) Interaction of Galectin-8 with Rho-GTPases**.** HEK293 cells were transiently transfected with plasmids encoding for EGFP, EGFP-tagged K-Ras(G12V), and wildtype EGFP- Rac1, -Rac1b, -RhoA, -RhoC and -Cdc42. Cells were lysed after 48 h. Equal amounts of EGFP- proteins were immunoprecipitated from each protein lysate using anti-GFP μMacs bead. 2 mg of PANC-1 lysate was added for 2 h to co-precipitate endogenous Galectins. The upper panel shows the amount of co-precipitated Galectin-8 and the middle panel the amount of the precipitated GTPases. The lower blot represents the amount of Galectin-8 in the PANC-1 lysate as an input control. The chart below displays the amino acid sequence within the hypervariable region of the GTPases with the lysines adjacent to the farnesylated cysteine denoted in bold. The diagram on the right side exposes the interaction index of Galectin-8 long (black) and short (white) with the GTPases as the mean ± SEM (*n* = 3). Galectin-8 showed a similar *in vitro* interaction to the three GTPases exhibiting two lysines next to the C-terminal cysteine as in K-Ras, namely Rac1, Rac1b and Cdc42, but lacked interaction with RhoA and RhoC. Thus, these data emphasizes the importance of the two lysines for the interaction. (**B**) Interaction of Galectin isoforms with EGFP-K-Ras mutants. HEK293 cells were transiently transfected with plasmids encoding for EGFP-tagged K-Ras: (G12V), (G12V,K184P), (G12V,K182S), or (G12V,K182S,K184P). Cells were lysed and interaction analyses were performed as described in (**A**). The precipitates were analyzed for simultaneous co-precipitation of Galectin-8 and Galectin-1 using western blotting. The upper panel of the blots shows co-precipitated Gal-8l and Gal-8s, the second panel co-precipitated Gal-1, and the third panel illustrates the immunoprecipitated EGFP-K-Ras proteins. The lower blots show detection of Gal-8 and Gal- 1 in 1/20 of each PANC-1 lysate, respectively. Recombinant Gal-8s (30 ng) and Gal-1 (40 ng) were used to control antibody specificity. The left bar graph shows the percentage of Gal-8s (white column) and Gal-1 (grey column) co-precipitated with EGFP-K-Ras (G12V) relative to the amount of Gal-8s and Gal-1 present in 2 mg of Panc-1 lysate, respectively. The input and bound amount of the Galectins was calculated by densitometric analysis of representative western blots using the amount of the rec.Gal-8s (30 ng) and Gal-1 (40 ng) as reference, set to 100 %. The percent bound Galectin is given as mean ± SEM, *n* = 3. The bar graph on the right displays the interaction index of Galectin-8 short (white) and Galectin-1 (grey) with EGFP-K-Ras quantified as described in (A). The interaction index is given as the mean ± SEM (** *p* ≤ 0.01; *** *p* ≤ 0.001, *n* = 5). These results demonstrate that the interaction of Galectin-1, which structurally resembles the N-CRD of Galectin-8, with K-Ras is also highly dependent on the two lysines next to the farnesylated cysteine. Removal of lysine184 alone already downregulates binding by ~50 %.


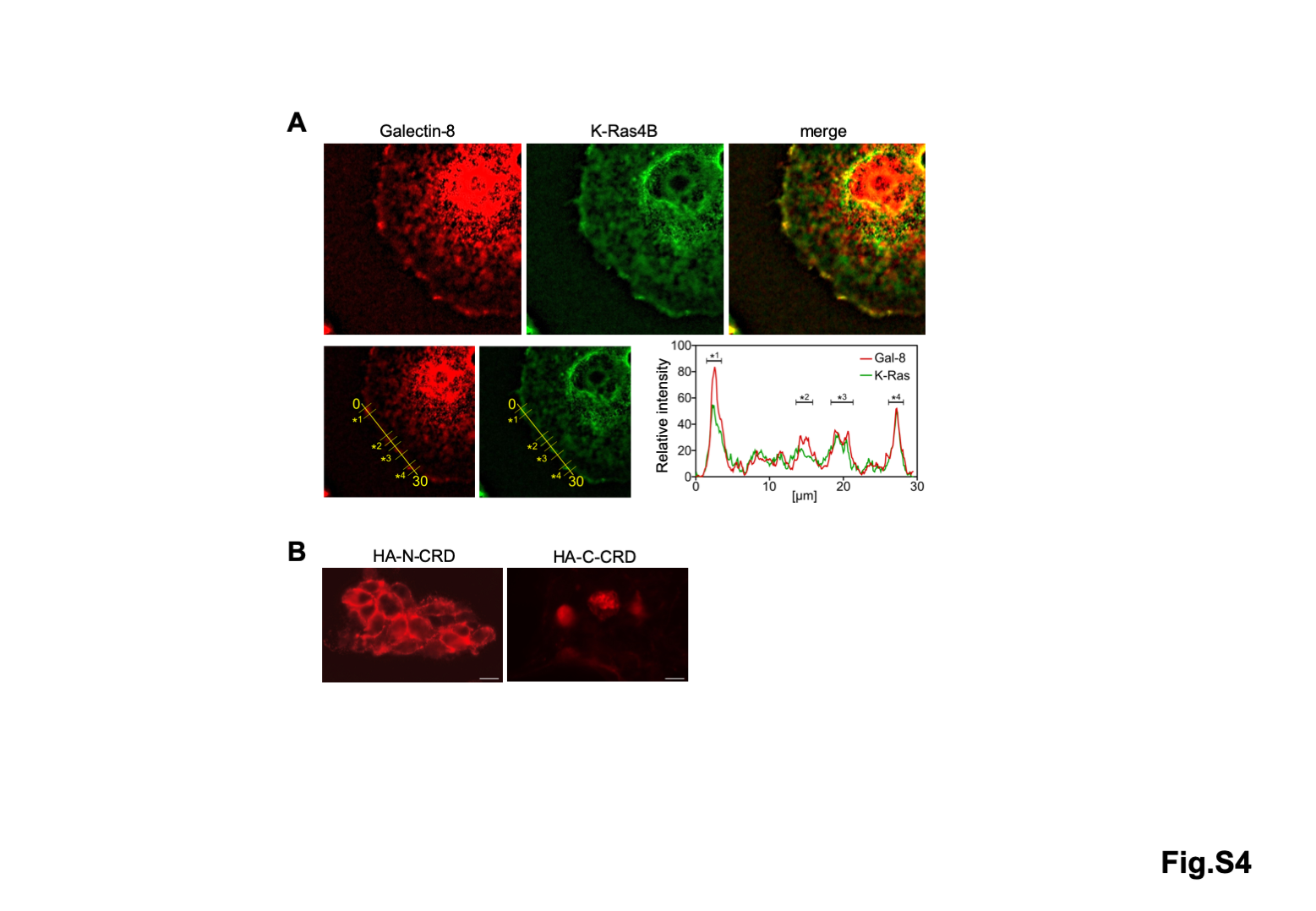


**Figure S4.** Immunofluorescence analysis of Galectin-8 and its CRDs**.** (**A**) Co-localization of K-Ras and Galectin-8. PANC-1 cells were fixed with paraformaldehyde and co-stained with anti-galectin 8 and Cy3-conjugated secondary antibody (left, red) and anti-K-Ras4B and Alexa-488 antibody (middle, green). A merged image is shown on the right. Microscopy was performed with an inverse fluorescence Leica DMi8 S microscope and Thunder imaging software. The fluorescence intensities of the marked 30 µm area at the membrane was quantified using Fiji software. Spots with intense yellow staining in the merge image were marked as *1, *2, *3, *4. The relative intensities are shown in the diagram. These images confirmed the co-localization of Galectin-8 and K-Ras at the plasma membrane. (**B**) Localization of N-CRD and C-CRD. Plasmids encoding for HA-N-CRD or HA-C-CRD were transiently expressed in HEK293 for 48 h. Cells were fixed with paraformaldehyde and HA-N-CRD (left) or HA-C-CRD (right) were stained with anti-HA-Tag and Cy3-conjugated secondary antibody. Microscopy was performed with an inverse fluorescence Olympus IX81 microscope. Bar: 10 µm. HA-N-CRD is mainly localized in the membrane of the cells, whereas HA-C-CRD is diffusely distributed in the cytoplasm near the nucleus.

Supplementary Materials

Antibodies, inhibitors, recombinant proteins

Rabbit polyclonal/monoclonal antibodies

Phospho-Akt (Ser473) (#9271), Phospho-p44/42 MAPK (Erk1/2) (Thr202/Tyr204) (#4370), and HA-Tag (#3724) were purchased from Cell Signaling Technology (Frankfurt am Main, Germany), GFP (FL) (#sc-8334) was from Santa Cruz Biotechnology (Heidelberg, Germany), Caveolin (#610059) from BD Transduction Laboratories (Heidelberg, Germany), and K-Ras (#12063-1-AP), and K-Ras2B-specific (#16155-1-AP) were acquired from Proteintech (Manchester, United Kingdom). Goat polyclonal antibodies Galectin-3 (#AF1197) and Galectin-1 (#AF1152) were purchased from Bio-Techne/R&D Systems (Wiesbaden, Germany).

Mouse monoclonal antibodies

Akt (pan) (#2920), p44/42 MAP Kinase (#4696), and HA-Tag (6E2) (#2367) were from Cell Signaling Technology (Frankfurt am Main, Germany), Galectin-8 (C-8) (#sc-377133), and K-Ras (F-234) (#sc-30) from Santa Cruz Biotechnolgy (Heidelberg, Germany), GFP-HRP (#130-091-833) from Miltenyi Biotec (Bergisch Gladbach, Germany), and panRas (Ab-3) (#OP40) from Merck (Darmstadt, Germany).

Secondary antibodies

CF^®^680 goat anti-mouse IgG (#20065-1) and CF^®^680 goat anti-rabbit IgG (#20067-1) were purchased from Biotium (Fremont, USA). IRDye^®^ 680LT donkey anti-goat IgG (#926-68024), IRDye^®^ 800CW goat anti-mouse IgG (#926-32210), IRDye^®^ 800CW goat anti-rabbit IgG (#926-32211), IRDye^®^ 800CW donkey anti-goat IgG (#926-32214) were acquired from LI-COR (Bad Homburg, Germany). Peroxidase-conjugated horse anti-mouse IgG (#7076) and goat anti-rabbit IgG (#7074) were from Cell Signaling Technology (Frankfurt am Main, Germany).

Recombinant proteins and inhibitors

Recombinant (rec.) human epidermal growth factor (#AF-100-15) was obtained from PeproTech (Hamburg, Germany). Rec. human Galectin-1 (#1152-GA), rec. human Galectin-3 (#1154-GA), rec. human Galectin-8 short (#1305-GA and #1305-GA/CF) were acquired from Bio-Techne/R&D Systems (Wiesbaden, Germany) and rec. K-Ras^His^ (#PR-239) was purchased from Jena Bioscience (Jena, Germany). Mitomycin-C was purchased from Santa Cruz Biotechnolgy (Heidelberg, Germany) and MG-132 from Calbiochem/Merck (Darmstadt, Germany). All other chemicals were of analytical grade and obtained from standard suppliers.

Plasmids

Expression plasmids for ectopic expression of EGFP-K-Ras (G12V), -H-Ras (G12V), -N-Ras (G12V), -Rac1, -Rac1b, -RhoA, -RhoC, -Cdc42 were produced ligating the coding sequence in frame into pEGFP-C vectors (Clontech, Heidelberg, Germany) as described in [1–3]. N-terminally HA-tagged K-Ras (G12V) was cloned as an EcoRV/NotI fragment into pVL1393/SmaI/NotI for expression in baculovirus-infected Spodoptera frugiperda (Sf) 9 insect cells or pET23d (+)/NcoI (Klenow)/NotI for expression in E.coli BL21 (DE3) (New England Biolabs, Frankfurt, Germany). His-tagged K-Ras (G12V) was generated by PCR using the His-K-Ras primers listed in Tab. 2 and cloned as BamHI fragment into pVL1393. Mutants of pEGFP-C3/K-Ras (G12V), pEGFP-C3/H-Ras (G12V) and pEGFP-C3/N-Ras (G12V) were created using the QuikChange II XL site-directed mutagenesis kit (Stratagene, Berlin, Germany) using the primers listed in Tab. 1. Additional mutants of pEGFP-C3/K-Ras (G12V) (see Figure 5) were created by PCR using primers listed in Tab. 2 and cloned as PstI/BamHI fragments into pEGFP-C3 vector. The cDNAs of the two splice-variants of Galectin-8 (transcript variant 1, NCBI NM_006499.3 and variant 2, NCBI NM201543.1) were transcribed from mRNA of PANC-1 cells using oligo-(dT) primers and the SuperScript preamplification system (ThermoFisher Scientific, Langenselbold, Germany) and amplified via PCR using 0.5 U Taq Polymerase and Gal-8 primers (Table 2). PCR fragments were purified from agarose gels. DNA from the upper band corresponding to the long isoform of Galectin-8 (1145 bp) [Galectin-8 long (Gal-8l)] and the lower band corresponding to the shorter isoform (956 bp) [Galectin-8 short (Gal-8s)] were subcloned, reamplified by PCR (Gal-8 Kozak primer, Table 2) and cloned into pcDNA3 expression vector. For expression in baculovirus-infected Sf9 insect cells Gal-8l was inserted into the BamHI/NotI restriction sites of pVL1393. An N-terminally HA-tagged version of Gal-8l was created by PCR and cloned as a XbaI/NotI/ fragment into pVL1393. His-tagged Gal-8l was generated from HA-Gal-8l by using QuikChange II XL site-directed mutagenesis kit and specific primers listed in Tab. 1. To create cDNAs of the N-terminal and C-terminal CRD of Gal-8l, specific primers for in vitro mutagenesis and PCR listed in Table 1 and Table 2 were used. Both CRD fragments were cloned into the pCGN/HA expression plasmid. HA-tagged N-CRD or C-CRD fused to the hinge region was produced by PCRs with primers listed in Table 2. All sequences were verified by DNA sequencing (GATC Biotech, Konstanz, Germany).

**Table 1.** List of primers applied within *in vitro* mutagenesis experiments.

| Name | Primer Sequence |
| --- | --- |
| K-Ras(G12V,K184P) | 5′ Primer: GAAGTCAAAGACACCATGTGTAATTATGTAAGGATCCACCGGATCTAG  3′ Primer: TCAGATCCGGTGGATCCTTACATAATTACACATGGTGTCTTTGACTTC |
| K-Ras(G12V,K182S) | 5′ Primer: GAAAAAGAAGTCAAGCACAAAGTGTGTAATTATGTAAGGATCCACCGG  3′ Primer: CCGGTGGAACCTTACATAATTACACACTTTGTGCTTGACTTCTTTTTC |
| K-Ras(G12V,K182S, K184P) | 5′ Primer: GAAAAAGAAGTCAAGCACACCATGTGTAATTATGTAAGGATCCACCGGATCTAGATAACTG  3′ Primer: CAGTTATCTAGATCCGGTGGATCCTTACATAATTACACATGGTGTGCTTGACTTCTTTTTC |
| H-Ras (G12V,K185P) | 5′ Primer: CCCGGCTGCATGAGCTGCCCATGTGTGCTCTCC  3′ Primer: GGAGAGCACACATGGGCAGCTCATGCAGCCGGG |
| H-Ras (G12V,S183K) | 5′ Primer: CCCGGCTGCATGAAGTGCAAGTGTGTGCTCTCC  3′ Primer: GGAGAGCACACACTTGCACTTCATGCAGCCGGG |
| N-Ras(G12V,G183K,  P185K) | 5′ Primer: GGGACTCAGGGTTGTATGAAGTTGAAGTGTGTGGTGATGTAAGGATCCACCGG  3′ Primer: CCGGTGGATCCTTACATCACCACACACTTCAACTTCATACAACCCAGAGTCCC |
| N-Ras(G12V,P185K) | 5′ Primer: GACTCAGGGTTGTATGGGATTGAAGTGTGTGGTGATGTAAGGATC  3′ Primer: GATCCTTACATCACCACACACTTCAATCCCATACAACCCAGAGTC |
| N-CRD(Δ*Bam*HI) | 5′ Primer: CACCATTCCTGATCAGCTCGATCCTGGAACTTTGATTG  3′ Primer: CAATCAAAGTTCCAGGATCGAGCTGATCAGGAATGGTG |
| His-Gal-8 | 5′ Primer: CCCGGGTACCTTCTAGAATGCATCACCACCATCACCATCACCACCCTGACTATGCCAGCCTG  3′ Primer: CAGGCTGGCATAGTCAGGGTGGTGATGGTGATGGTGGTGATGCATTCTAGAAGGTACCCGGG |

**Table 2.** List of primers applied within PCR cloning experiments.

| Name | Primer Sequence |
| --- | --- |
| Gal-8 | 5′ Primer: AGCTGGAAAAGAATGATGTT  3′ Primer: TTTTGTAGCAGCTGTGTAGG |
| Gal-8 Kozak | 5′ Primer: GGGGGTACCGCCACCATGATGTTGTCCTTAAAC  3′ Primer: GGGGATATCCTACCAGCTCCTTACTTCCAG |
| K-Ras(G12V)PL-H | 5′ Primer: GGGCTGCAGCATATGACTGAATATAAAC  3′ Primer: CCCCCCGGATCCTTACATAATTACACACTTTGTCTTTGAGCAGCCGGGGCCACTCTCACCATCTTTGC |
| K-Ras(G12V)PL-N | 5′ Primer: GGGCTGCAGCATATGACTGAATATAAAC  3′ Primer: CCCCCCGGATCCTTACATAATTACACACTTTGTCTTTGAACAACCCTGAGTCCCATCACCATCTTTGC |
| K-Ras(G12V,K182S,  K184P)PL-H | 5′ Primer: GGGCTGCAGCATATGACTGAATATAAAC  3’ Primer: CCCCCCGGATCCTTACATAATTACACATGGTGTGCTTGAGCAGCCGGGGCCACTCTCACCATCTTTGC |
| K-Ras(G12V,K182S,  K184P)PL-N | 5′ Primer: GGGCTGCAGCATATGACTGAATATAAAC  3′ Primer: CCCCCCGGATCCTTACATAATTACACATGGTGTGCTTGAACAACCCTGAGTCCCATCACCATCTTTGC |
| HA-N-CRD | 5′ Primer: GGGGGATCCATGATGTTGTCCTTAAACAACCTACAGAAT  3′ Primer: GGGGATCCGCGGCCGCCTACGAGCTGAAGCTAAAACCAATTGAGTG |
| HA-C-CRD | 5’ Primer: GGGAATTCCGGATCCATGAGGCTGCCATTCGCTGCAAGGTTG  3′ Primer: GGGGATCCCTACCAGCTCCTTACTTCCAGTAAGTG |
| HA-N-CRD-hinge | 5′ Primer: GGGGGATCCATGATGTTGTCCTTAAACAACCTACAGAAT  3′ Primer: CCCGCGGCCGCCTACTTTGACACATAGTTCATAGGTGGTAT |
| HA-C-CRD-hinge | 5′ Primer: GGGGGATCCATGGACTTACAAAGTACCCAAGCATCT  3′ Primer: CCCGCGGCCGCCTACCAGCTCCTTACTTCCAGTAAGTGGAT |
| His-K-Ras | 5′ Primer: CCCATCGAATTCCTGCAGATGCATCACCATCACCATCACCATCACGCCCATATGACTGAATATAAACTTG  3′ Primer: GGGGGATCCTTACATAATTACACACTTTGT |

Buffers

Gold-Lysis buffer

20 mM Tris HCl pH 7.9, 50 mM NaCl, 10 % (v/v) glycerol, 1 % (v/v) Triton X-100, 0.05 % (m/v) sodium deoxycholate, 1.39 mM pefabloc, 15 µM aprotinin, 21 µM leupeptin, 50 µM trypsin inhibitor.

HEPES buffer

50 mM HEPES, pH 7.6, 8.6 % (m/v) sucrose, 10 mM EDTA, 10 mM EGTA, 1 mM phenylmethylsulfonyl fluoride (PMSF), 80 µM leupeptin, 60 µM pepstatin A, 0.4 mg/ml soybean trypsin inhibitor. IP buffer: 50 mM Tris pH 7.6, 200 mM NaCl, 20 mM MgCl_2_, 0.5 % (V/V) NP-40, 0.5 % (m/V) sodium deoxycholate, 1 mM DTT, 1.39 mM pefabloc, 15 µM aprotinin, 21 µM leupeptin, 50 µM soybean trypsin inhibitor, 10 mM sodium pyrophosphat, 25 mM β-glycerophosphate, 2 mM sodium orthovanadate.

His buffer

50 mM Tris pH 7.6; 200 mM NaCl, 20 mM MgCl_2_, 0.5 % (v/v) NP-40, 0.5 % (m/v) sodium deoxycholate, 10 mM imidazole, 0.5 µM GDP, 1 mM DTT, 1.39 mM pefabloc, 15 µM aprotinin, 21 µM leupeptin, 50 µM soybean trypsin inhibitor.

Ni-NTA washing buffer

50 mM Tris pH 7.6, 20 mM MgCl_2_, 200 mM NaCl, 0.5 µM GDP, 20 mM imidazole, 1.39 mM pefabloc, 15 µM aprotinin, 21 µM leupeptin, 50 µM soybean trypsin inhibitor.

HA-binding buffer

50 mM Tris HCl pH 7.5, 150 mM NaCl, 0.5 % (v/v) Triton X-100, 5 mM MgCl_2_, 0.5 µM GDP, 1 mM DTT, 1 mM PMSF.

MACS washing buffer 1

50 mM Tris HCl pH 8.0, 150 mM NaCl, 1 % (v/v) Igepal CA-630, 0.5 % (m/v) sodium deoxycholate, 0.1 % (v/v) SDS.

Elution buffer

50 mM Tris HCl pH 6.8, 10 % glycerol, 50 mM DTT, 1 % (v/v) SDS, 1 mM EDTA, 0.005 % bromophenol blue.

Reference

1. Dreissigacker, U.; Mueller, M.S.; Unger, M.; Siegert, P.; Genze, F.; Gierschik, P.; Giehl, K. Oncogenic K-Ras down-regulates Rac1 and RhoA activity and enhances migration and invasion of pancreatic carcinoma cells through activation of p38. *Cell Signal.* **2006**, *18*, 1156–1168, doi:10.1016/j.cellsig.2005.09.004.
2. Hage, B.; Meinel, K.; Baum, I.; Giehl, K.; Menke, A. Rac1 activation inhibits E-cadherin-mediated adherens junctions via binding to IQGAP1 in pancreatic carcinoma cells. *Cell Commun. Signal.* **2009**, *7*, 23, doi:10.1186/1478-811x-7-23.
3. Schreiber, S.C.; Giehl, K.; Kastilan, C.; Hasel, C.; Muhlenhoff, M.; Adler, G.; Wedlich, D.; Menke, A. Polysialylated NCAM represses E-cadherin-mediated cell-cell adhesion in pancreatic tumor cells. *Gastroenterology* 2008, *134*, 1555–1566, doi:10.1053/j.gastro.2008.02.023.
