# Supplementary material for: Galectin-8 binds to the Farnesylated C-terminus of K-Ras4B and Modifies Ras/ERK Signaling and Migration in Pancreatic and Lung Carcinoma Cells
Source: Cancers (Basel). 2019 Dec 20;12(1):30. doi: 10.3390/cancers12010030 (PMC7017085; doi:10.3390/cancers12010030)
Supplement: Supplementary file 1 [file cancers-12-00030-s001.zip › cancers-672634-Suppl-final/cancers-672634-western blot figures.pdf]

Fig.1

A

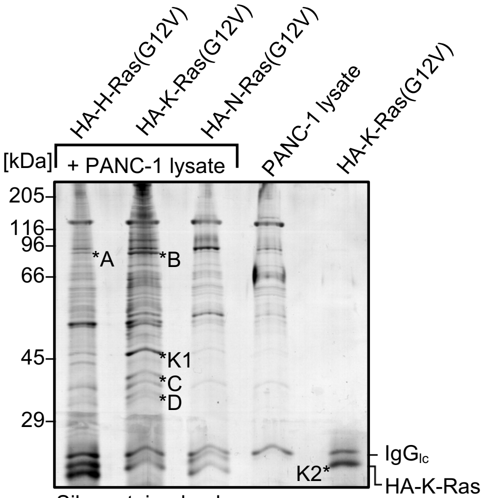

Silver-stained gel

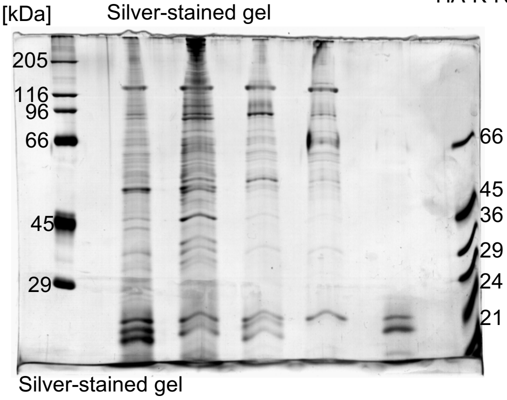

Silver-stained gel

Fig.1

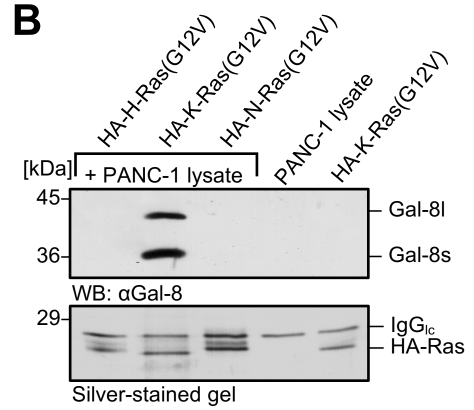
B
HA-H-Ras(G12V)
HA-K-Ras(G12V)
HA-N-Ras(G12V)
PANC-1 lysate
HA-K-Ras(G12V)
[kDa]
+ PANC-1 lysate
45
36
Gal-8l
Gal-8s
WB: αGal-8
29
IgGlc
HA-Ras
Silver-stained gel

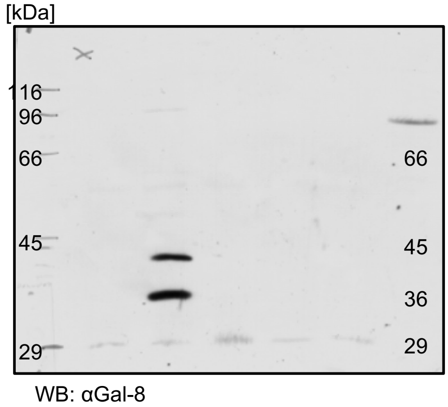
[kDa]
116
96
66
45
29
66
45
36
29
WB: αGal-8

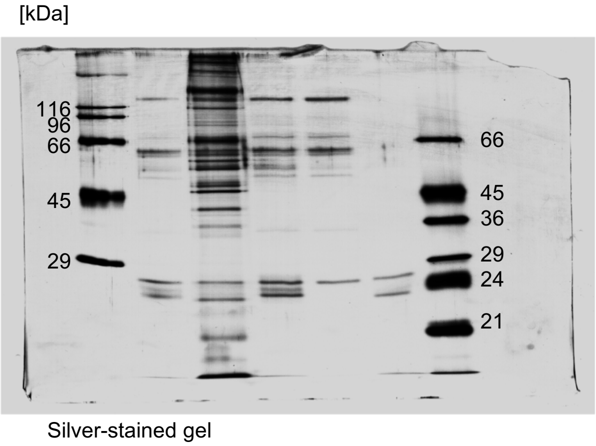
[kDa]
116
96
66
45
29
66
45
36
29
24
21
Silver-stained gel

Fig.1

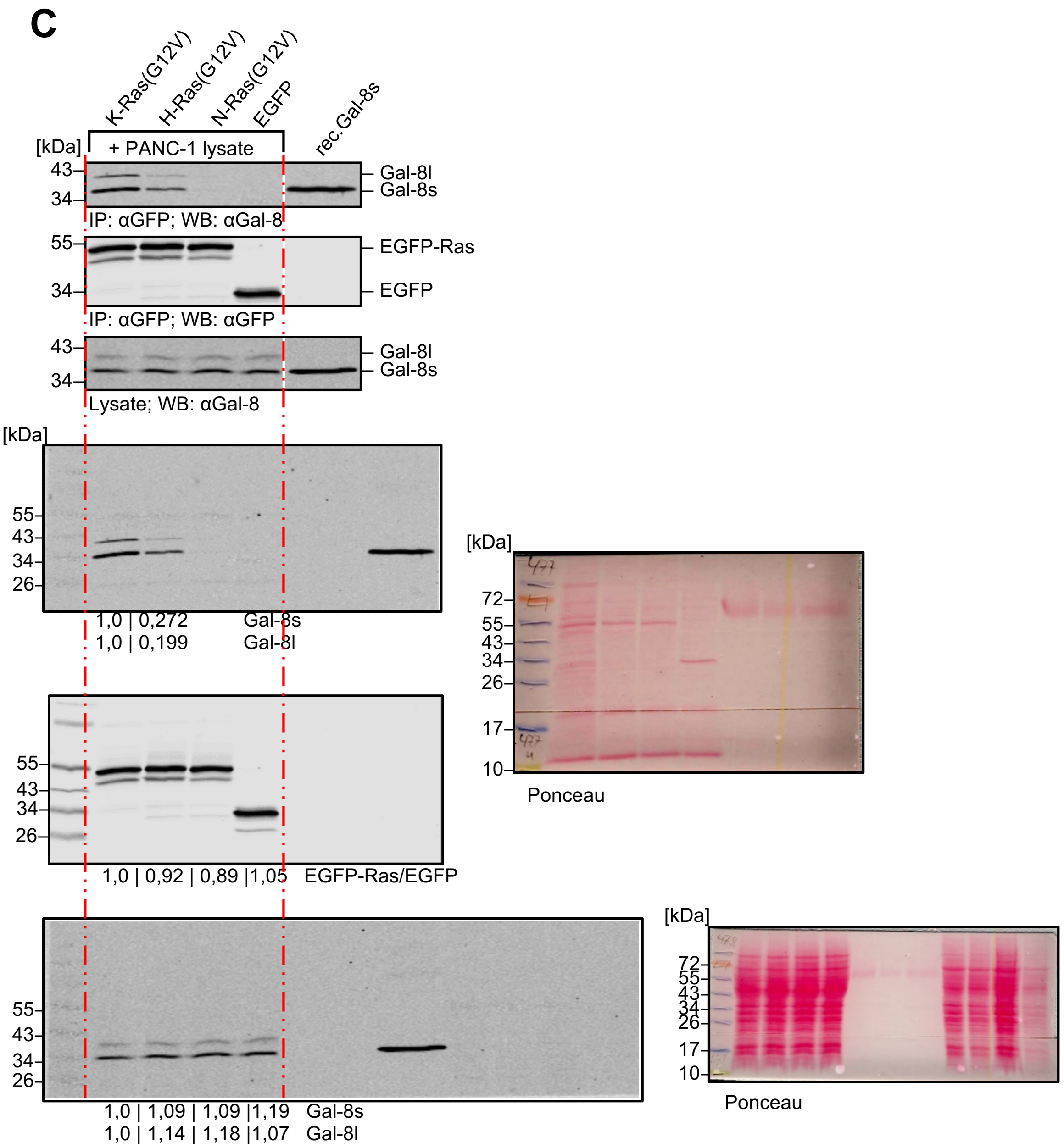
C
K-Ras(G12V)
H-Ras(G12V)
N-Ras(G12V)
EGFP
rec.Gal-8s
+ PANC-1 lysate
[kDa]
43
34
Gal-8l
Gal-8s
IP: αGFP; WB: αGal-8
55
34
EGFP-Ras
EGFP
IP: αGFP; WB: αGFP
43
34
Gal-8l
Gal-8s
Lysate; WB: αGal-8
[kDa]
55
43
34
26
1,0 | 0,272 Gal-8s
1,0 | 0,199 Gal-8l
[kDa]
72
55
43
34
26
17
10
Ponceau
55
43
34
26
1,0 | 0,92 | 0,89 |1,05 EGFP-Ras/EGFP
55
43
34
26
1,0 | 1,09 | 1,09 |1,19 Gal-8s
1,0 | 1,14 | 1,18 |1,07 Gal-8l
[kDa]
72
55
43
34
26
17
10
Ponceau

Fig.1

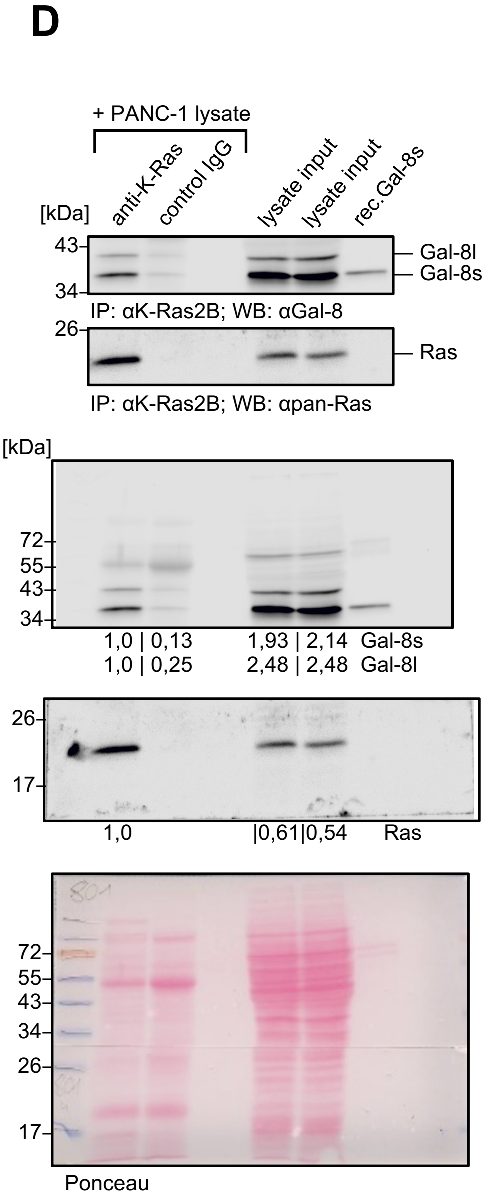
D
+ PANC-1 lysate
anti-K-Ras
control IgG
lysate input
lysate input
rec.Gal-8s
[kDa]
43
34
Gal-8l
Gal-8s
IP: αK-Ras2B; WB: αGal-8
26
Ras
IP: αK-Ras2B; WB: αpan-Ras
[kDa]
72
55
43
34
1,0 | 0,13
1,93 | 2,14
Gal-8s
1,0 | 0,25
2,48 | 2,48
Gal-8l
26
17
1,0
|0,61|0,54
Ras
72
55
43
34
26
17
Ponceau

Fig.2

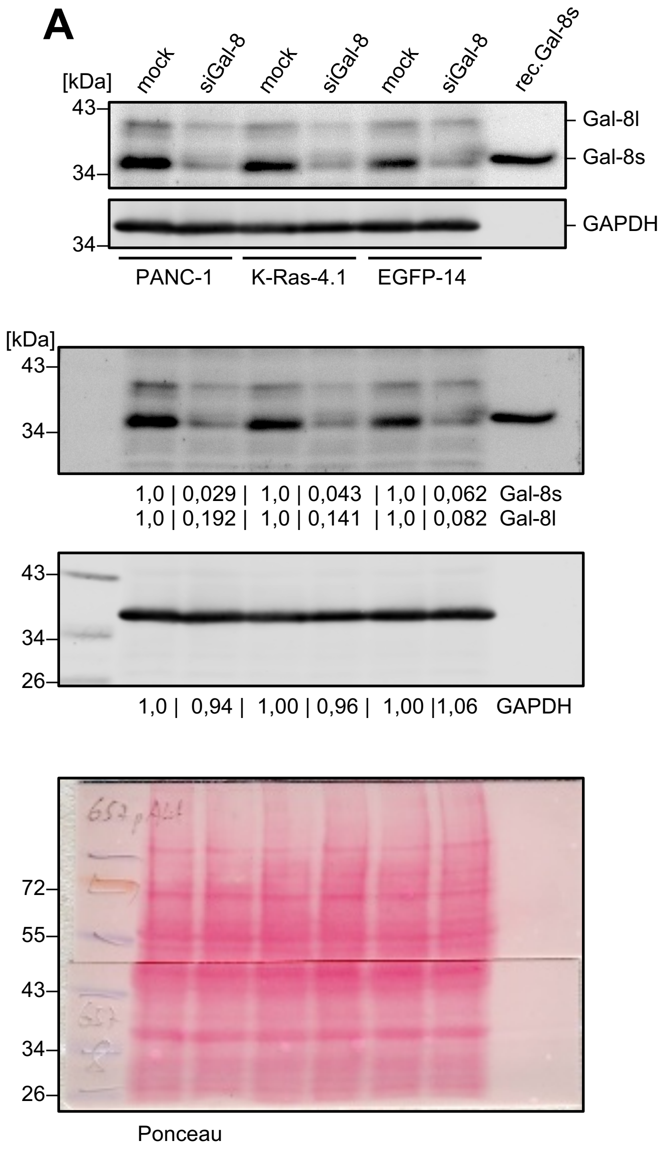

A
mock
siGal-8
mock
siGal-8
mock
siGal-8
rec.Gal-8s
[kDa]
43
34
Gal-8l
Gal-8s
34
GAPDH
PANC-1
K-Ras-4.1
EGFP-14
[kDa]
43
34
1,0 | 0,029 | 1,0 | 0,043 | 1,0 | 0,062 Gal-8s
1,0 | 0,192 | 1,0 | 0,141 | 1,0 | 0,082 Gal-8l
43
34
26
1,0 | 0,94 | 1,00 | 0,96 | 1,00 |1,06 GAPDH
72
55
43
34
26
Ponceau

Fig.2

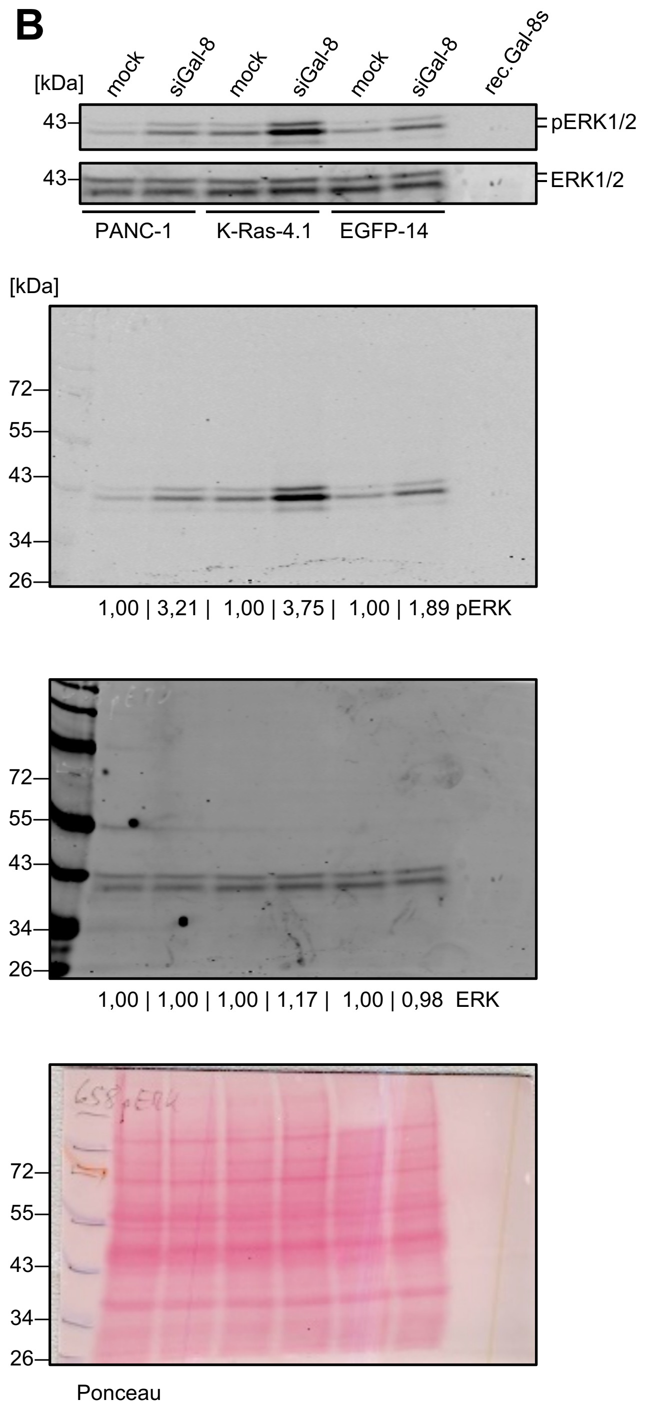

B
[kDa]
mock
siGal-8
mock
siGal-8
mock
siGal-8
rec.Gal-8s
43
pERK1/2
43
ERK1/2
PANC-1
K-Ras-4.1
EGFP-14
[kDa]
72
55
43
34
26
1,00 | 3,21 | 1,00 | 3,75 | 1,00 | 1,89 pERK
72
55
43
34
26
1,00 | 1,00 | 1,00 | 1,17 | 1,00 | 0,98 ERK
72
55
43
34
26
Ponceau

Fig.2

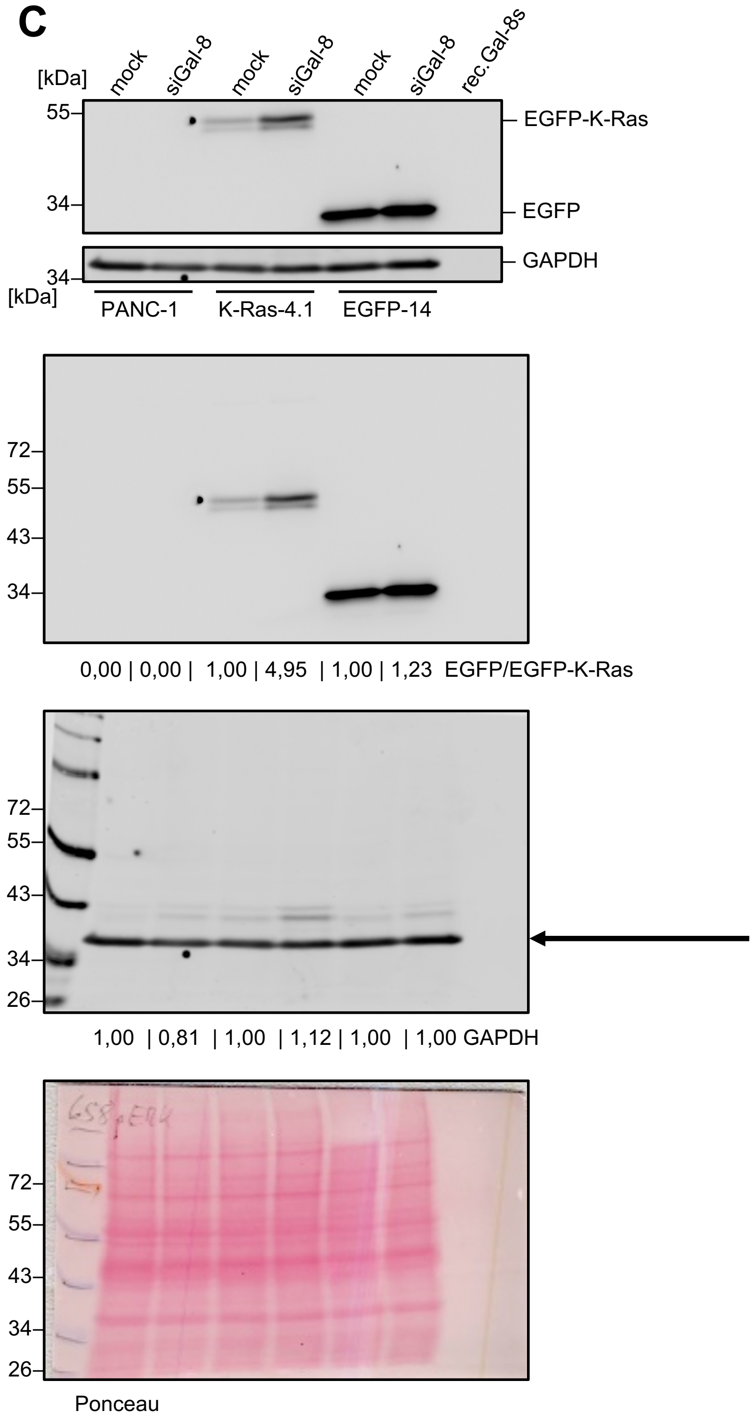
C
[kDa]
mock
siGal-8
mock
siGal-8
mock
siGal-8
rec.Gal-8s
55
34
EGFP-K-Ras
EGFP
GAPDH
34
[kDa]
PANC-1
K-Ras-4.1
EGFP-14
72
55
43
34
0,00 | 0,00 | 1,00 | 4,95 | 1,00 | 1,23 EGFP/EGFP-K-Ras
72
55
43
34
26
1,00 | 0,81 | 1,00 | 1,12 | 1,00 | 1,00 GAPDH
72
55
43
34
26
Ponceau

Fig.2

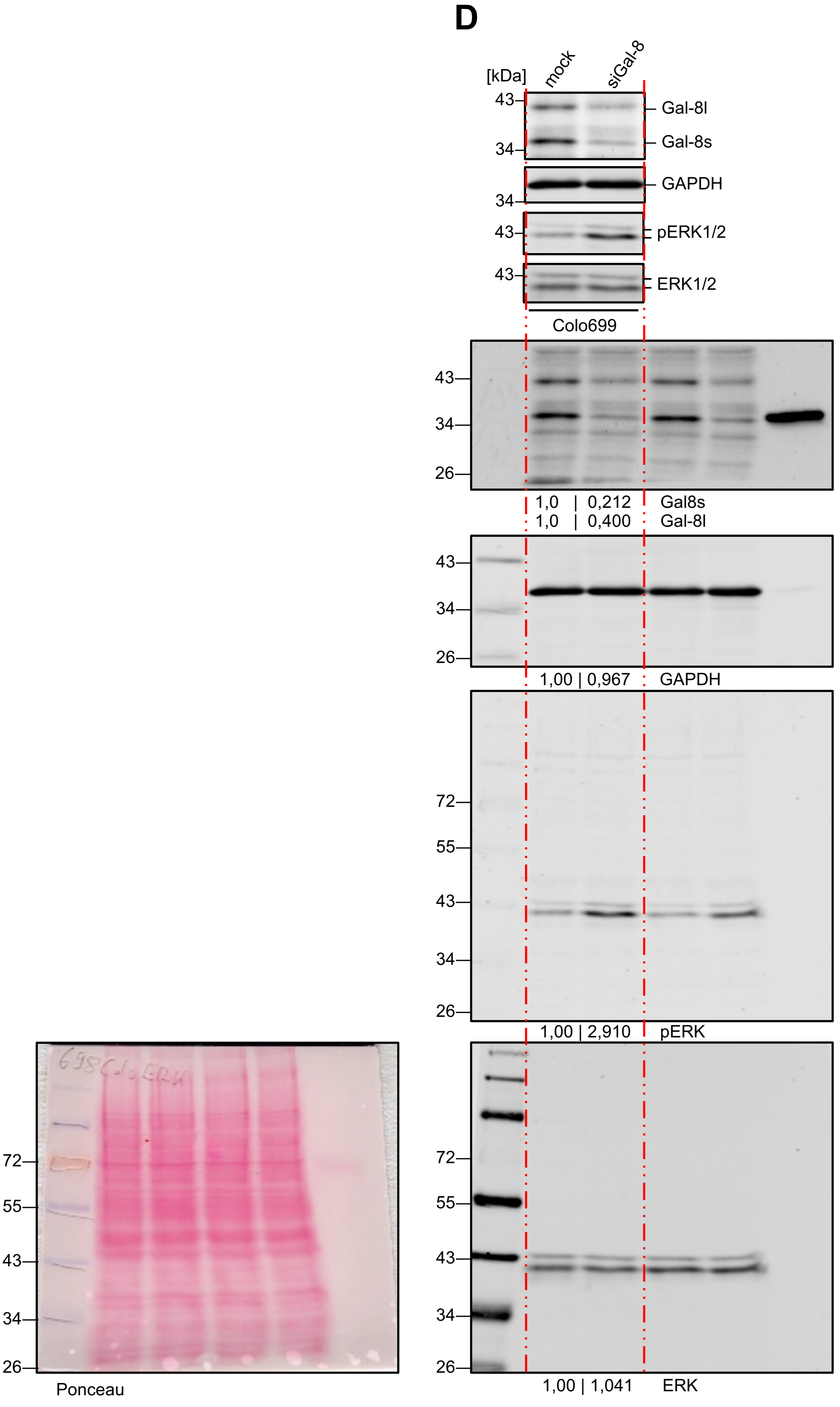
D
[kDa]
mock
siGal-8
43
34
Gal-8l
Gal-8s
GAPDH
pERK1/2
ERK1/2
Colo699
26
72
55
1,0 | 0,212 Gal8s
1,0 | 0,400 Gal-8l
1,00 | 0,967 GAPDH
1,00 | 2,910 pERK
1,00 | 1,041 ERK
698 Colo ERK
Ponceau

Fig.2

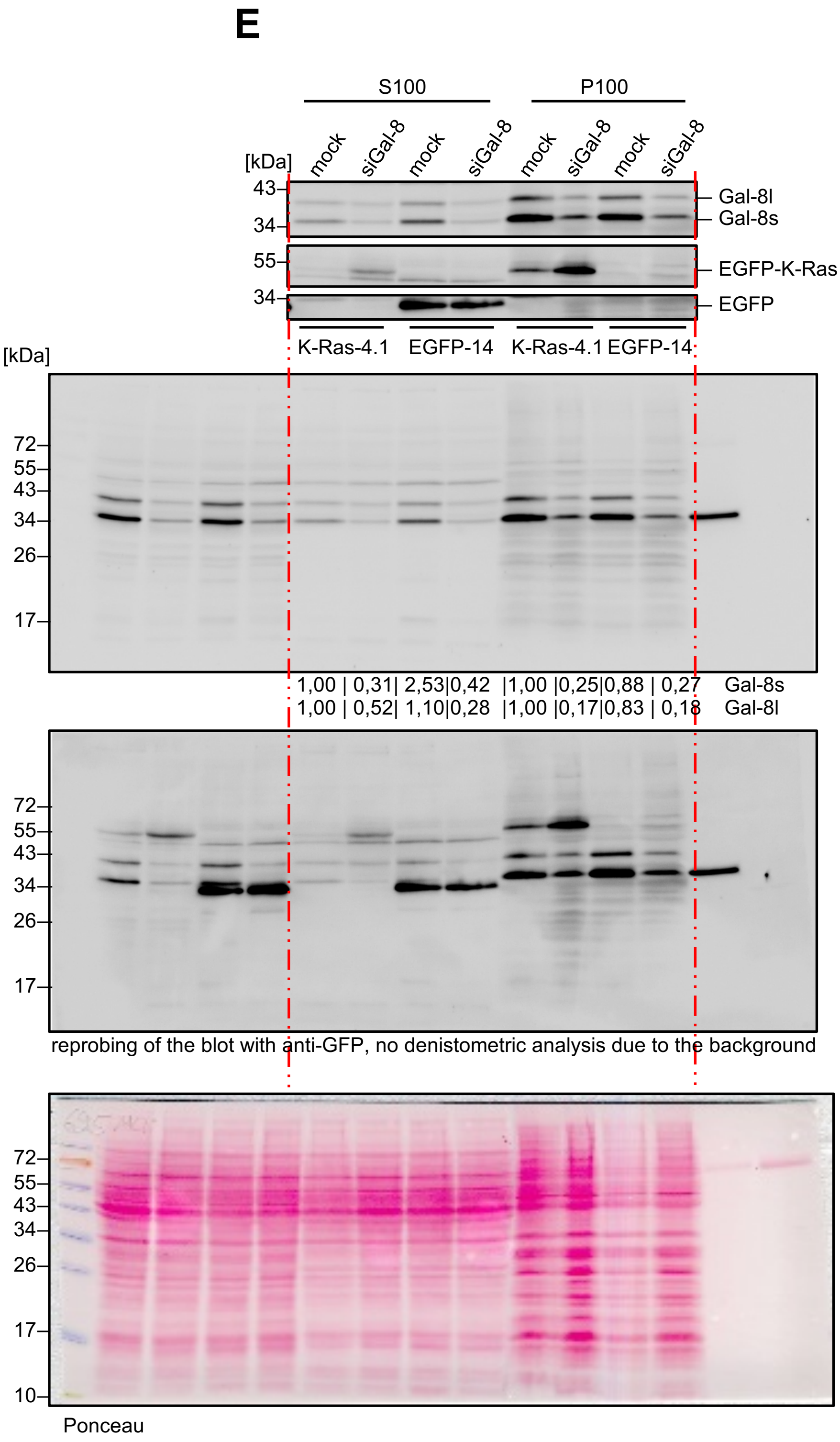
E
S100
P100
mock
siGal-8
mock
siGal-8
mock
siGal-8
mock
siGal-8
[kDa]
43
34
55
34
Gal-8l
Gal-8s
EGFP-K-Ras
EGFP
K-Ras-4.1
EGFP-14
K-Ras-4.1
EGFP-14
[kDa]
72
55
43
34
26
17
1,00 | 0,31| 2,53|0,42 |1,00 |0,25|0,88 | 0,27 Gal-8s
1,00 | 0,52| 1,10|0,28 |1,00 |0,17|0,83 | 0,18 Gal-8l
72
55
43
34
26
17
reprobing of the blot with anti-GFP, no denistometric analysis due to the background
72
55
43
34
26
17
10
Ponceau

Fig.3

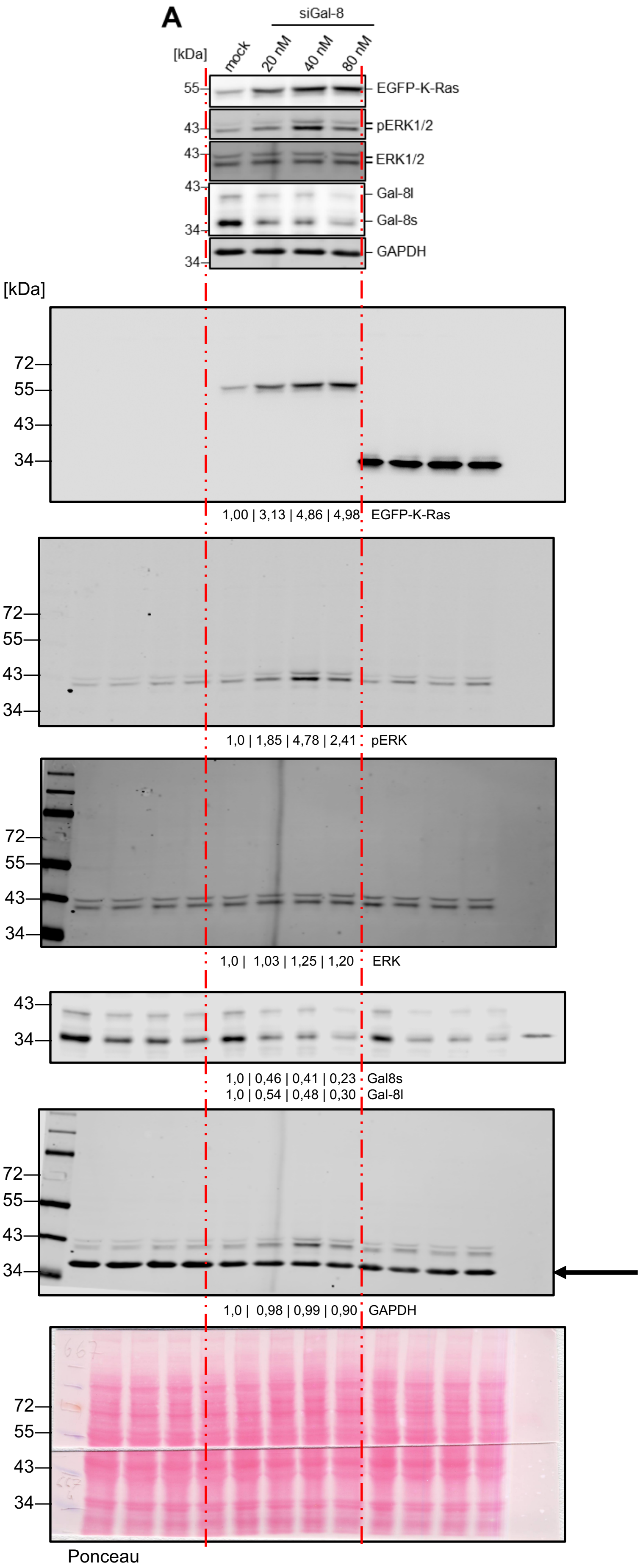
A
siGal-8
[kDa]
mock
20 nM
40 nM
80 nM
55
43
43
43
34
34
EGFP-K-Ras
pERK1/2
ERK1/2
Gal-8l
Gal-8s
GAPDH
[kDa]
72
55
43
34
1,00 | 3,13 | 4,86 | 4,98 EGFP-K-Ras
1,0 | 1,85 | 4,78 | 2,41 pERK
1,0 | 1,03 | 1,25 | 1,20 ERK
1,0 | 0,46 | 0,41 | 0,23 Gal8s
1,0 | 0,54 | 0,48 | 0,30 Gal-8l
1,0 | 0,98 | 0,99 | 0,90 GAPDH
Ponceau

Fig.3

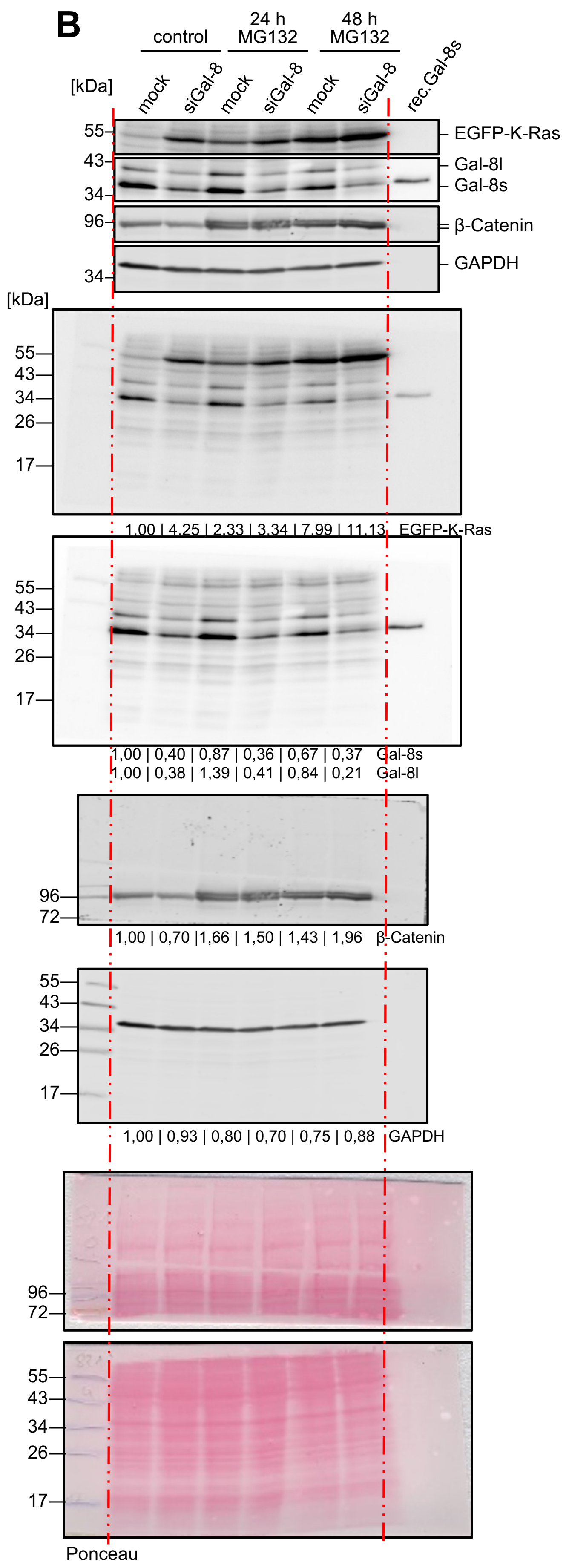
B
control
24 h MG132
48 h MG132
[kDa]
mock
siGal-8
mock
siGal-8
mock
siGal-8
rec.Gal-8s
EGFP-K-Ras
Gal-8l
Gal-8s
β-Catenin
GAPDH
[kDa]
1,00 | 4,25 | 2,33 | 3,34 | 7,99 | 11,13 EGFP-K-Ras
1,00 | 0,40 | 0,87 | 0,36 | 0,67 | 0,37 Gal-8s
1,00 | 0,38 | 1,39 | 0,41 | 0,84 | 0,21 Gal-8l
1,00 | 0,70 |1,66 | 1,50 | 1,43 | 1,96 β-Catenin
1,00 | 0,93 | 0,80 | 0,70 | 0,75 | 0,88 GAPDH
Ponceau

Fig.4

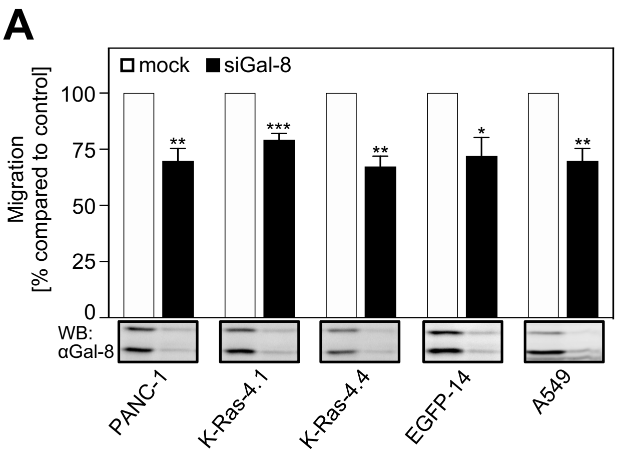
A
mock
siGal-8
Migration [% compared to control]
100
75
50
25
0
**
***
**
*
**
WB: αGal-8
PANC-1
K-Ras-4.1
K-Ras-4.4
EGFP-14
A549

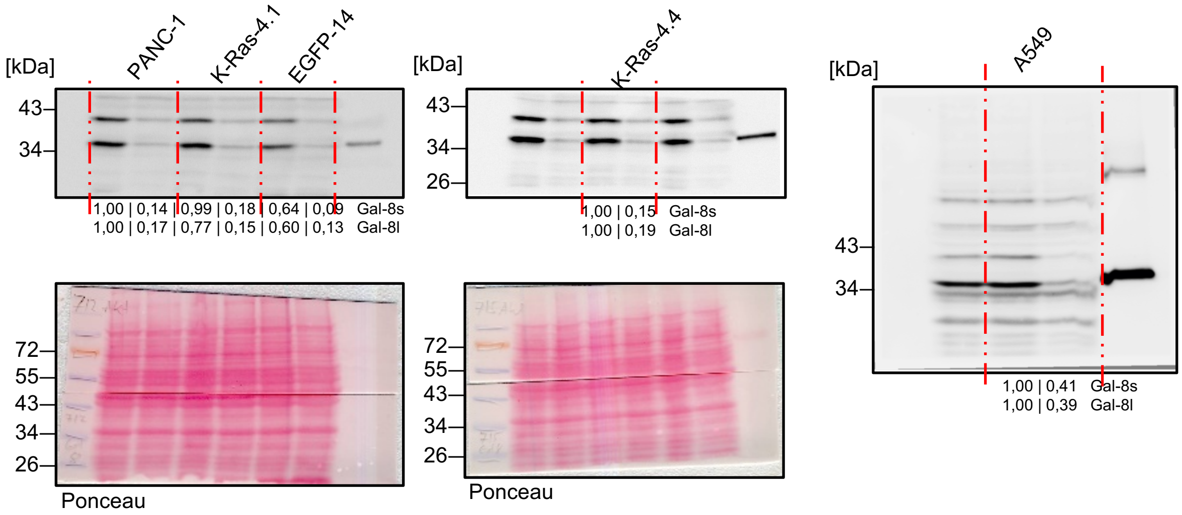
[kDa]
PANC-1
K-Ras-4.1
EGFP-14
43
34
1,00 | 0,14 | 0,99 | 0,18 | 0,64 | 0,09 Gal-8s
1,00 | 0,17 | 0,77 | 0,15 | 0,60 | 0,13 Gal-8l
72
55
43
34
26
Ponceau
[kDa]
K-Ras-4.4
43
34
26
1,00 | 0,15 Gal-8s
1,00 | 0,19 Gal-8l
72
55
43
34
26
Ponceau
[kDa]
A549
43
34
1,00 | 0,41 Gal-8s
1,00 | 0,39 Gal-8l

Fig.4

B

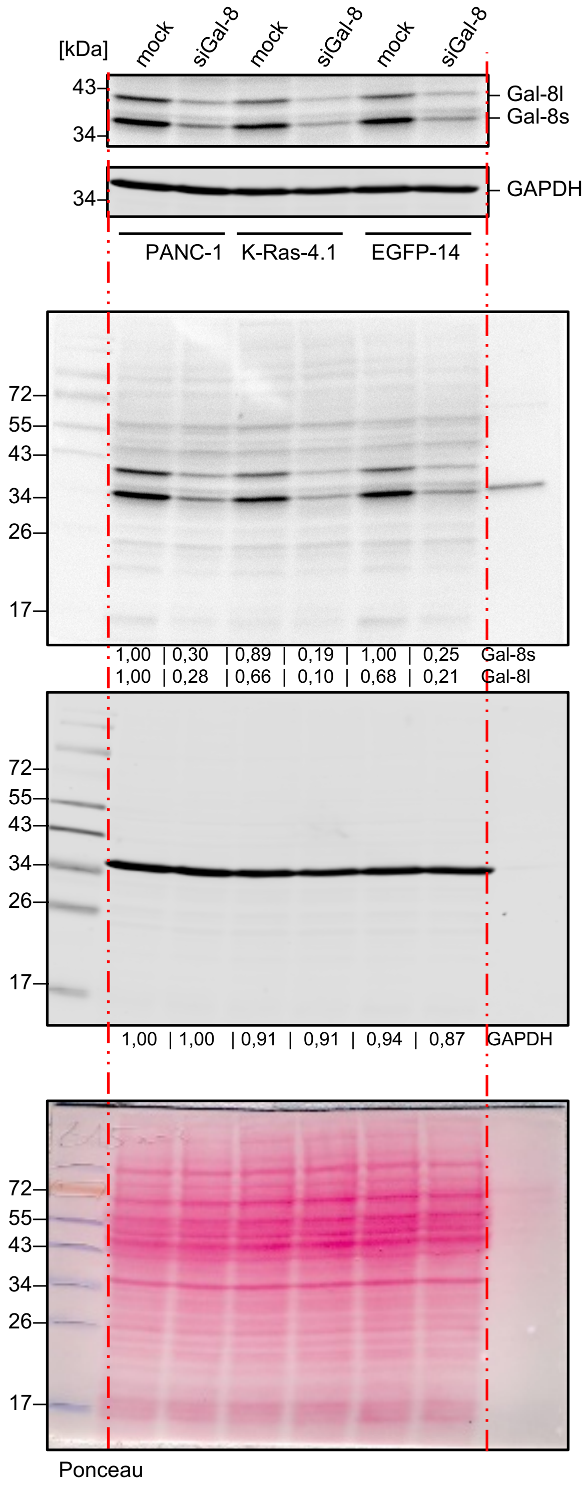
[kDa]
mock
siGal-8
mock
siGal-8
mock
siGal-8
43
34
Gal-8l
Gal-8s
34
GAPDH
PANC-1
K-Ras-4.1
EGFP-14
72
55
43
34
26
17
1,00 | 0,30 | 0,89 | 0,19 | 1,00 | 0,25 Gal-8s
1,00 | 0,28 | 0,66 | 0,10 | 0,68 | 0,21 Gal-8l
72
55
43
34
26
17
1,00 | 1,00 | 0,91 | 0,91 | 0,94 | 0,87 GAPDH
72
55
43
34
26
17
Ponceau

Fig.5

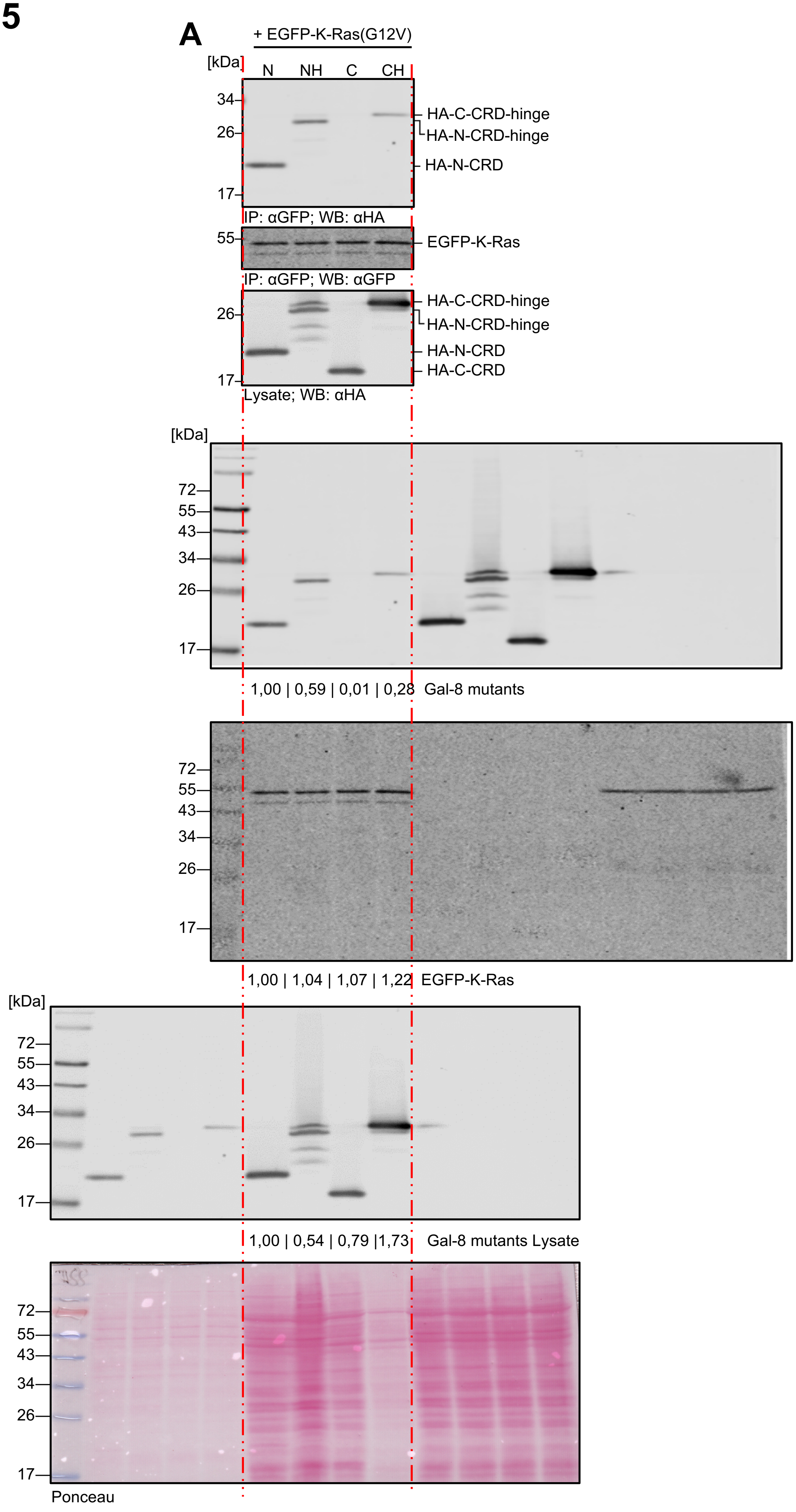
A
+ EGFP-K-Ras(G12V)
[kDa]
N
NH
C
CH
34
26
17
HA-C-CRD-hinge
HA-N-CRD-hinge
HA-N-CRD
IP: αGFP; WB: αHA
55
EGFP-K-Ras
IP: αGFP; WB: αGFP
26
17
HA-C-CRD-hinge
HA-N-CRD-hinge
HA-N-CRD
HA-C-CRD
Lysate; WB: αHA
[kDa]
72
55
43
34
26
17
1,00 | 0,59 | 0,01 | 0,28 Gal-8 mutants
72
55
43
34
26
17
1,00 | 1,04 | 1,07 | 1,22 EGFP-K-Ras
[kDa]
72
55
43
34
26
17
1,00 | 0,54 | 0,79 |1,73 Gal-8 mutants Lysate
72
55
43
34
26
17
Ponceau

Fig.5

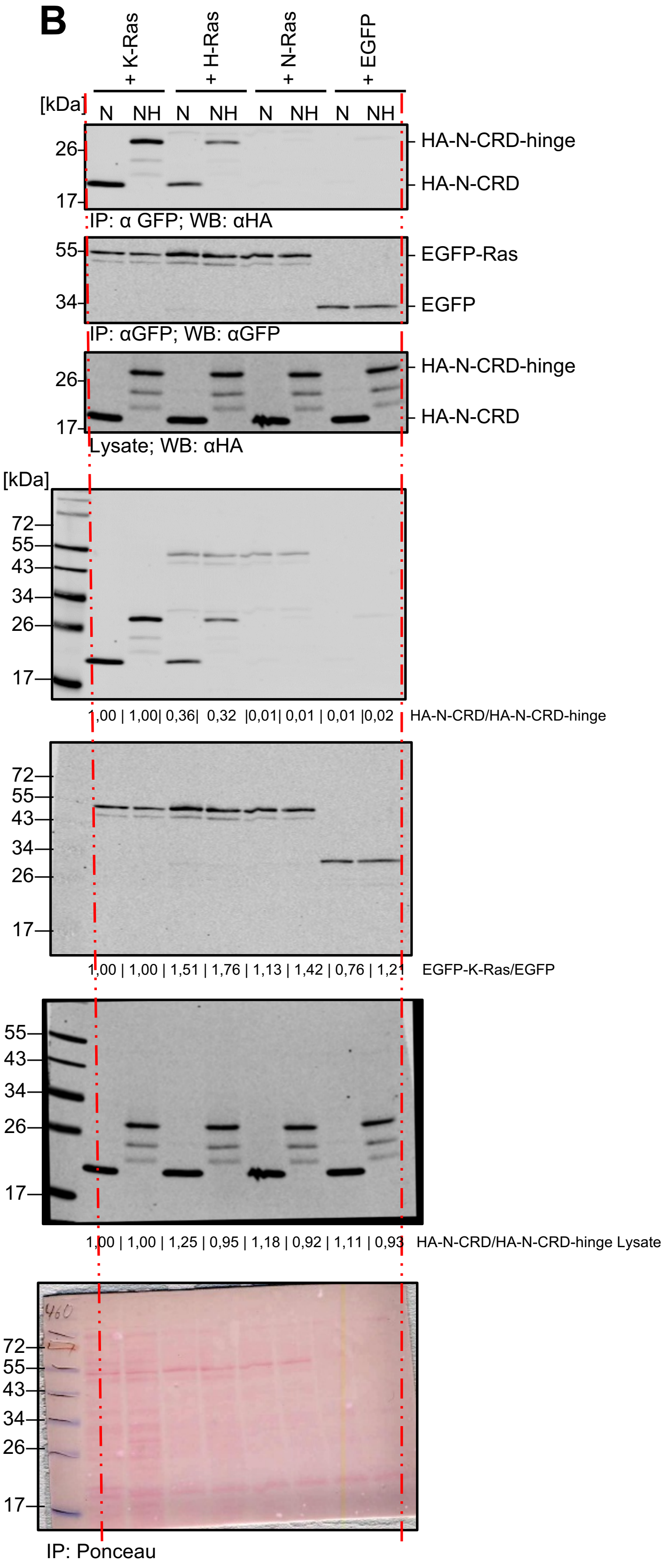
B
+ K-Ras
+ H-Ras
+ N-Ras
+ EGFP
[kDa]
N NH N NH N NH N NH
26
17
HA-N-CRD-hinge
HA-N-CRD
IP: α GFP; WB: αHA
55
34
EGFP-Ras
EGFP
IP: αGFP; WB: αGFP
26
17
HA-N-CRD-hinge
HA-N-CRD
Lysate; WB: αHA
[kDa]
72
55
43
34
26
17
1,00 | 1,00| 0,36| 0,32 |0,01| 0,01 | 0,01 |0,02
HA-N-CRD/HA-N-CRD-hinge
72
55
43
34
26
17
1,00 | 1,00 | 1,51 | 1,76 | 1,13 | 1,42 | 0,76 | 1,21
EGFP-K-Ras/EGFP
55
43
34
26
17
1,00 | 1,00 | 1,25 | 0,95 | 1,18 | 0,92 | 1,11 | 0,93
HA-N-CRD/HA-N-CRD-hinge Lysate
72
55
43
34
26
17
IP: Ponceau

Fig.5

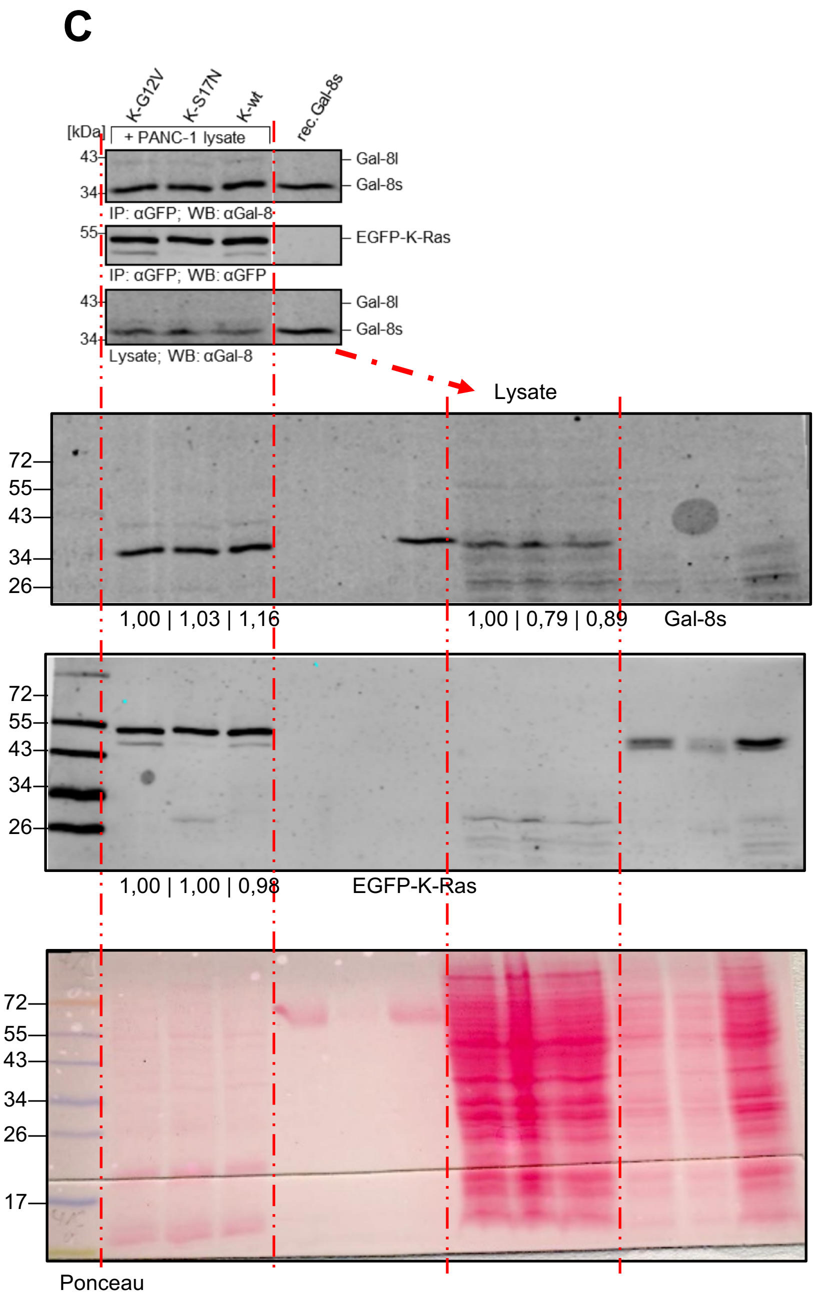

C
K-G12V
K-S17N
K-wt
rec. Gal-8s
[kDa]
+ PANC-1 lysate
43
34
Gal-8l
Gal-8s
IP: αGFP; WB: αGal-8
55
EGFP-K-Ras
IP: αGFP; WB: αGFP
43
34
Gal-8l
Gal-8s
Lysate; WB: αGal-8
Lysate
72
55
43
34
26
1,00 | 1,03 | 1,16
1,00 | 0,79 | 0,89
Gal-8s
72
55
43
34
26
1,00 | 1,00 | 0,98
EGFP-K-Ras
72
55
43
34
26
17
Ponceau

Fig.5 D

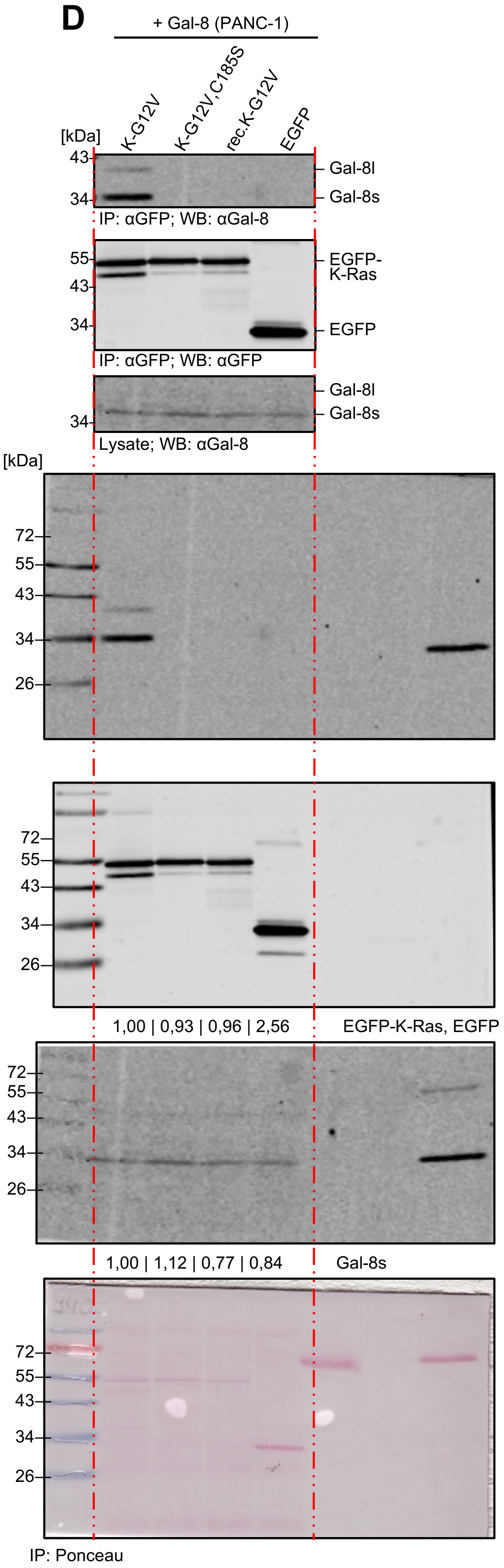
D
+ Gal-8 (PANC-1)
K-G12V
K-G12V,C185S
rec.K-G12V
EGFP
[kDa]
43
34
Gal-8l
Gal-8s
IP: αGFP; WB: αGal-8
55
43
34
EGFP-
K-Ras
EGFP
IP: αGFP; WB: αGFP
34
Gal-8l
Gal-8s
Lysate; WB: αGal-8
[kDa]
72
55
43
34
26
72
55
43
34
26
1,00 | 0,93 | 0,96 | 2,56
EGFP-K-Ras, EGFP
72
55
43
34
26
1,00 | 1,12 | 0,77 | 0,84
Gal-8s
72
55
43
34
26
IP: Ponceau

Fig.5 E

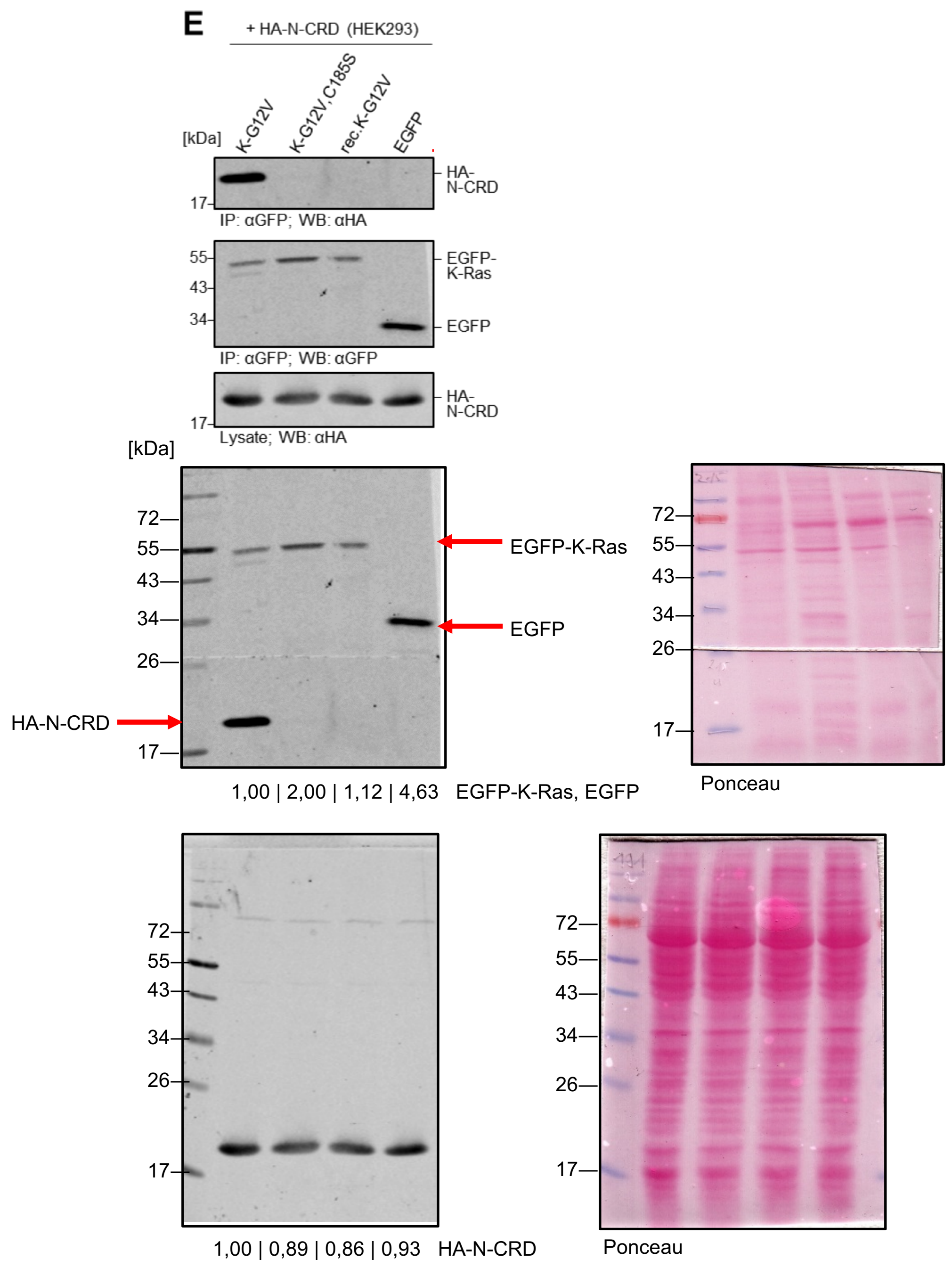
E
+ HA-N-CRD (HEK293)
[kDa]
K-G12V
K-G12V,C185S
rec.K-G12V
EGFP
HA-N-CRD
17
IP: αGFP; WB: αHA
55
43
34
EGFP-K-Ras
EGFP
IP: αGFP; WB: αGFP
17
HA-N-CRD
Lysate; WB: αHA
[kDa]
72
55
43
34
26
17
EGFP-K-Ras
EGFP
HA-N-CRD
1,00 | 2,00 | 1,12 | 4,63 EGFP-K-Ras, EGFP
Ponceau
1,00 | 0,89 | 0,86 | 0,93 HA-N-CRD
Ponceau

Fig.5 F

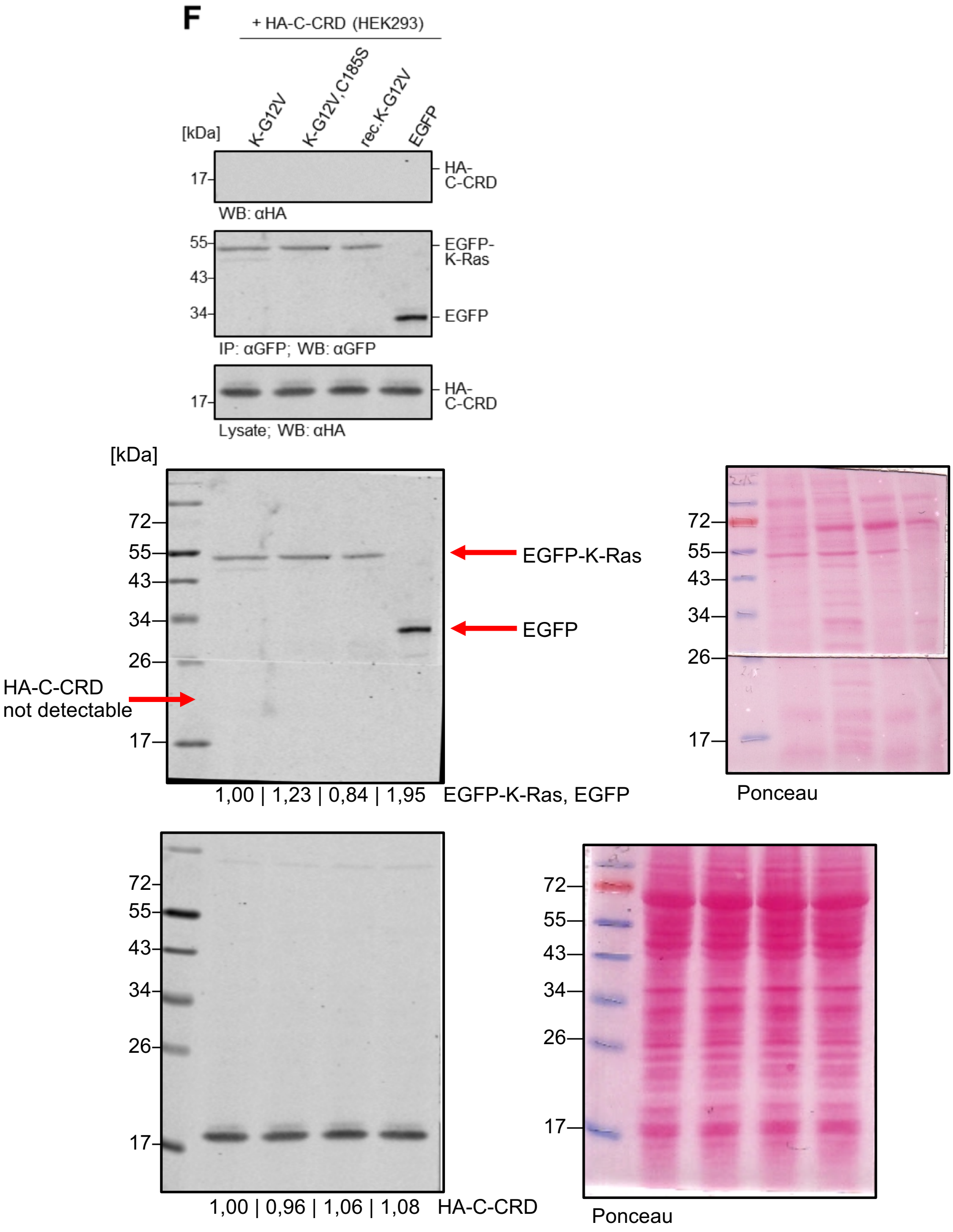
F
+ HA-C-CRD (HEK293)
K-G12V
K-G12V,C185S
rec.K-G12V
EGFP
[kDa]
17
HA-C-CRD
WB: αHA
55
43
34
EGFP-K-Ras
EGFP
IP: αGFP; WB: αGFP
17
HA-C-CRD
Lysate; WB: αHA
[kDa]
72
55
43
34
26
17
EGFP-K-Ras
EGFP
HA-C-CRD not detectable
1,00 | 1,23 | 0,84 | 1,95 EGFP-K-Ras, EGFP
Ponceau
72
55
43
34
26
17
1,00 | 0,96 | 1,06 | 1,08 HA-C-CRD
Ponceau

Fig.6

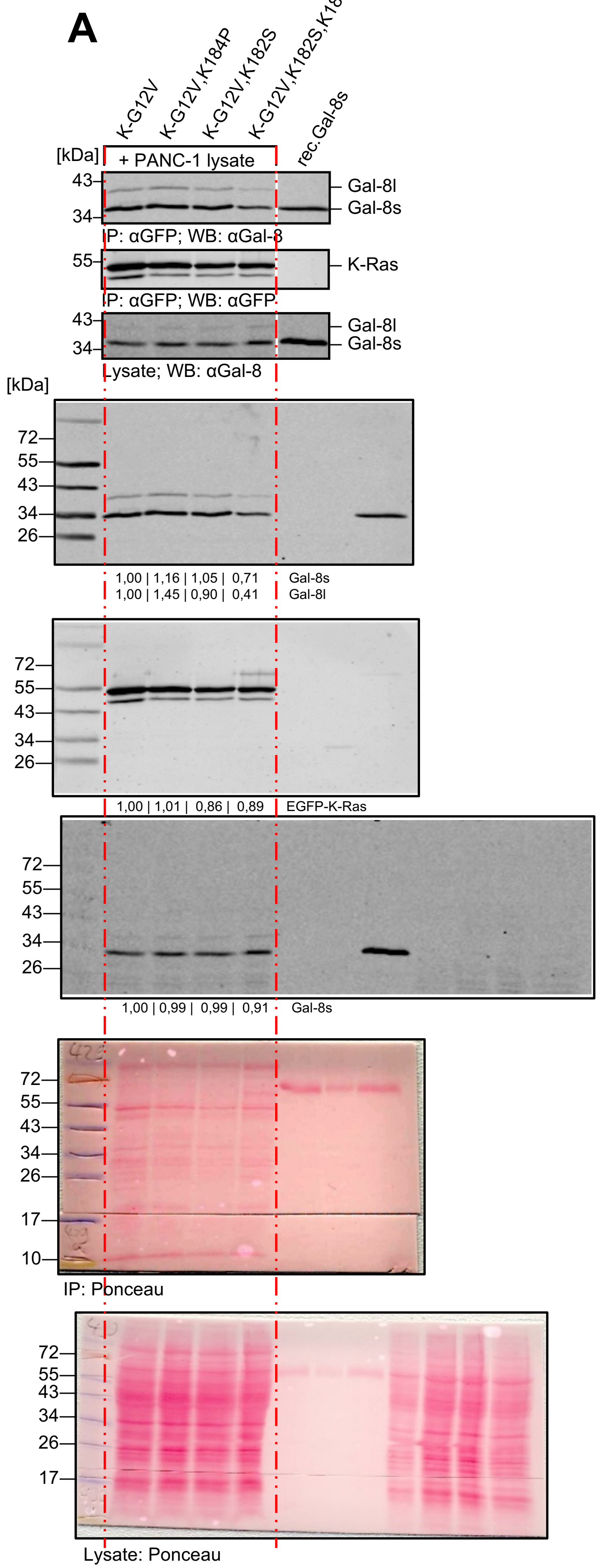
A
K-G12V
K-G12V,K184P
K-G12V,K182S
K-G12V,K182S,K184P
rec.Gal-8s
[kDa]
+ PANC-1 lysate
43
34
Gal-8l
Gal-8s
IP: αGFP; WB: αGal-8
55
K-Ras
IP: αGFP; WB: αGFP
43
34
Gal-8l
Gal-8s
Lysate; WB: αGal-8
[kDa]
72
55
43
34
26
1,00 | 1,16 | 1,05 | 0,71
Gal-8s
1,00 | 1,45 | 0,90 | 0,41
Gal-8l
72
55
43
34
26
1,00 | 1,01 | 0,86 | 0,89
EGFP-K-Ras
72
55
43
34
26
1,00 | 0,99 | 0,99 | 0,91
Gal-8s
72
55
43
34
26
17
10
IP: Ponceau
72
55
43
34
26
17
Lysate: Ponceau

Fig.6

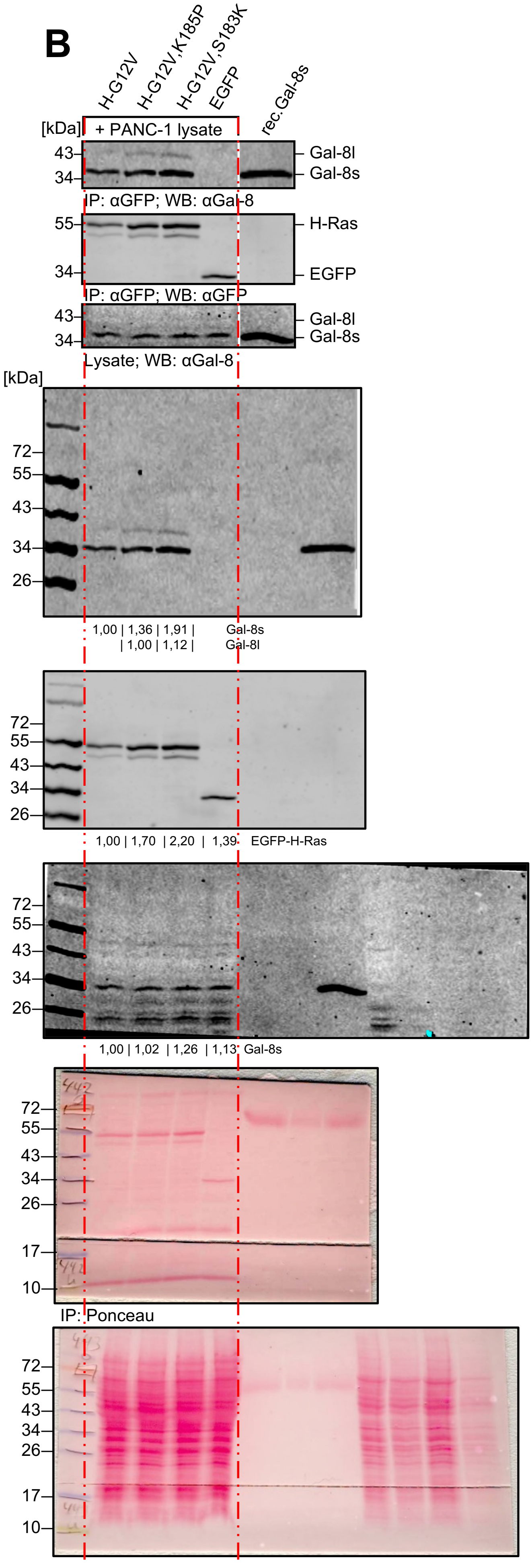
B
H-G12V
H-G12V,K185P
H-G12V,S183K
EGFP
rec.Gal-8s
[kDa]
+ PANC-1 lysate
43
34
Gal-8l
Gal-8s
IP: αGFP; WB: αGal-8
55
34
H-Ras
EGFP
IP: αGFP; WB: αGFP
43
34
Gal-8l
Gal-8s
Lysate; WB: αGal-8
[kDa]
72
55
43
34
26
1,00 | 1,36 | 1,91 |
| 1,00 | 1,12 |
Gal-8s
Gal-8l
72
55
43
34
26
1,00 | 1,70 | 2,20 | 1,39
EGFP-H-Ras
72
55
43
34
26
1,00 | 1,02 | 1,26 | 1,13
Gal-8s
72
55
43
34
26
17
10
IP: Ponceau
72
55
43
34
26
17
10
Lysate: Ponceau

Fig.6

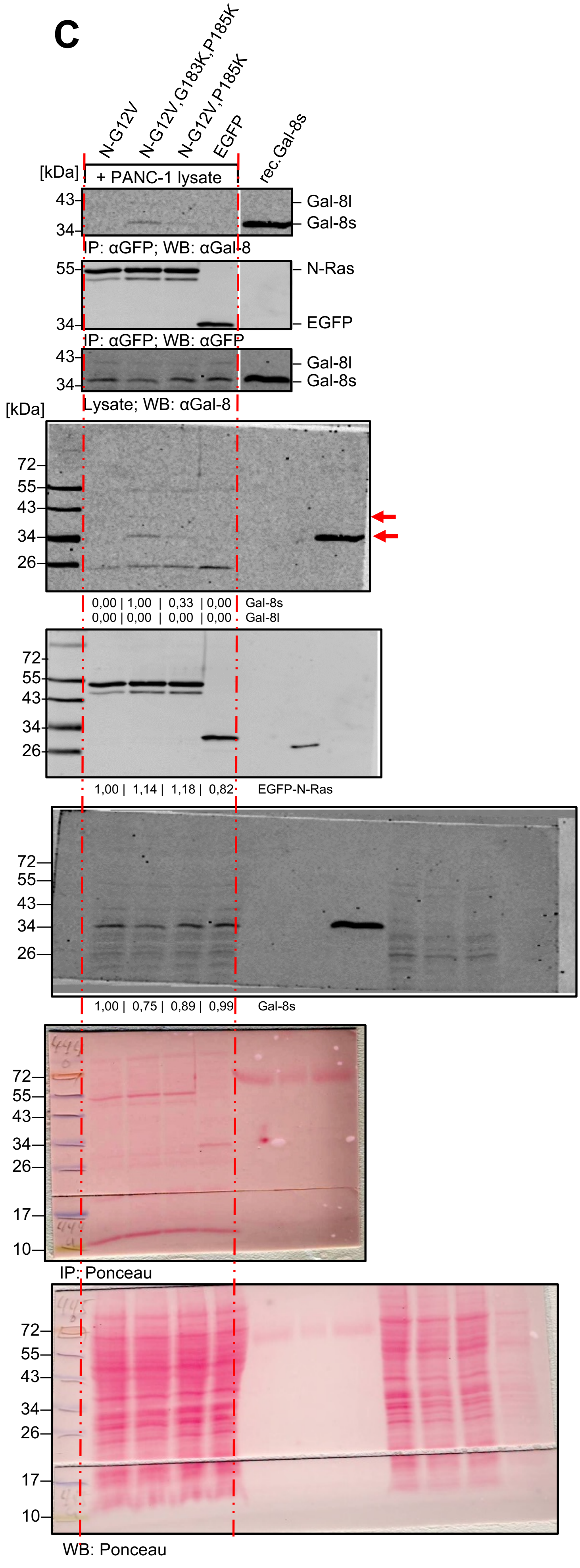
C
N-G12V
N-G12V,G183K,P185K
N-G12V,P185K
EGFP
rec.Gal-8s
[kDa]
+ PANC-1 lysate
43
34
Gal-8l
Gal-8s
IP: αGFP; WB: αGal-8
55
34
N-Ras
EGFP
IP: αGFP; WB: αGFP
43
34
Gal-8l
Gal-8s
Lysate; WB: αGal-8
[kDa]
72
55
43
34
26
0,00 | 1,00 | 0,33 | 0,00 Gal-8s
0,00 | 0,00 | 0,00 | 0,00 Gal-8l
72
55
43
34
26
1,00 | 1,14 | 1,18 | 0,82 EGFP-N-Ras
72
55
43
34
26
1,00 | 0,75 | 0,89 | 0,99 Gal-8s
72
55
43
34
26
17
10
IP: Ponceau
72
55
43
34
26
17
10
WB: Ponceau

Fig.7

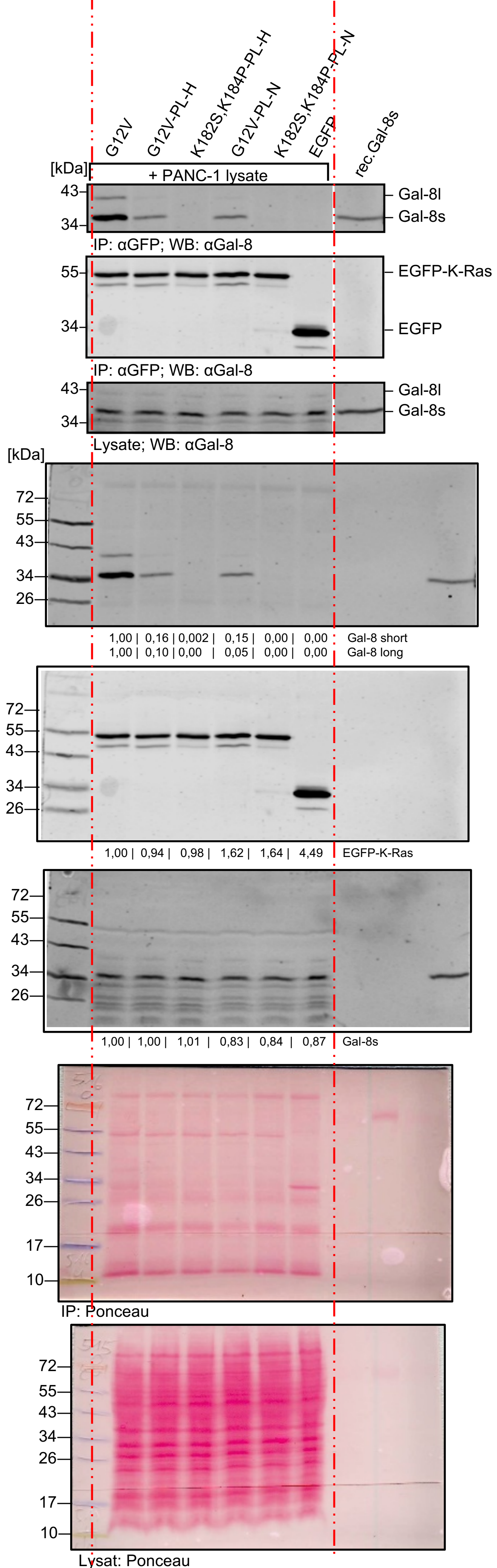
G12V
G12V-PL-H
K182S,K184P-PL-H
G12V-PL-N
K182S,K184P-PL-N
EGFP
rec.Gal-8s
[kDa]
+ PANC-1 lysate
Gal-8l
Gal-8s
IP: αGFP; WB: αGal-8
EGFP-K-Ras
EGFP
IP: αGFP; WB: αGal-8
Gal-8l
Gal-8s
Lysate; WB: αGal-8
1,00 | 0,16 | 0,002 | 0,15 | 0,00 | 0,00 Gal-8 short
1,00 | 0,10 | 0,00 | 0,05 | 0,00 | 0,00 Gal-8 long
1,00 | 0,94 | 0,98 | 1,62 | 1,64 | 4,49 EGFP-K-Ras
1,00 | 1,00 | 1,01 | 0,83 | 0,84 | 0,87 Gal-8s
IP: Ponceau
Lysat: Ponceau

Fig.S1

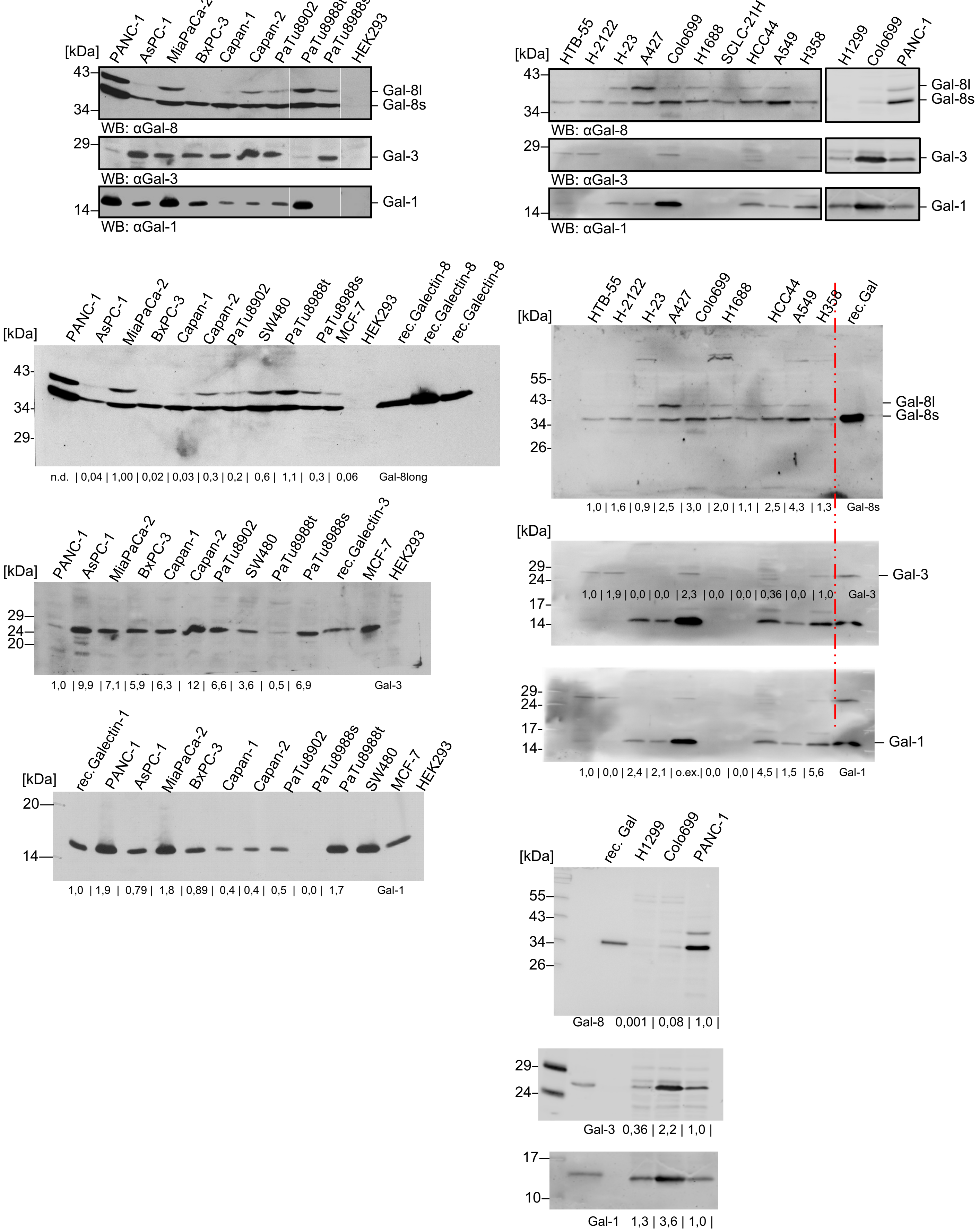
[kDa]
PANC-1 AsPC-1 MiaPaCa-2 BxPC-3 Capan-1 Capan-2 PaTu8902 PaTu8988t PaTu8988s HEK293
43 34 Gal-8l Gal-8s
WB: αGal-8
29 Gal-3
WB: αGal-3
14 Gal-1
WB: αGal-1
HTB-55 H-2122 H-23 A427 Colo699 H1688 SCLC-21H HCC44 A549 H358 H1299 Colo699 PANC-1
43 34 Gal-8l Gal-8s
WB: αGal-8
29 Gal-3
WB: αGal-3
14 Gal-1
WB: αGal-1
PANC-1 AsPC-1 MiaPaCa-2 BxPC-3 Capan-1 Capan-2 PaTu8902 SW480 PaTu8988t PaTu8988s MCF-7 HEK293 rec.Galectin-8 rec.Galectin-8 rec.Galectin-8
43 34 29
n.d. | 0,04 | 1,00 | 0,02 | 0,03 | 0,3 | 0,2 | 0,6 | 1,1 | 0,3 | 0,06 Gal-8long
HTB-55 H-2122 H-23 A427 Colo699 H1688 HCC44 A549 H358 rec.Gal
55 43 34 26 Gal-8l Gal-8s
1,0 | 1,6 | 0,9 | 2,5 | 3,0 | 2,0 | 1,1 | 2,5 | 4,3 | 1,3 Gal-8s
PANC-1 AsPC-1 MiaPaCa-2 BxPC-3 Capan-1 Capan-2 PaTu8902 SW480 PaTu8988t PaTu8988s rec.Galectin-3 MCF-7 HEK293
29 24 20
1,0 | 9,9 | 7,1 | 5,9 | 6,3 | 12 | 6,6 | 3,6 | 0,5 | 6,9 Gal-3
29 24 17 14 Gal-3
1,0 | 1,9 | 0,0 | 0,0 | 2,3 | 0,0 | 0,0 | 0,36 | 0,0 | 1,0 Gal-3
29 24 17 14 Gal-1
1,0 | 0,0 | 2,4 | 2,1 | o.ex. | 0,0 | 0,0 | 4,5 | 1,5 | 5,6 Gal-1
rec.Galectin-1 PANC-1 AsPC-1 MiaPaCa-2 BxPC-3 Capan-1 Capan-2 PaTu8902 PaTu8988s PaTu8988t SW480 MCF-7 HEK293
20 14
1,0 | 1,9 | 0,79 | 1,8 | 0,89 | 0,4 | 0,4 | 0,5 | 0,0 | 1,7 Gal-1
rec. Gal H1299 Colo699 PANC-1
55 43 34 26
Gal-8 0,001 | 0,08 | 1,0 |
29 24
Gal-3 0,36 | 2,2 | 1,0 |
17 10
Gal-1 1,3 | 3,6 | 1,0 |

Fig.S2

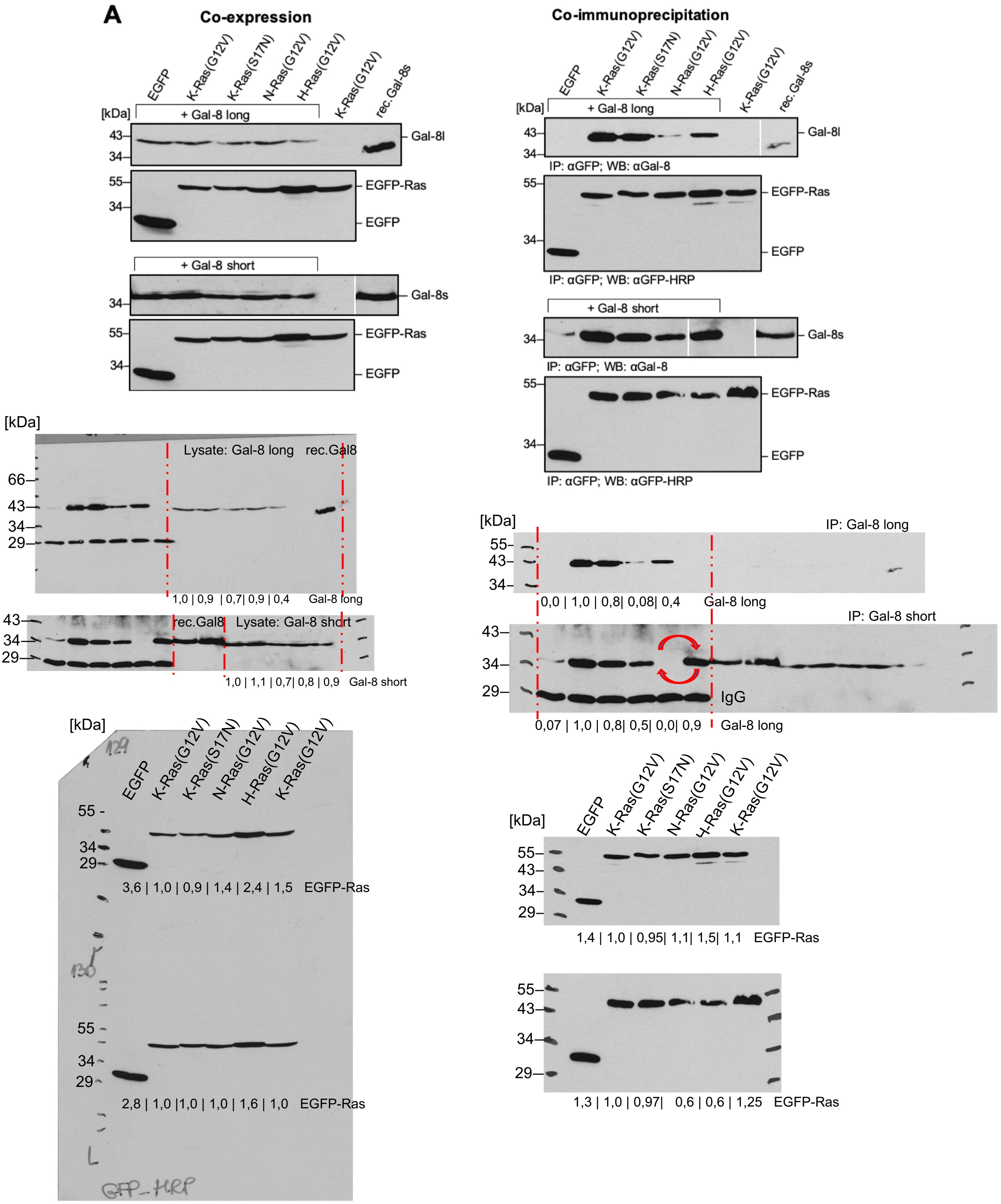
A
Co-expression
EGFP
K-Ras(G12V)
K-Ras(S17N)
N-Ras(G12V)
H-Ras(G12V)
K-Ras(G12V)
rec.Gal-8s
[kDa]
+ Gal-8 long
Gal-8l
EGFP-Ras
EGFP
+ Gal-8 short
Gal-8s
Co-immunoprecipitation
IP: αGFP; WB: αGal-8
IP: αGFP; WB: αGFP-HRP
Lysate: Gal-8 long
rec.Gal8
1,0 | 0,9 | 0,7| 0,9 | 0,4
Gal-8 long
Lysate: Gal-8 short
1,0 | 1,1 | 0,7| 0,8 | 0,9
Gal-8 short
IP: Gal-8 long
0,0 | 1,0 | 0,8| 0,08| 0,4
IP: Gal-8 short
IgG
0,07 | 1,0 | 0,8| 0,5| 0,0| 0,9
3,6 | 1,0 | 0,9 | 1,4 | 2,4 | 1,5
2,8 | 1,0 |1,0 | 1,0 | 1,6 | 1,0
GFP-HRP
1,4 | 1,0 | 0,95| 1,1| 1,5| 1,1
1,3 | 1,0 | 0,97| 0,6 | 0,6 | 1,25

Fig.S2

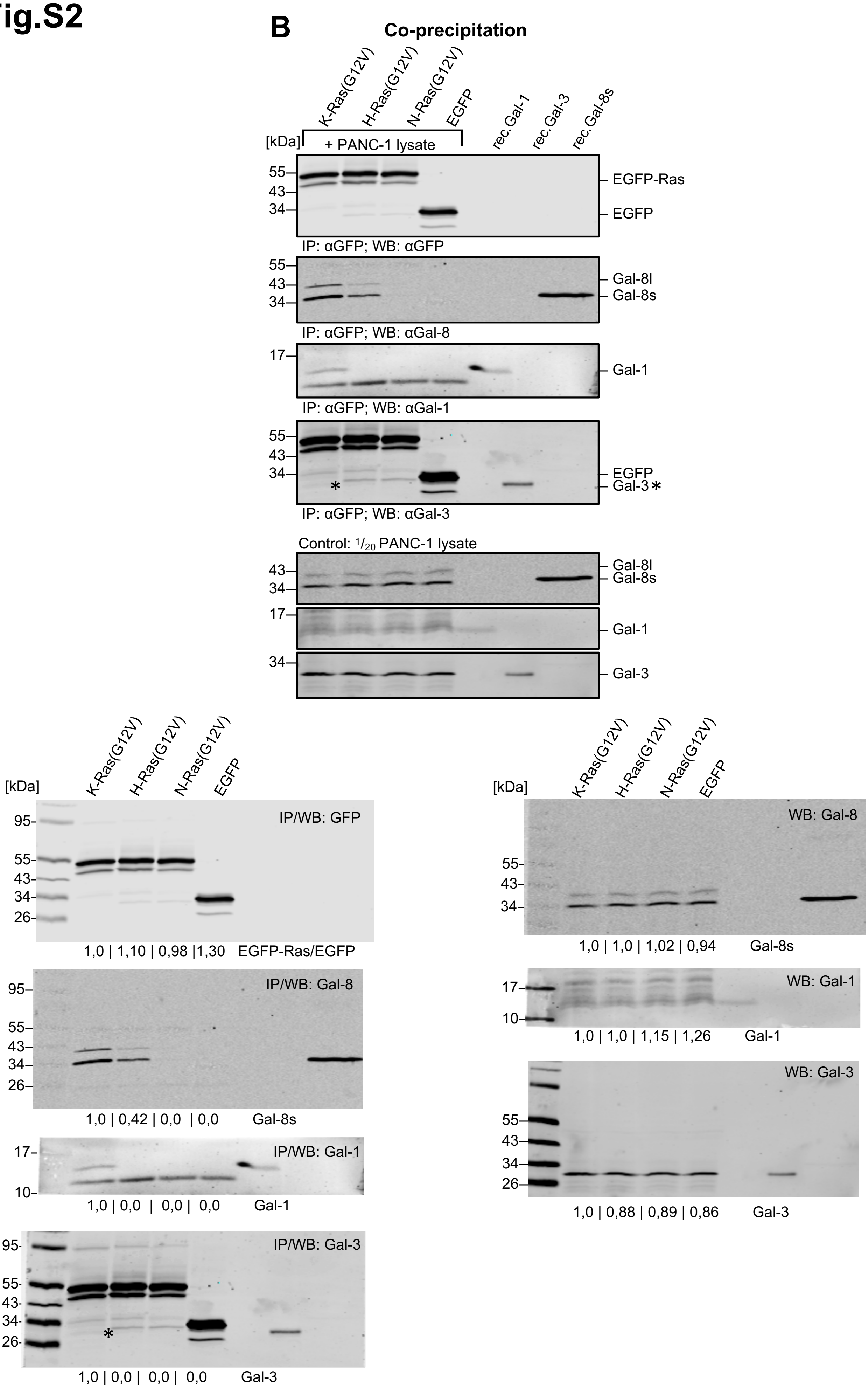
B
Co-precipitation
K-Ras(G12V)
H-Ras(G12V)
N-Ras(G12V)
EGFP
rec.Gal-1
rec.Gal-3
rec.Gal-8s
[kDa]
+ PANC-1 lysate
55
43
34
EGFP-Ras
EGFP
IP: αGFP; WB: αGFP
Gal-8l
Gal-8s
IP: αGFP; WB: αGal-8
17
Gal-1
IP: αGFP; WB: αGal-1
Gal-3
*
IP: αGFP; WB: αGal-3
Control: 1/20 PANC-1 lysate
IP/WB: GFP
95
26
1,0 | 1,10 | 0,98 |1,30
EGFP-Ras/EGFP
IP/WB: Gal-8
1,0 | 0,42 | 0,0 | 0,0
Gal-8s
IP/WB: Gal-1
10
1,0 | 0,0 | 0,0 | 0,0
Gal-1
IP/WB: Gal-3
1,0 | 0,0 | 0,0 | 0,0
Gal-3
WB: Gal-8
1,0 | 1,0 | 1,02 | 0,94
Gal-8s
WB: Gal-1
1,0 | 1,0 | 1,15 | 1,26
Gal-1
WB: Gal-3
1,0 | 0,88 | 0,89 | 0,86
Gal-3

Fig.S3

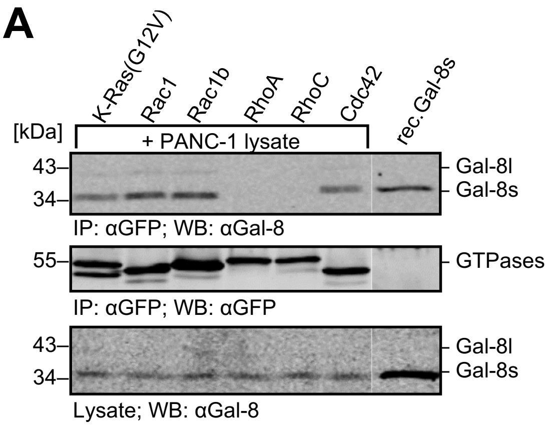
A
K-Ras(G12V)
Rac1
Rac1b
RhoA
RhoC
Cdc42
rec.Gal-8s
[kDa]
+ PANC-1 lysate
43–
34–
Gal-8l
Gal-8s
IP: αGFP; WB: αGal-8
55–
GTPases
IP: αGFP; WB: αGFP
43–
34–
Gal-8l
Gal-8s
Lysate; WB: αGal-8

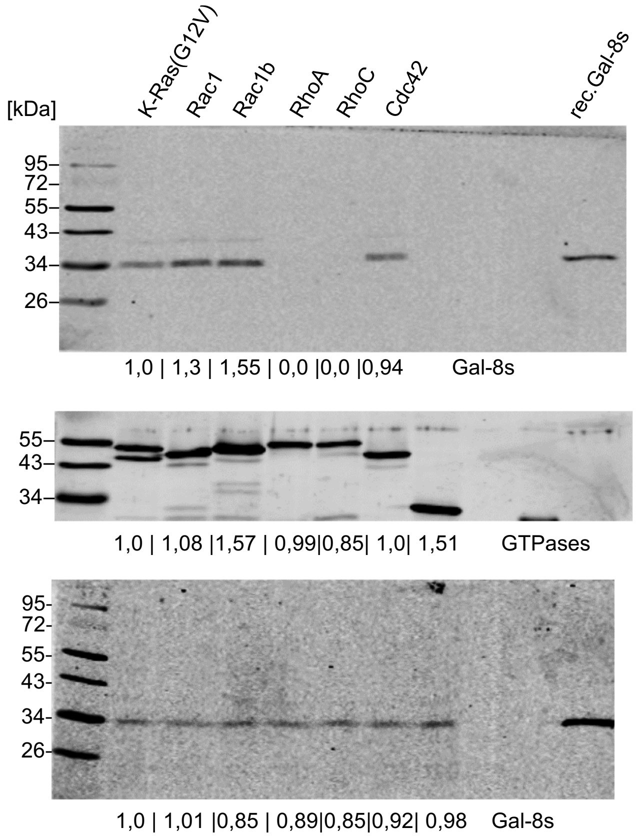
K-Ras(G12V)
Rac1
Rac1b
RhoA
RhoC
Cdc42
rec.Gal-8s
[kDa]
95–
72–
55–
43–
34–
26–
1,0 | 1,3 | 1,55 | 0,0 |0,0 |0,94
Gal-8s
55–
43–
34–
1,0 | 1,08 |1,57 | 0,99|0,85| 1,0| 1,51
GTPases
95-
72-
55-
43-
34-
26-
1,0 | 1,01 |0,85 | 0,89|0,85|0,92| 0,98
Gal-8s

Fig.S3

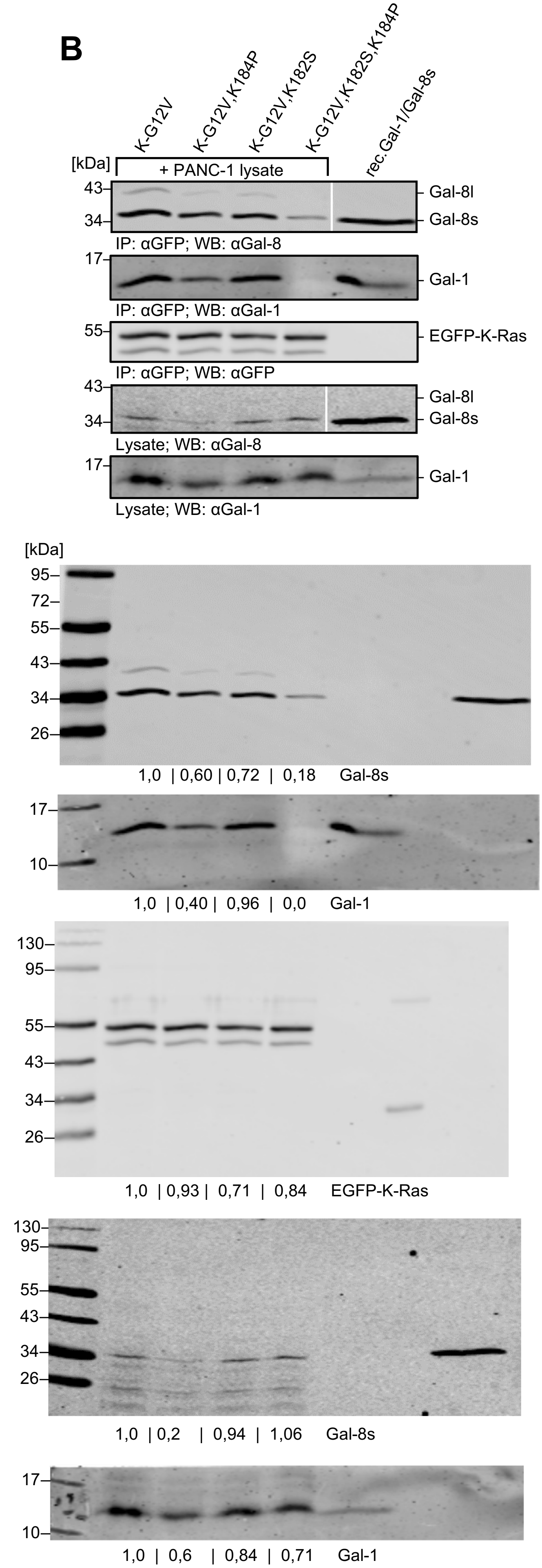
B
K-G12V
K-G12V,K184P
K-G12V,K182S
K-G12V,K182S,K184P
rec.Gal-1/Gal-8s
[kDa]
+ PANC-1 lysate
43
34
Gal-8l
Gal-8s
IP: αGFP; WB: αGal-8
17
Gal-1
IP: αGFP; WB: αGal-1
55
EGFP-K-Ras
IP: αGFP; WB: αGFP
43
34
Gal-8l
Gal-8s
Lysate; WB: αGal-8
17
Gal-1
Lysate; WB: αGal-1
[kDa]
95
72
55
43
34
26
1,0 | 0,60 | 0,72 | 0,18 Gal-8s
17
10
1,0 | 0,40 | 0,96 | 0,0 Gal-1
130
95
55
43
34
26
1,0 | 0,93 | 0,71 | 0,84 EGFP-K-Ras
130
95
55
43
34
26
1,0 | 0,2 | 0,94 | 1,06 Gal-8s
17
10
1,0 | 0,6 | 0,84 | 0,71 Gal-1
